# Passivity-based Rieman Liouville fractional order sliding mode control of three phase inverter in a grid-connected photovoltaic system

**DOI:** 10.1371/journal.pone.0296797

**Published:** 2024-02-07

**Authors:** Luqman Khan, Laiq Khan, Shahrukh Agha, Kamran Hafeez, Jamshed Iqbal

**Affiliations:** 1 Department of Electrical and Computer Engineering, COMSATS University Islamabad, Islamabad, Pakistan; 2 School of Computer Science, Faculty of Science and Engineering, University of Hull, Hull, United Kingdom; Vellore Institute of Technology, INDIA

## Abstract

Photovoltaic (PV) system parameters are always non-linear due to variable environmental conditions. The Maximum power point tracking (MPPT) is difficult under multiple uncertainties, disruptions and the occurrence of time-varying stochastic conditions. Therefore, Passivity based Fractional order Sliding-Mode controller (PBSMC) is proposed to examine and develop a storage function in error tracking for PV power and direct voltage in this research work. A unique sliding surface for Fractional Order Sliding Mode Control (FOSMC) framework is proposed and its stability and finite time convergence is proved by implementing Lyapunov stability method. An additional input of sliding mode control (SMC) is also added to a passive system to boost the controller performance by removing the rapid uncertainties and disturbances. Therefore, PBSMC, along with globally consistent control efficiency under varying operating conditions is implemented with enhanced system damping and substantial robustness. The novelty of the proposed technique lies in a unique sliding surface for FOSMC framework based on Riemann Liouville (R-L) fractional calculus. Results have shown that the proposed control technique reduces the tracking error in PV output power, under variable irradiance conditions, by 81%, compared to fractional order proportional integral derivative (FOPID) controller. It is reduced by 39%, when compared to passivity based control (PBC) and 28%, when compared to passivity based FOPID (EPBFOPID). The proposed technique led to the least total harmonic distortion in the grid side voltage and current. The tracking time of PV output power is 0.025 seconds in PBSMC under varying solar irradiance, however FOPID, PBC, EPBFOPID, have failed to converge fully. Similarly the dc link voltage has tracked the reference voltage in 0.05 seconds however the rest of the methods either could not converge, or converged after significant amount of time. During solar irradiance and temperature change, the photovoltaic output power has converged in 0.018 seconds using PBSMC, however remaining methods failed to converge or track fully and the dc link voltage has minimum tracking error due to PBSMC as compared to the other methods. Furthermore, the photovoltaic output power converges to the reference power in 0.1 seconds in power grid voltage drop, whereas other methods failed to converge fully. In addition power is also injected from the PV inverter into the grid at unity power factor.

## 1. Introduction

A strong electrical power system becomes the primary element for the growth and progress of a nation, as quality of life of citizens, agricultural-industrial development, and levels of production depends on a continuous supply of electricity [1].

Due to quick industrialization and rapid growth of the world population, fossil fuels like oil, gas, and coal reserves are declining. Sustainable and endless technologies for clean energy to produce electricity are also desperately needed to completely meet the ever-increasing demand for energy, which is projected to produce approximately half of all growth in 2040 [2]. Electricity harvested from various natural resources (hydropower, solar, wind, tidal, biomass, geothermal, bio fuel) leads to development of different modern technologies [3]. Among these, one of the main constructive sources of sustainability is solar energy which has cleanliness, time distribution, and merits of abundance. Due to high advantages of solar energy such as low operational cost, less maintenance, no carbon emissions, no moving parts, silent energy production (making no noise in energy conversion), and more than 20 years of its long lifetime, it has gained numerous interests and attention in academic and industrial sectors. Increased energy costs and environmental restrictions are motivated by the advancement of technology solutions that allow improved resource management and the utilization of renewable energies by unique photovoltaic energy sources [4]. The PV cell is the fundamental unit and vital part of a photovoltaic system and its output power is subjected to illumination, temperature of photoelectric material, and component aging, etc. The productivity distinctiveness of photovoltaic cells is altering with the change of environmental factors which is always non-linear [5]. The photovoltaic has seen an increase in efficiency because of the innovations of the solar cells. Practically, it is very complex to always track the maximum available power from PV systems and to make use of the PV cell more efficiently. The extraction of maximum power process is called MPPT [6]. To obtain the maximum power output under different atmospheric conditions, there are different algorithms i.e. hill climbing [7], Perturb and observe (P&O) [8], and Incremental conductance (INC) [9]. The DC voltage is regulated by PV inverter that converts it into single-phase or 3-phase AC currents. The methods mentioned above are unsuccessful in tracking the MPP under speedily altering atmospheric surroundings and the operating point oscillates at steady-state, resulting in power loss [10]. To improve MPPT accuracy and speed, [11] suggested the variable step size INC-MPPT method. However, stability cannot be ensured by the above described MPPT processes. Then advanced MPPT techniques are suggested in [12, 13], based on ripples association control (RAC). Such methods have demonstrated good efficiency, while a reliable operation can be assured at the same instant of time. Therefore, a correct device configuration of PV inverters is very imperative to achieve an accurate and proficient MPPT.

Proportional integral derivative (PID) along with Vector- control loops are traditionally used for PV inverter due to its currently aerial operational performance and its simple structure [14]. Nevertheless, it cannot achieve reliable control efficiency under varying operational conditions. To boost traditional control efficiency for PID control, [15] suggested a minimum power control technique with a half-order PID controller based on unknown parameters of PV. Meanwhile, optimized fractional-order PI (FOPI) control for the solar photovoltaic systems have been developed [16]. Above mentioned methods depend on a fractional-order estimation, that provides two additional fractional order parameters to change the device dynamics further. However, the later methods have the underlying flaws of linear control, such as the one-point linearization of original nonlinear system. Many nonlinear control schemes are used to ensure global control consistency, aimed at ensuring adequate control performance for various purposes. Feedback linearization control (FLC) was suggested in the work of [17] for grid-connected PV three-level inverters, in which nonlinearities of the PV inverter were eliminated to realize a global continuity of control with different operating conditions. But a stable PV device model is needed because it is highly vulnerable to any kind of uncertainties or external perturbations of parameters. An MPPT controller was also designed to increase tracking accuracy at different solar irradiance and temperature levels in [18]. Furthermore [19] suggested an advanced Adaptive SMC approach, to discard inconsistencies and instability, which was capable of properly boosting the forcefulness of the PV device. In [20], a perturbation estimator has been suggested to reduce exposure to the unstable parameters and to easily reject grid side disruption in a digital predictive current control controller. In general, the above techniques treat the PV systems regulation as a mathematical challenge, the necessary and important physical characteristics for the complicated responses of the system are in some cases not fully analyzed and are ignored. The Lyapunov stability theorem flexibly decomposes a complex initial structure into many sub-systems [21] with an effective storage mechanism, to reorganize the total power of the closed-loop system by inserting distributed energies. The principle of energy determination based on its reshaping for dynamic control of manipulator was given in [22]. The control issues were then balanced and the time variants of the storage space mechanism took the preferred form to construct a correct sequence of links between the controller and the dynamic system under consideration [23].

Passivity-based controls (PBCs) are also highly promising for PV control architecture since they can be used as a power transfer unit. Two types of PBC schemes are widespread in literature: interconnection, damp passivity-based control (IDAPBC) [24] and proportional-integral passivity-based control (PIPBC) [25]. These two approaches were proposed by [1] for the constancy analysis of hydro-solar power systems. PIPBC was a model for bilinear model incorporated by electronic power converters. To obtain fast and accurate photovoltaic systems in terms of environmental changes a passivity-based MPPT controller was developed in [5] for grid-connected PV systems. PBCs with damping techniques and energy shaping methods of injectors for the power modulation PV/battery hybrid power sources have been synthesized in [26]. Similarly, [27] applied a passivity-based control assumption to control the current control mode of a battery energy storage device under closed-loop conditions of global exponential stability. Meanwhile, an algebraic identification parameter was employed to approximate unknown PV array voltage, battery voltage, and charge resistance parameters via the PV/battery hybrid energy sources adaptive passivity-based controller (APBC) [28]. A PBC was engineered with Euler-Lagrange (EL) damping in order to enhance the complex output of the electricity-related current with the T-type neutral point clamped PV inverter [2]. In comparison, a PBC was suggested for the PV inverter revealing strong reference tracking with fast dynamics to ensure that the tracking error asymptotically became zero. Thus Passivity based control (PBC) showed more robustness during parameters disturbances, and can be easily implemented [29].

It has been well established that the utilization of sliding mode control (SMC) leads to the development of robust controllers for complex non-linear dynamic plants which are operating under different uncertain conditions. It is less sensitive to the variations in plant parameters and disturbances due to which the exact modeling of the plant becomes unnecessary. However, SMC has a chattering phenomenon, which is undesirable. Conventional SMC cannot control these oscillations due to chattering within the bounded time, however fractional order SMC has the ability to reduce the amplitude of the error signal and conversion time by selecting appropriate fractional coefficient ‘*α*’.

Sliding-mode control (SMC) have several applications in different areas and can model uncertainties and disturbances. A fractional order sliding mode control (FOSMC) is used to reduce chattering in a current and power signals. It gradually suppresses signal chattering in a lower order system using traditional sliding mode control (SMC) [30–34]. In [35], a magnetic suspension system of a low speed maglev train was suggested along with the implementation of magnetic suspension controller and a nonlinear mathematical model of the magnetic suspension system. In this work PID controller was considered but it was sensitive to disturbances. To remove disturbances and parameter perturbations an adaptive neural-fuzzy sliding mode controller was suggested, based on sliding mode control, adaptive-fuzzy approximator, and the neural-fuzzy switching law. It efficiently reduced the impact of the disturbance and parameter perturbations.

In [36], an adaptive neural network controller comprising input delay compensation and a control parameter optimization scheme was presented for the electromagnetic levitation system of a maglev vehicle. It was robust against the issues of external disturbance, input time delay, and time-varying mass. A sliding-mode surface with time-delay compensation was implemented for the problem of input time delay, a double-layer neural network and adaptive laws were implemented, leading to adaptive tracking control law with finite time. In addition, the stability of the proposed controller in finite time, using Lyapunov stability method, was analyzed that enhanced the robustness of the system.

In [37], authors have presented fuzzy supervisory passivity-based high order-sliding mode control approach for tidal turbine-based permanent magnet synchronous generator conversion system. They reported problems associated with conventional control techniques such as PI control related to machine side. They emphasized the advantage of passivity based control and mentioned that instead of the cancellation of the nonlinear properties, they were damped, leading to more robustness and stability. In a passivity based control, the system’s natural energy was reshaped and damping was injected in a controlled manner, to achieve the desired system dynamics. A hybrid controller law was implemented by combining high order sliding mode control and passivity based control to achieve robustness irrespective of uncertainities.

In [38], authors have presented robust interconnection and damping assignment energy-based control for a permanent magnet synchronous motor using high order sliding mode approach and nonlinear observer. They mentioned the benefits of nonlinear controls for the compensation of nonlinearities, external disturbances and parametric fluctuations. A new interconnection and damping assignment passivity based control was proposed which was robust.

In [39] authors have presented advantages of the passivity based control in dynamic voltage restorers for power quality improvement. They reported that under transient and steady state conditions, the passivity based control provided better performance than PI control, e.g. faster transient response, did not generate overshoots and led to zero tracking error of any reference with linear and nonlinear loads. On the other hand with PI control zero steady state error was not achieved and the stability was confined to one operating point.

In [40] authors have presented interconnection and damping assignment passivity-based control as a survey. As mentioned above, the passivity based control rendered a system passive with respect to a storage function and injecting damping. Typically the controller rendered the storage function non-increasing.

In [41], authors have presented design of passivity-based damping controller for suppressing power oscillations in dc microgrids. They mentioned the sensitivity of conventional passivity based control to load variations and suggested the use of interconnection and damping assignment passivity based control in order to overcome the issue. In [42] authors have presented an approach to suppress low frequency oscillation in the traction network of high-speed railway using passivity-based control, i.e. interconnection and damping assignment passivity based control was utilized. They concluded that passivity based control was better than PI control, which was the most important factor for the creation of low frequency oscillations of the high speed railway traction network, in terms of static and dynamic performance and effectively suppressed low frequency oscillations of the high speed railway traction network.

Sliding mode control has been considered as one of the robust controllers for complex high order nonlinear dynamic plants which were operating under uncertain conditions [43]. However sliding mode control also suffers from an undesirable chattering phenomena due to system’s unmodeled dynamics or discrete time implementations, as mentioned above. Chattering is a type of high frequency switching which can induce unwanted dynamics in the system which can destabilize, degrade, or even destroy the system under study. Authors in [43] have presented chattering suppression methods in sliding mode control systems. They suggested an observer based chattering suppression mechanism.

In [44], sliding mode control versus fractional-order sliding mode control, applied to a magnetic levitation system was proposed. Authors showed that, in terms of tracking accuracy, speed of response and chattering, the performance of fractional order sliding mode control was better than sliding mode control. The reason was that due to adjustable fractional orders of derivatives and integrals, more degrees of freedom could be added to the controller. Similarly in [45], a fractional-order sliding mode control method for a class of integer-order nonlinear systems was presented. A fractional order stability theorem was derived. Based on this a novel fractional order sliding surface was proposed and a control law based on it for a class of integer order nonlinear system was derived. The advantage of the proposed technique was that fractional order sliding mode control can be applied to integer order nonlinear systems which are more practical. During solar irradiance change and/or temperature change, the maximum power point of the photovoltaic system shifts. If this maximum power point is not tracked accurately and immediately, it leads to decrease in power output and efficiency of the system. Similarly, during grid voltage drop, the photo voltaic inverter should keep on providing the desired power to the grid. The motivation of the research is to propose robust and fast control system, to track the maximum power point efficiently. The innovation of the paper lies in proposing a hybrid robust and fast control system based on passivity and fractional order sliding mode control. A unique sliding surface for fractional order sliding mode control has been proposed which ensures asymptotic convergence of the error signal without chattering. This has been verified through Lyapunov stability criteria and three test cases, i.e. the performance of the controlled photovoltaic system under irradiance change, under both irradiance and temperature change and under power grid voltage drop.

In [31] a passivity based FOSMC was designed for a grid connected PV system. However inverter was not operated under unity power factor. Whereas in [32] passivity based fractional order PID (PBFOPID) was also studied for a grid connecting PV system. Keeping all these advantages in view, i.e. the benefits of PBC and robustness in FOSMC, a passivity-based fractional order sliding mode controller (PBSMC) is proposed and implemented in this research work.

The key contributions in this work given as:

1. A storage function is developed and the physical characteristics of all its terms are carefully investigated and completely analyzed.
2. Unlike [31], a unique sliding surface for FOSMC framework is proposed based on Riemann Liouville (R-L) fractional calculus.
3. The stability as well as finite time convergence of FOSMC is proved by using Lyapunov stability criteria.
4. FOSMC is implemented as an input to passivize a system by reshaping its storage function. It significantly increases the robustness of a PV inverter during parameter uncertainties.
5. The proposed PBSMC is compared with the existing latest techniques, i.e. Fractional order PID (FOPID) [32], Passivity based fractional order PID (PBFOPID) [32] and PBC [5, 31], under three cases a) irradiance change b) irradiance and temperature change c) power grid voltage drop.

## 2. Grid-connected PV inverter modeling

**Fig 1:**
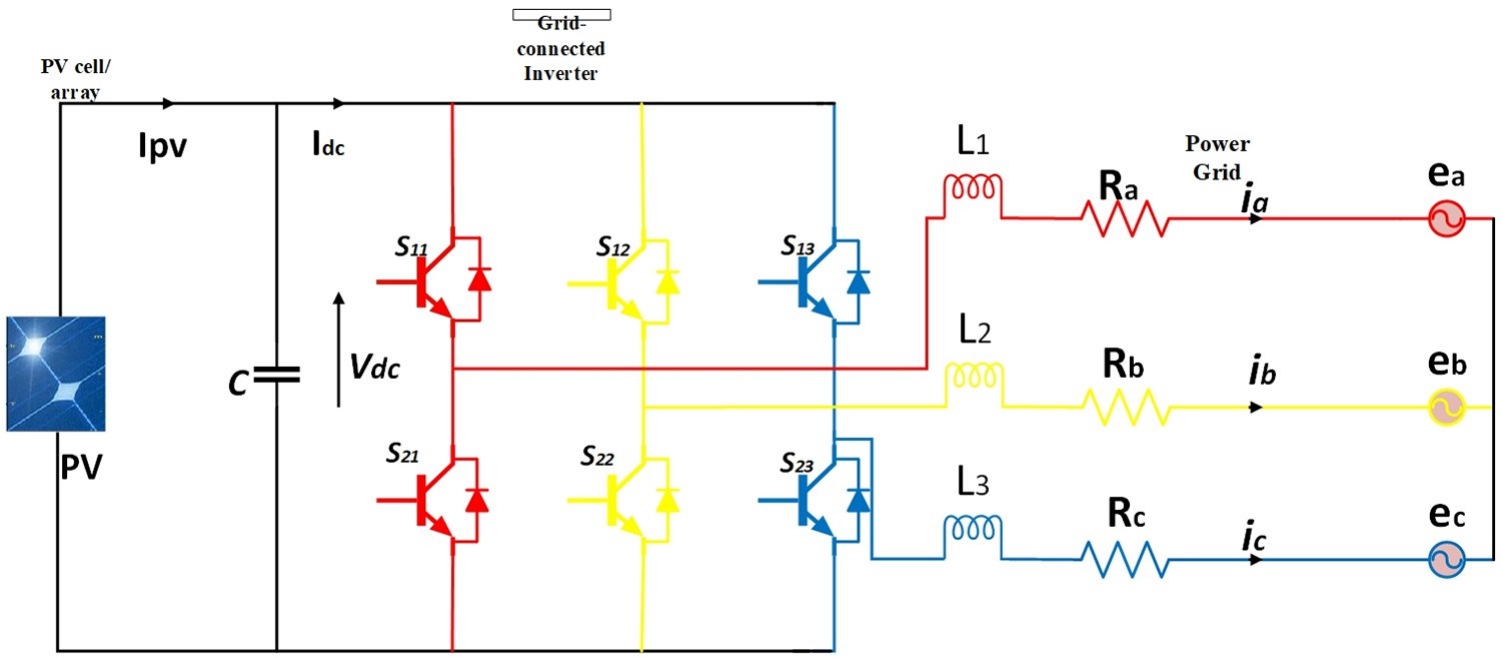
Grid-connected PV inverter.

In Fig 1 grid-connected system with three-phase inverter is shown. The structure includes different components. The conversion of solar irradiance to DC current occurs in PV cell, DC linked capacitor is used to diminish frequency ripples in the DC voltage of the PV inverter. The PV inverter is connected to the dc-link capacitor by converting input DC power into AC power. An R-L filter and 3-phase power grid is also connected [46].

PV cells are connected together to form PV modules. Each PV cell contains a luminosity emitted current generated source, a series resistance, with a parallel diode to block the reverse current. The desired output can be obtained by placing PV cells in series and parallel combinations. The link between the yielded current and yielded voltage is given as [17, 31, 47].

$$I_{pv}=N_{p}I_{ph}-N_{p}I_{rs}\left(\text{exp}\;\left[\frac{q}{AkT_{c}}\left(\frac{V_{dc}}{N_{s}}+\frac{R_{s}I_{pv}}{N_{p}}\right)\right]-1\right)$$ (1)

where *I*_*pv*_ is the PV output current, *I*_*ph*_ is the cell photo current, *N*_*p*_ is the number of solar cell panels connected in parallel, *N*_*s*_ is the number of panels connected in series, *A* is the ideality factor of diode, *k* is the Boltzman constant, *T*_*c*_ is the cell absolute working temperature in ^o^K, *R*_*s*_ is the series resistance of solar cell, *V*_*dc*_ is the PV output voltage and *I*_*rs*_ is the cell reverse saturation current.

$$I_{ph}=\left(I_{sc}+k_{i}\left(T_{c}-T_{ref}\right)\right)\frac{s}{1000}$$ (2)

$$I_{s}=I_{rs}[\frac{T_{c}}{T_{ref}}{]}^{3}\text{exp}\;\left[\frac{{qE}_{g}}{Ak}\left(\frac{1}{T_{ref}}-\frac{1}{T_{c}}\right)\right]$$ (3)

where *k*_*i*_ is cell short circuit current at 25 ^o^C and 1000 W/m^2^, *T*_*ref*_ is cell reference temperature in ^o^K, *s* is the total solar irradiation, W/m^2^, *I*_*sc*_ is cell short circuit current and *I*_*s*_ is the cell saturation current. Eq (4) is the reverse saturation current under its rated temperature and solar irradiance which is given below.

$$I_{rs}=\frac{I_{sc}}{\text{exp}\;\left(\frac{qV_{oc}}{N_{s}kAT_{c}}\right)-1}$$ (4)

where *V*_*oc*_ is the cell open circuit voltage. In this work, 25 panels of PV arrays in series are used where every module contains 18 cells in series. The aforementioned Eqs (1)–(4) show that the PV totally depends on solar irradiance and temperature [17, 31, 47].

The park’s transformation [17] is a technique that transforms, abc components into dq0 components.

From Fig 1 we can obtain the following equations, i.e.

$$\left\{\begin{array}{c} v_{a}=Ri_{a}+L\frac{di_{a}}{dt}+e_{a}\\ v_{b}=Ri_{b}+L\frac{di_{b}}{dt}+e_{b}\\ v_{c}=Ri_{c}+L\frac{di_{c}}{dt}+e_{c} \end{array}\right.$$ (5)

where *R* is the equivalent line resistance of power grid and *L* is equivalent line inductance of a power grid. Also it is assumed for simplicity that *R* = *R*_*a*_ = *R*_*b*_ = *R*_*c*_ and *L* = *L*_1_ = *L*_2_ = *L*_3_. *v*_*a*_, *v*_*b*_, *v*_*c*_ are photovoltaic inverter three phase output voltages and *e*_*a*_, *e*_*b*_, *e*_*c*_ are grid side three phase voltages. *i*_*a*_, *i*_*b*_, *i*_*c*_ are the three phase line currents. The purpose of RL filter is to smooth out the high frequency harmonic components present in the inverter output.

By applying the following Park’s transformation on the system (5), i.e.


abc=23sinωtcosωt12sin.(ωt-(2π3))cos.(ωt-(2π3))12sin.(ωt+(2π3))cos.(ωt+(2π3))12dqo (6)

We obtain the following equations in the rotating d-q frame

$$\left\{\begin{array}{c} v_{d}=e_{d}+{Ri}_{d}+L\frac{{di}_{d}}{dt}+\omega Li_{q}\\ v_{q}=e_{q}+{Ri}_{q}+L\frac{{di}_{q}}{dt}-\omega Li_{d} \end{array}\right.$$ (7)

where *v*_*d*_ and *v*_*q*_ are d-q components of the PV inverter output voltage, *e*_*d*_ and *e*_*q*_ are d- q components of grid voltage, *i*_*d*_ and *i*_*q*_ are d-q components of grid current and *ω* is AC grid frequency.

Eq (8) shows the dependence between the AC output side and the DC input side by ignoring the power losses in the PV inverter.

$$e_{d}i_{d}+e_{q}i_{q}=V_{dc}I_{dc}$$ (8)

where *V*_*dc*_ and *I*_*dc*_ are the PV inverter’s input voltage and current, correspondingly. From Fig 1 we can write

$$C\frac{{dV}_{dc}}{dt}=I_{pv}-I_{dc}=I_{pv}-\frac{e_{d}i_{d}+e_{q}i_{q}}{V_{dc}}$$ (9)

where *C* is the DC link capacitance.

**Fig 2:**
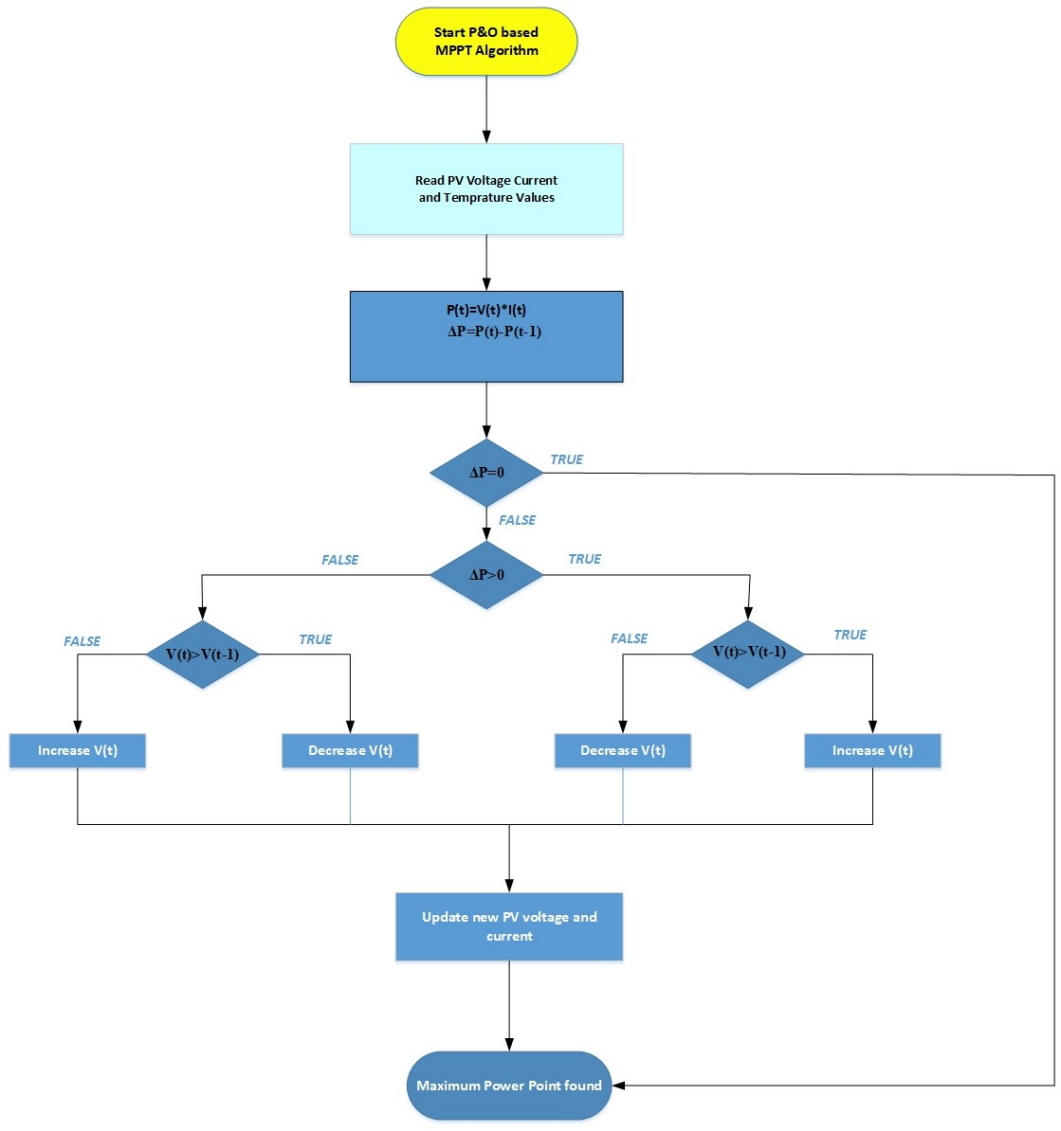
Flowchart of P&O-based MPPT algorithm.

Fig 2 shows the P&O method flow chart [8] based MPPT system. The advantage of P&O and Hill climbing [7] methods is that they are simple. However they have associated disadvantage as mentioned in [8]. Basically P&O method measures solar cell power P and solar cell voltage V. If dP/dV >0 then the actual point is at the left side of MPP, otherwise point is on the right side of MPP. This process lasts until dP/dV = 0, as shown in Fig 2.


$$\left\{\begin{array}{c} \frac{dP}{dV}=0\Rightarrow MPP\\ \frac{dP}{dV}>0\Rightarrow Left\;side\;of\;MPP\\ \frac{dP}{dV}<0\Rightarrow Right\;side\;ofMPP \end{array}\right.$$ (10)

## 3. PBSMC design of three-phase PV inverter for MPPT

### 3.1. Passivity

Eq (11) represents the dynamical nonlinear system.


x˙=fx,uy=h(x,u) (11)

The state vector of the scheme (11) is *x* ∈ *R*^*n*^. *u* ∈ *R*^*m*^ and *y* ∈ *R*^*m*^ corresponds to input and output respectively.

The energy balance [2] can be written as;

$$\underset{\text{Stored}}{\underset{︸}{H\left[x\left(t\right)\right]-H\left[x\left(0\right)\right]}}=\underset{\text{supplied}}{\underset{︸}{\int_{0}^{t}u^{T}\left(s\right)y\left(s\right)ds}}-\underset{\text{dissipate}}{\underset{︸}{d\left(t\right)}}$$ (12)

where *H*(*x*) represents storage function and *d(t)* is a nonnegative function that shows the dissipation effects in practical engineering problems. In case of a continuous differentiable positive semi-definite function *H(x)*, the system (11) is strictly passive such that

$$u^{T}y\geq \frac{\partial H}{\partial x}f\left(x,u\right)+\zeta y^{T}y,\forall (x,u)\in R^{n}{\times R}^{m}$$ (13)

where *ζ* > 0. To acquire the asymptotic stability, Lemma 1 is required as below;

**Lemma 1** [31]. Consider a system (11), the origin of the uncontrolled system, $\dot{x}=f\left(x,0\right)$, is asymptotically stable, when its output is strictly passive and zero-state detectable with a positive definite storage function *H*(*x*). Furthermore, if the storage function *H*(*x*) is unbounded radially, then the origin is globally asymptotically stable.

If system (11) is non-passive and there still exists a positive definite storage function *H*(*x*) and a feedback control law u = β(x) + k*v* such that $\dot{H}\leq vy$, then the feedback system is passive.

As an outcome, the feedback passivation is applied as a primary step in a stabilization design due to the additional output feedback,

v=-ϕ(y) (14)

where *ϕ*(*y*) is a sector-nonlinearity satisfying *yϕ*(*y*) > 0 for *y* ≠ 0 and *ϕ*(0) = 0, which can achieve $\dot{H}\leq y\phi \left(y\right)\leq 0$.

### 3.2. Fractional-order sliding mode control

Fractional-order calculus is based on integration and differentiation in a non-integer order domain, the fundamental operator Dtαa is defined as

Dtαa=dαdtα,α>01,α=0∫at(dτ)-α,α<0 (15)

where *a* and *t* are the lower and upper limits, while *α* ∈ *R* is the operation order.

The Riemann Liouville (R-L) type [32] and Caputo type [31] are the two most prevalent definitions of fractional-order calculus (FOC).

The fractional derivative plus integration using Riemann-Liouville (R-L) of a function *f*(*t*) employing t, is specified by the *α*^*th*^ order R-L fractional derivative and integration.

Dtαaft=1ɼn-αdndtn∫atfτ(t-τ)α-n+1dτ (16)

where ɼ(.) is the gamma function, *n* is the first integer > *α*, e.g. n − 1 ≤ *α* < n.

Moreover, the R-L for a fractional-order integral can be defined as;

Dt-αaft=1ɼα∫at(t-τ)α-1fτdτ (17)

where ɼ(.) is Euler’s Gamma Function defined as, $ɼ\left(\alpha \right)=\int_{0}^{\infty }e^{-t}t^{\alpha -1}dt$.

The Caputo fractional order derivative is given as,

Dtαaft=1ɼn-α∫atfnτ(t-τ)α-n+1dτ (18)

where *n* is the first integer > *α*, e.g., *n* − 1 ≤ *α* < *n*. As before ɼ(.) is the Gamma function.

The Laplace conversion of the Caputo fractional-order derivative (18) is given by

∫0∞Dtα0fte-stdt=sαLf(t)-∑k=0n-1skDtα-k-10ft|t=0 (19)

where $L\left\{.\right\}$ is the Laplace operator. During initial conditions, the fractional order integration with the operation order α can be done by the transfer function $F\left(s\right)=\frac{1}{s^{\alpha }}$ in a frequency domain.

It is important to calculate the numerical solution of fractional systems defined by fractional differential equations. Here, the Oustaloup approximation [31] is adopted to approximate the fractional differentiator for a recursive distribution of poles and zeroes, as given below,

$$s^{\alpha }\approx K\prod_{n=-N}^{N}\frac{1+(\frac{s}{\omega_{z,n}})}{1+(\frac{s}{\omega_{p,n}})},\alpha >0$$ (20)

where 2*N* + 1 is the number of poles and zeros, and *K* is the gain which makes both sides of Eq (16) to have unity gain at 1 rad/s. *ω*_*z*,*n*_ and *ω*_*p*,*n*_ are given as,

$$\omega_{z,n}=\omega_{b}{\left(\frac{\omega_{h}}{\omega_{b}}\right)}^{\left(n+N+(1-\alpha )/2)/(2N+1\right)}$$ (21)

$$\omega_{p,n}=\omega_{b}{\left(\frac{\omega_{h}}{\omega_{b}}\right)}^{\left(n+N+(1+\alpha )/2)/(2N+1\right)}$$ (22)

where *ω*_*b*_ and *ω*_*h*_ represent the lower and upper limits of frequency of approximation, respectively. In general *ω*_*b*_ *ω*_*h*_ *=* 1 *and* $K=\omega_{h}^{\alpha }$.

**Lemma 2** [31]. Consider the following autonomous system.

Dtα0z=Cz,z0=z0 (23)

where *z* ∈ *R*^*n*^ *and C* ∈ *R*^*n×n*^ are asymptotically steady if $|arg\;(eig(C))|>\frac{\alpha \pi }{2}$, in which every element of the state decays towards 0, like *t*^−*α*^. Moreover, system (23) is stable if $|arg\;(eig(C))|\geq \frac{\alpha \pi }{2}$, with those critical eigenvalues satisfying $|arg\;(eig(C))|=\frac{\alpha \pi }{2}$, have geometric multiplicity one.

The fractional sliding surface along with the control law are the two primary characteristics that SMC is based upon. According to the proposed control law, the system must track the sliding surface. The suggested non-integer or fractional sliding mode surfaces are:

$$\begin{array}{c} S_{1}=e_{1}+\lambda {D\;}^{\alpha -1}(sig{\left(e_{1}\right)}^{\gamma })\\ S_{2}=e_{2}+\lambda {D\;}^{\alpha -1}(sig{\left(e_{2}\right)}^{\gamma }) \end{array}$$ (24)

$$e_{1}=i_{q}-i_{q}^{*}$$ (25)

$$e_{2}=V_{dc}-V_{dc}^{*}$$ (26)

where *e*_1_ and *e*_2_ are current and voltage tracking error respectively, *D*^*α*−1^ is the R-L fractional integral of (*α* − 1)^th^ order, *α*, *γ*, and *λ* adds positive parameters along design attributes of (*α* < 1 and *γ* < 1).

The *sig*() function is given as,

$$sig{\left(x\right)}^{\gamma }={\left|x\right|}^{\gamma }sgn(x)$$ (27)

where *sgn*(*x*) function is defined as:

$$sgn\left(x\right)=\left\{\begin{array}{cc} \frac{x}{\left|x\right|}, & if\;x\neq 0\\ 0, & if\;x=0 \end{array}\right.$$ (28)

### 3.3. Pbsmc design

P&O technique in MPPT under different atmospheric conditions is applied to obtain the reference of a DC-link voltage $V_{dc}^{*}$. The reference of q-axis current $i_{q}^{*}$ is evaluated by the PV inverter operator to regulate the unity power factor. The state vectors are given as *x* = (*x*_1_, *x*_2_, *x*_3_)^*T*^ = (*i*_*d*_, *i*_*q*_, *V*_*dc*_)^*T*^, output *y* = (*y*_1_, *y*_2_)^*T*^ = (*i*_*q*_, *V*_*dc*_)^*T*^, and input *u* = (*u*_1_, *u*_2_)^*T*^ = (*v*_*d*_, *v*_*q*_)^*T*^. The tracking error is defined as $e=[e_{1},e_{2}{]}^{T}=[i_{q}-i_{q}^{*},V_{dc}-V_{dc}^{*}{]}^{T}$, where $i_{q}^{*}$ and $V_{dc}^{*}$ are the reference currents and voltages. By differentiating the tracking error e until the control input u appears, we get,

$$\left[\begin{array}{c} \dot{e_{1}}\\ \ddot{e_{2}} \end{array}\right]=\left[\begin{array}{c} f_{1}(x)\\ f_{2}(x) \end{array}\right]+B(x)\left[\begin{array}{c} u_{1}\\ u_{2} \end{array}\right]-\left[\begin{array}{c} \dot{i_{q}^{*}}\\ \ddot{V_{dc}^{*}} \end{array}\right]$$ (29)

where,

$$f_{1}\left(x\right)=-\frac{R}{L}i_{q}+\omega i_{d}-\frac{e_{q}}{L}$$ (30)

$$f_{2}\left(x\right)=\frac{{\dot{I}}_{pv}}{C}-\frac{e_{d}\left(-\frac{R}{L}i_{d}-\omega i_{q}-\frac{e_{d}}{L}\right)+e_{q}\left(-\frac{R}{L}i_{q}+\omega i_{d}-\frac{e_{q}}{L}\right)}{CV_{dc}}-\frac{\left(e_{d}i_{d}+e_{q}i_{q}\right)}{C^{2}V_{dc}^{2}}I_{pv}+\frac{{(e}_{d}i_{d}+e_{q}i_{q}{)}^{2}}{C^{2}V_{dc}^{3}}$$ (31)

$$B\left(x\right)=\left[\begin{array}{cc} 0 & \frac{1}{L}\\ -\frac{e_{d}}{LCV_{dc}} & -\frac{e_{q}}{LCV_{dc}} \end{array}\right]$$ (32)

To make above input–output linearization valid, control gain matrix B(x) must be non-singular during the whole operation range, which needs to satisfy the following equation, i.e.


$$\text{det}\;\left[B\left(x\right)\right]=\frac{e_{d}}{L^{2}CV_{dc}}\neq 0$$ (33)

As component *e*_*d*_ is always different from zero, the above condition can always be satisfied.

A storage function for error tracking dynamics (29) is developed as,

$$H\left(i_{q},V_{dc},I_{dc}\right)=\frac{1}{2}(i_{q}-i_{q}^{*}{)}^{2}+\frac{1}{2}(V_{dc}-V_{dc}^{*}{)}^{2}+\frac{1}{2}(\frac{I_{dc}}{C}-{\dot{V}}_{dc}^{*}{)}^{2}$$ (34)

Here, the storage function *H*(*i*_*q*_, *V*_*dc*_, *I*_*dc*_) consists of sum of heat produced by *i*_*q*_ on a virtual unit AC series-resistor plus heat produced by DC-link voltage *V*_*dc*_ as a virtual unit DC parallel resistor. Whereas the heat generated by DC-link current *I*_*dc*_ is flowing through a virtual unit DC series-resistor.

The first term of storage function (34), e.g. $\frac{1}{2}(i_{q}-i_{q}^{*}{)}^{2}$ tries to regulate power factor; while the later terms, e.g., $\frac{1}{2}(V_{dc}-V_{dc}^{*}{)}^{2}$ and $\frac{1}{2}(\frac{I_{dc}}{C}-{\dot{V}}_{dc}^{*}{)}^{2}$ show energy transformation from the solar energy into electricity. The changes of PV output power are evaluated by the variation of DC-link voltage *V*_*dc*_ and DC-link current *I*_*dc*_ according to relationship (9).

**Remark 1.** MPPT is acquired by adaptable dc-link voltage *V*_*dc*_ (with a degree of 2). One more goal is achieved to control the reactive power, which is controlled by *i*_*q*_ (with a degree of 1). Therefore, storage function and tracking error dynamics only contain dc-link voltage *V*_*dc*_ and reactive component *i*_*q*_ at the same time as the d-axis current *i*_*d*_ is excluded. There are only two inputs *u*_1_ and *u*_2_ but the total order of tracking error dynamics (29) is 3. The two inputs *u*_1_ and *u*_2_ are used to achieve the above-mentioned two goals (2+1 = 3). In this control theory, no more inputs could be adopted for the controlling of the d-axis current *i*_*d*_. Therefore, Eqs (7) to (9) show that after the control of *i*_*q*_ and *V*_*dc*_, the *i*_*d*_ is indirectly controlled.

**Remark 2.** The third term of the storage function H, for example, $\frac{1}{2}(\frac{I_{dc}}{C}-{\dot{V}}_{dc}^{*}{)}^{2}$, is actually $\frac{1}{2}(\dot{V_{dc}}-\dot{V_{dc}^{*}}{)}^{2}$. The connection $C\frac{{dV}_{dc}}{dt}=I_{dc}$ may be used to get this directly. To offer a clearer physical depiction of these two terms, this work does not openly employ their derivative but instead ultimately uses their corresponding connection. The DC-link current *I*_*dc*_ plus DC-link capacitor C of the storage function H, in particular, can be directly measured.

Differentiation of storage function (34) with respect to time is given as,

$$\dot{H}\left(i_{q},V_{dc},I_{dc}\right)=\left(i_{q}-i_{q}^{*}\right)\frac{d}{dt}\left(i_{q}-i_{q}^{*}\right)+\left(V_{dc}-V_{dc}^{*}\right)\frac{d}{dt}\left(V_{dc}-V_{dc}^{*}\right)+(\frac{I_{dc}}{C}-{\dot{V}}_{dc}^{*})\frac{d}{dt}(\frac{I_{dc}}{C}-{\dot{V}}_{dc}^{*})$$ (35)

Substituting $\frac{I_{dc}}{C}=\frac{{dV}_{dc}}{dt}={\dot{V}}_{dc}$ in Eq (35) and simplifying gives

$$\dot{H}\left(i_{q},V_{dc},I_{dc}\right)=\left(i_{q}-i_{q}^{*}\right)\left(-\frac{R}{L}i_{q}+\omega i_{d}-\frac{e_{q}}{L}+\frac{u_{2}}{L}-{\dot{i}}_{q}^{*}\right)+\left(\frac{I_{dc}}{C}-{\dot{V}}_{dc}^{*}\right)\left[\left(V_{dc}-V_{dc}^{*}\right)+\frac{{\dot{I}}_{pv}}{C}-\frac{e_{d}\left(-\frac{R}{L}i_{d}-\omega i_{q}-\frac{e_{d}}{L}\right)+e_{q}\left(-\frac{R}{L}i_{q}+\omega i_{d}-\frac{e_{q}}{L}\right)}{CV_{dc}}-\frac{\left(e_{d}i_{d}+e_{q}i_{q}\right)}{C^{2}V_{dc}^{2}}I_{pv}+\frac{{(e}_{d}i_{d}+e_{q}i_{q}{)}^{2}}{C^{2}V_{dc}^{3}}-\frac{e_{d}}{LCV_{dc}}u_{1}-\frac{e_{q}}{LCV_{dc}}u_{2}-{\ddot{V}}_{dc}^{*}\right]$$ (36)

Design of PBSMC for system (29) is given as

$$u_{1}=-\frac{LCV_{dc}}{e_{d}}\left[{\ddot{V}}_{dc}^{*}-V_{dc}+V_{dc}^{*}+\frac{e_{q}}{LCV_{dc}}u_{2}-\frac{{\dot{I}}_{pv}}{C}+\frac{e_{d}\left(-\frac{R}{L}i_{d}-\omega i_{q}-\frac{e_{d}}{L}\right)+e_{q}\left(-\frac{R}{L}i_{q}+\omega i_{d}-\frac{e_{q}}{L}\right)}{CV_{dc}}+\frac{\left(e_{d}i_{d}+e_{q}i_{q}\right)}{CV_{dc}^{2}}\dot{V_{dc}^{*}}+\nu_{1}\right]$$ (37)

$$u_{2}=L\dot{i_{q}^{*}}-{\omega Li}_{d}+Ri_{q}^{*}+e_{q}+\nu_{2}$$ (38)

where *ν*_1_ and *ν*_2_ denote additional inputs. Substituting values of *u*_1_ and *u*_2_ in Eq (36), together with the DC-link relationship (9) gives,

$$\dot{H}\left(i_{q},V_{dc},I_{dc}\right)=-\frac{1}{CR_{dc}}({\dot{V}}_{dc}-{\dot{V}}_{dc}^{*}{)}^{2}-\frac{R}{L}(i_{q}-i_{q}^{*}{)}^{2}+\left({\dot{V}}_{dc}-{\dot{V}}_{dc}^{*}\right)v_{1}+\frac{i_{q}-i_{q}^{*}}{L}v_{2}$$ (39)

where $R_{dc}=\frac{{V_{dc}}^{2}}{e_{d}i_{d}+e_{q}i_{q}}$

Differentiating Eq (24) we get

$$\begin{array}{c} \dot{S_{1}}=\dot{i_{q}}-\dot{i_{q}^{*}}+\lambda {D\;}^{\alpha }(sig{\left(e_{1}\right)}^{\gamma })\\ \dot{S_{2}}=\dot{V_{dc}}-\dot{V_{dc}^{*}}+\lambda {D\;}^{\alpha }(sig{\left(e_{2}\right)}^{\gamma }) \end{array}$$ (40)

Eqs (7) and (9) can be rewritten as

$$v_{q}=m_{q}V_{dc}=e_{q}+{Ri}_{q}+L\frac{{di}_{q}}{dt}-\omega Li_{d}$$ (41)

$$C\frac{{dV}_{dc}}{dt}=I_{pv}-\frac{e_{d}i_{d}+e_{q}i_{q}}{V_{dc}}=I_{pv}-\frac{{(e}_{d}i_{d}+e_{q}i_{q})m_{d}}{v_{d}}$$ (42)

where we have made use of the following equations

$$v_{q}=m_{q}V_{dc}$$ (43)

$$v_{d}=m_{d}V_{dc}$$ (44)

where *m*_*d*_(*t*) and *m*_*q*_(*t*) are d and q components of modulation signals for Sinusoidal Pulse Width Modulation (SPWM) that are modulating *V*_*dc*_. Assuming $i_{q}^{*}$ and $V_{dc}^{*}$ are constants or slowly varying. Substituting Eqs (41) and (42) in Eq (40), the modified equations can be written as

$$\dot{S_{1}}=f_{1}\left(.\right)+V_{dc}m_{q}+\rho_{1}+\lambda {D\;}^{\alpha }(sig{\left(e_{1}\right)}^{\gamma })$$ (45)

$$\dot{S_{2}}=f_{2}\left(.\right)+xm_{d}+\rho_{2}+\lambda {D\;}^{\alpha }(sig{\left(e_{2}\right)}^{\gamma })$$ (46)

where *D*^*α*^ is the R-L operator, *ρ*_1_ and *ρ*_2_ are model uncertainity terms [33] and

$$f_{1}\left(.\right)=-\frac{e_{q}}{L}-\frac{Ri_{q}}{L}+\omega i_{d}$$ (47)

$$f_{2}\left(.\right)=I_{pv}$$ (48)

$$x=-\frac{{(e}_{d}i_{d}+e_{q}i_{q})}{v_{d}}$$ (49)

Based on Eqs (45) and (46), the proposed control law certifies the reference error (current) tracking convergence and produces modulating signals *m*_*d*_ and *m*_*q*_ for SPWM which can be defined as,

$$m_{q}=\frac{-\left[f_{1}\left(.\right)+\lambda {D\;}^{\alpha }\;\left(sig{\left(e_{1}\right)}^{\gamma }\right)+k_{q}sgn(S_{1})\right]}{V_{dc}}$$ (50)

$$m_{d}=\frac{-\left[f_{2}\left(.\right)+\lambda {D\;}^{\alpha }\;\left(sig{\left(e_{2}\right)}^{\gamma }\right)+k_{d}sgn(S_{2})\right]}{x}$$ (51)

where terms *k*_*d*_ and *k*_*q*_ signifies FOSMC sliding gains.

## 4. Stability analysis

The definition of Lyapunov function [33] for the stability of proposed FOSMC is described as

$$V\left(t\right)=\frac{1}{2}(S_{1}^{2}+S_{2}^{2})$$ (52)

By taking time derivative of Eq (52) we get

$$\dot{V}\left(t\right)=S_{1}\dot{S_{1}}+S_{2}\dot{S_{2}}$$ (53)

Substituting Eqs (45) and (46) in Eq (53) we get

$${{\dot{V}\left(t\right)=S}_{1}(f}_{1}\left(.\right)+V_{dc}m_{q}+\rho_{1}+\lambda {D\;}^{\alpha }(sig{\left(e_{1}\right)}^{\gamma }))+S_{2}(f_{2}\left(.\right)+xm_{d}+\rho_{2}+\lambda {D\;}^{\alpha }(sig{\left(e_{2}\right)}^{\gamma }))$$ (54)

Substituting Eqs (50) and (51) in Eq (54) we get

$${\dot{V}\left(t\right)=S}_{1}\left(\rho_{1}-k_{q}sgn\left(S_{1}\right)\right)+S_{2}(\rho_{2}-k_{d}sgn\left(S_{2}\right))$$ (55)

Considering $sgn\left(S_{1}\right)=\frac{\left|S_{1}\right|}{S_{1}}$ and $sgn\left(S_{2}\right)=\frac{\left|S_{2}\right|}{S_{2}}$

$$\dot{V}\left(t\right)={(S}_{1}\rho_{1}-k_{q}\left|S_{1}\right|)+{(S}_{2}\rho_{2}-k_{d}\left|S_{2}\right|)$$ (56)

Now assigning (*k*_*q*_ = |*ρ*_1_| + *ξ*_*q*_) and (*k*_*d*_ = |*ρ*_2_| + *ξ*_*d*_), where *ξ*_*q*_ and *ξ*_*d*_ are positive parameters. Eq (56) yields

$$\dot{V}\left(t\right)\leq -\xi_{q}\left|S_{1}\right|-\xi_{d}\left|S_{2}\right|\leq -min\;(\xi_{q},\xi_{d})(\left|S_{1}\right|+\left|S_{2}\right|)$$ (57)

where min(*ξ*_*q*_, *ξ*_*d*_) represents minimum of (*ξ*_*q*_, *ξ*_*d*_). Eq (57) verifies the stability criteria, finite time convergence of proposed FOSMC sliding surface *S*_1_ and *S*_2_. Therefore, $\dot{V}\left(t\right)$ is negative, and the proposed FOSMC system is asymptotic stable [33].

The additional inputs are then designed as

$$v_{1}=f_{1}\left(.\right)+V_{dc}m_{q}+\rho_{1}+\lambda {D\;}^{\alpha }(sig{\left(e_{1}\right)}^{\gamma })$$ (58)

$$v_{2}=f_{2}\left(.\right)+xm_{d}+\rho_{2}+\lambda {D\;}^{\alpha }(sig{\left(e_{2}\right)}^{\gamma })$$ (59)

The control laws are provided using Eqs (58) and (59) to slide the scheme on a sliding surface and ensure rapid and robust tracking error convergence.

**Remark 3.** To avoid over-current, the classical linear PI and PID control technique is used as an inner current-loop to manage the inverter’s three-phase current. The suggested PBSMC system, on the other hand, is a nonlinear approach that lacks an inner current loop in its control law and cannot tolerate over-current. As a result, the over-current prevention devices [22] will be turned on to prevent the over-current from increasing.

**Fig 3:**
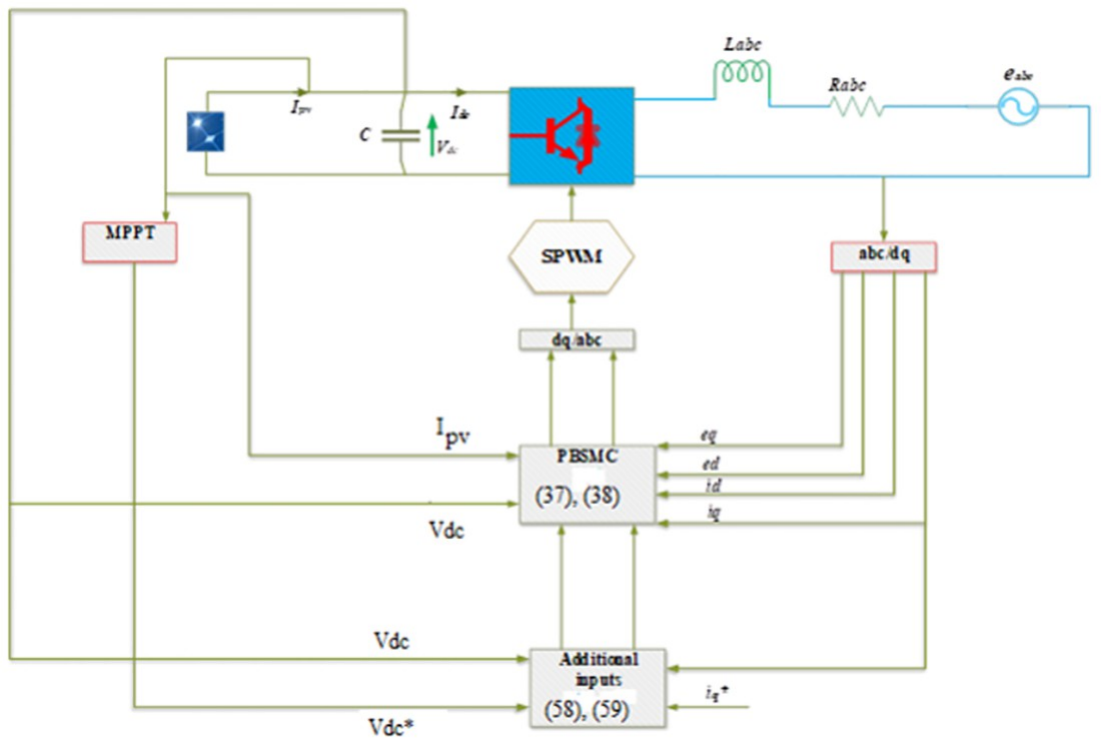
The overall PBSMC structure of the grid-connected PV inverter for MPPT.

Fig 3 shows the overall structure of PBSMC. Three-phase current and voltage components are converted into d-q components, which are controlled by the controller which work as a passivity-based controller. An additional input of Eqs (58) and (59) are added to the PBC as additional inputs which performed as SMC. Then d-q components are transformed into abc components and given to SPWM. SPWM produces pulses at a switching frequency of 10 kHz.

## 5. Results and analysis

Three cases, i.e. solar irradiance changes, solar irradiance and temperature changes, and power grid voltage drop are adopted. The performance indices of each case are thoroughly analyzed.

### 5.1. Case studies

Three representative control systems, i.e, FOPID [32], PBFOPID control [32], and PBC control [5, 31] along with the proposed PBSMC system are simulated and analyzed under the subsequent three cases, i.e. (a) Solar irradiance change in the presence of constant temperature; (b) Solar irradiance change plus temperature variation; and (c) Power grid voltage drop; Furthermore, because the control inputs may exceed the PV inverter’s acceptable capacity at some operating points, their values are restricted in the range [-1.3, 1.3]. Tables 1 and 2 include a list of PV system parameters from [48] as well as PBSMC parameters established by trial and error.

**Table 1:** PV system parameters.

**Typical peak power**	213.15 W	**Series resistance**	0.0848 Ω
**Voltage at peak power**	29 V	**Grid voltage**	400 V
**Current at peak power**	7.35 A	**Grid frequency (*f*)**	50 Hz
**Short-circuit current**	7.84 A	**Grid inductance (*L*)**	0.003395 H
**Open-circuit voltage**	36.3 V	**Grid resistance Line (*R*)**	1e-6 Ω
**Temperature coefficient of I**_**sc**_	0.12 A/C	**DC bus capacitance (*C*)**	2955 µF

**Table 2:** Parameters of proposed PBSMC and PI controller.

**q-axis current control**	γ = 0.9	λ = 1500	K_q_ = 477	K_p_ = 5	K_i_ = 5000
**DC-link voltage**	γ = 0.9	λ = 1500	K_d_ = 477	K_p_ = 5	K_i_ = 5000

Furthermore, the first solar irradiance and temperature, as well as the q-axis current *i*_*q*_ = 0, are set to their rated values, e.g., 1 kW/m^2^ and 25°C. PV output power P is 95920 W, DC link voltage *V*_*dc*_ is 730 V, and PV output current *I*_*pv*_ is 132.3 A, correspondingly, under such standard conditions.

The voltage mentioned in Table 1 is three phase line to line rms voltage, whereas that mentioned in the figure is single phase peak voltage, i.e.

Singlephasepeakvoltage=(400/sqrt(3))xsqrt(2)=323.4V.

Where ‘sqrt’ stands for the square root.

### 5.2 Solar irradiance change

The step changes in solar irradiance are investigated as shown in Table 3;

**Table 3:** Three consecutive steps changes in solar irradiance.

Solar irradiance	Time
1 kW/m^2^	0 sec
0.5 kW/m^2^	0.2 sec
0.8 kW/m^2^	0.7 sec
1 kW/m^2^	1.2 sec

**Fig 4:**
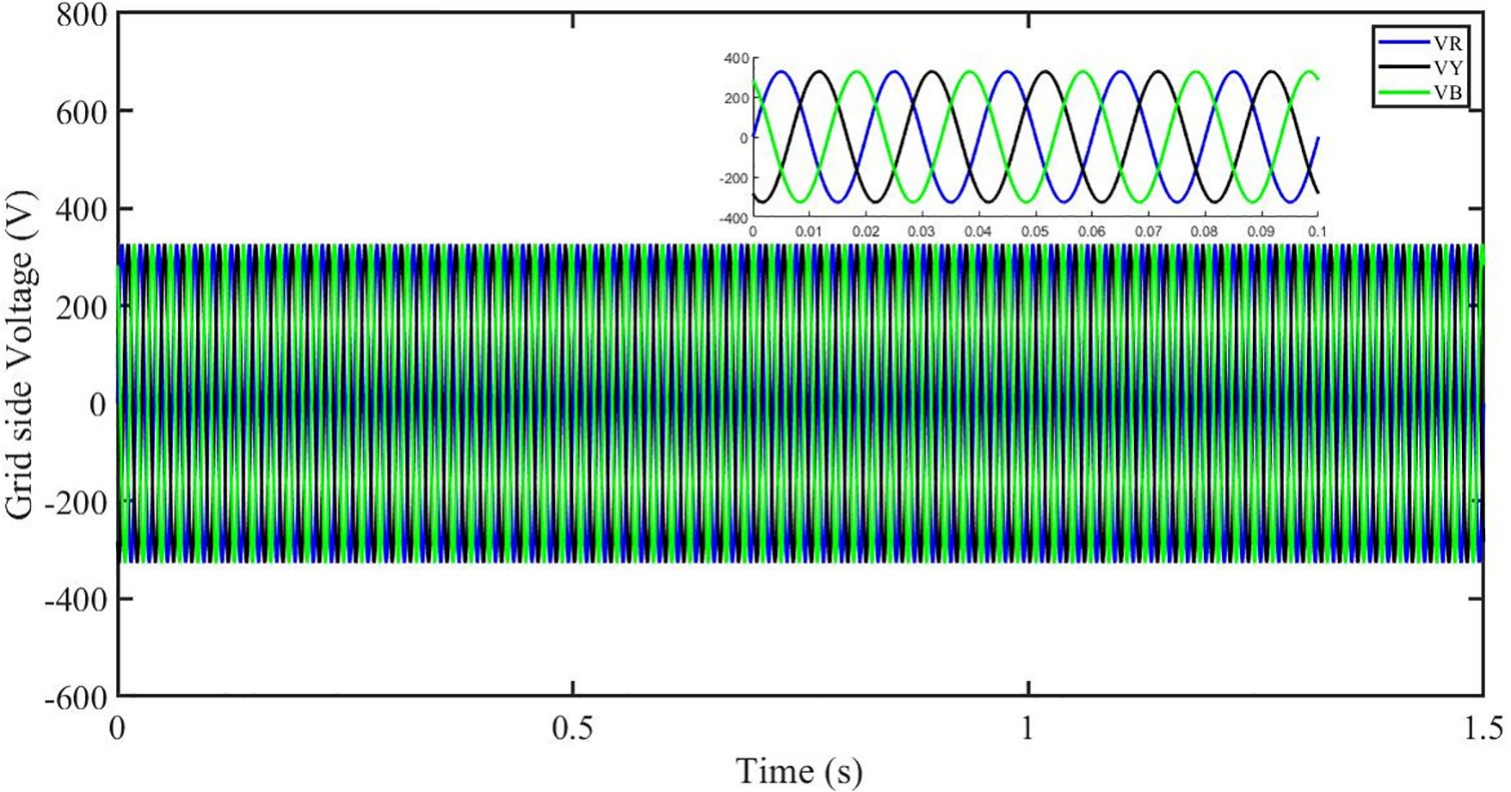
Three-phase voltages at the grid side under solar irradiance change.

**Fig 5:**
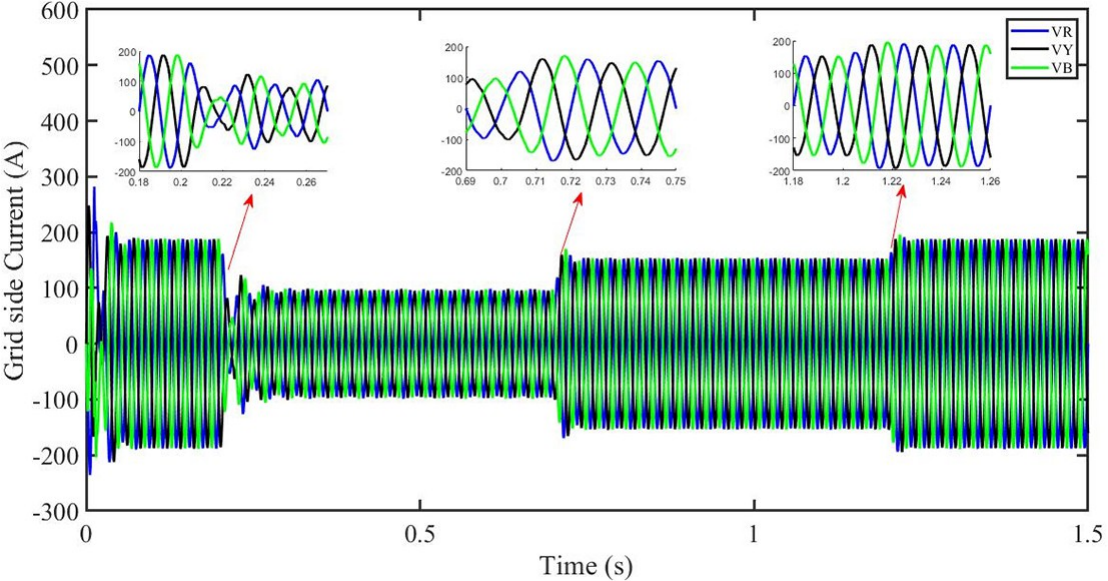
Three-phase currents at the grid side under solar irradiance change.

This section aims to study the robust performance of the proposed control strategy under solar irradiance change conditions. It is supposed that the system initially operates under solar irradiance change conditions. Fig 4 shows that the three-phase voltages are sinusoidal but the three-phase currents are dropped to 100 amperes at 0.2 sec because of dropping of solar irradiance to 0.5 kW/m^2^, then rising to 150 amperes as the irradiance changed to 0.8 kW/m^2^ at 0.7 sec and recovered at 1.2 sec with the irradiance of 1 kW/m^2^ as shown in Fig 5. This causes the output inverter current non-sinusoidal but the PBSMC is capable of removing oscillations from the output voltage, thus the voltage waveform looks quite sinusoidal. Hence, the proposed controller performance is good under irradiance change conditions. The THD of the output voltage is within limits as per IEEE standards.

**Fig 6:**
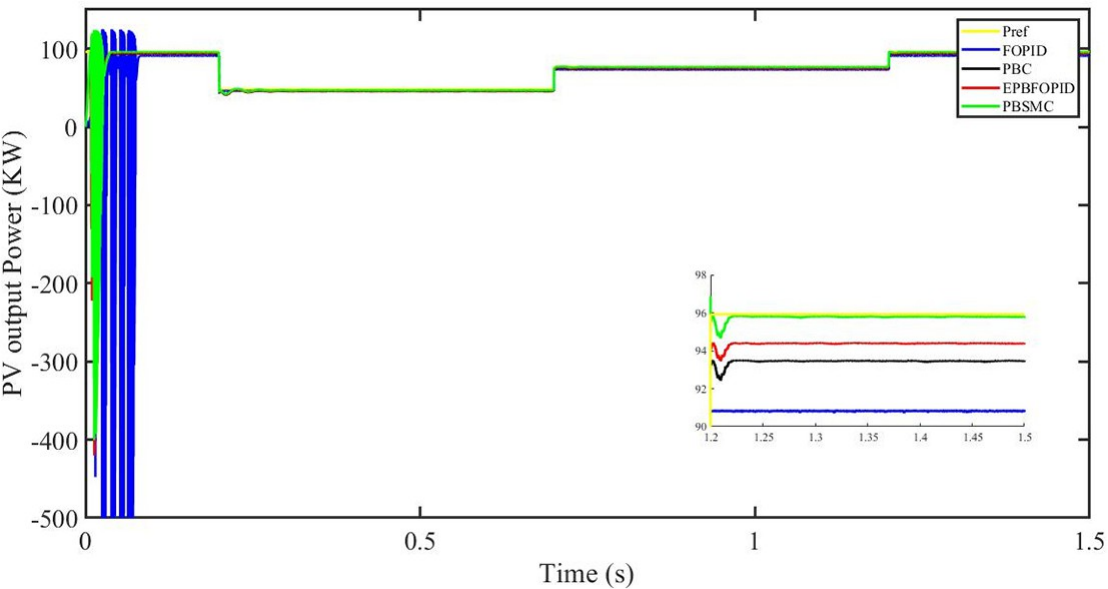
PV output power.

**Fig 7:**
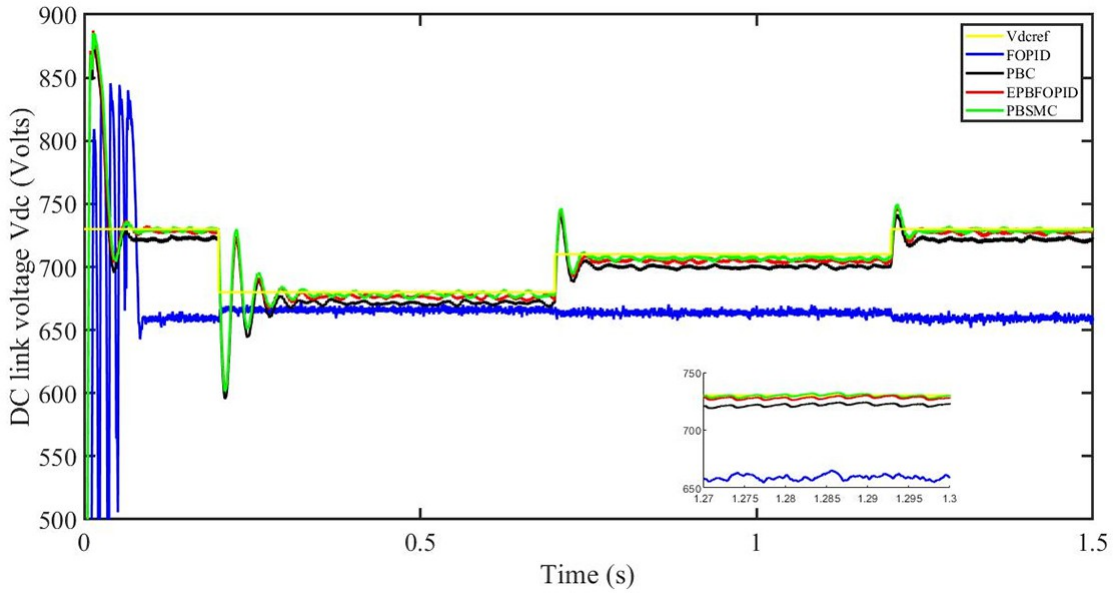
DC link voltage *V*_*dc*_.

**Fig 8:**
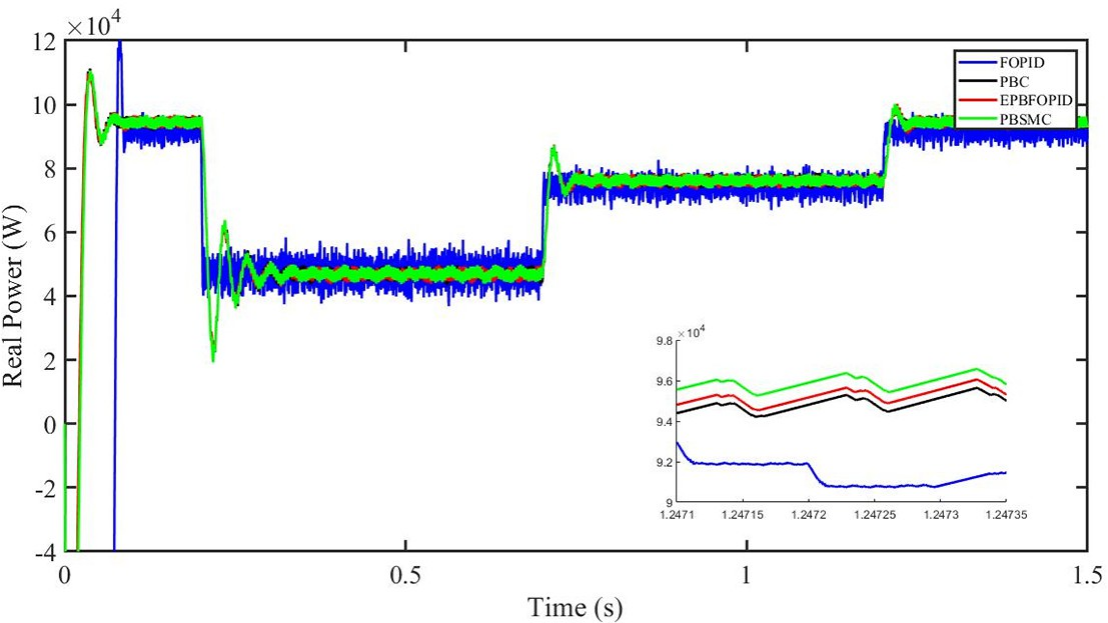
Real power (W).

**Fig 9:**
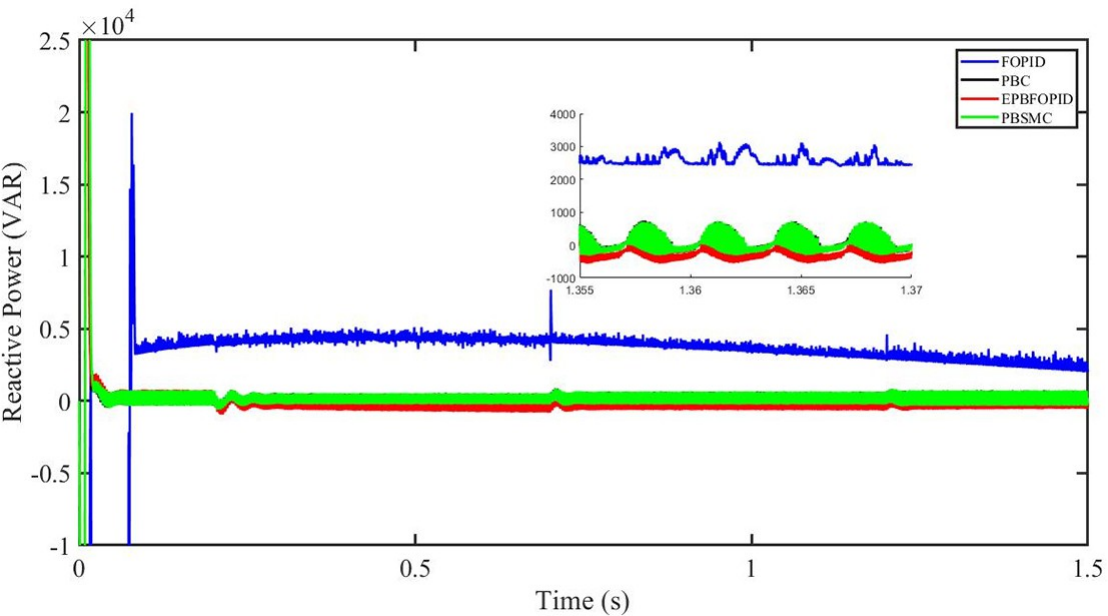
Reactive power (VAR).

**Fig 10:**
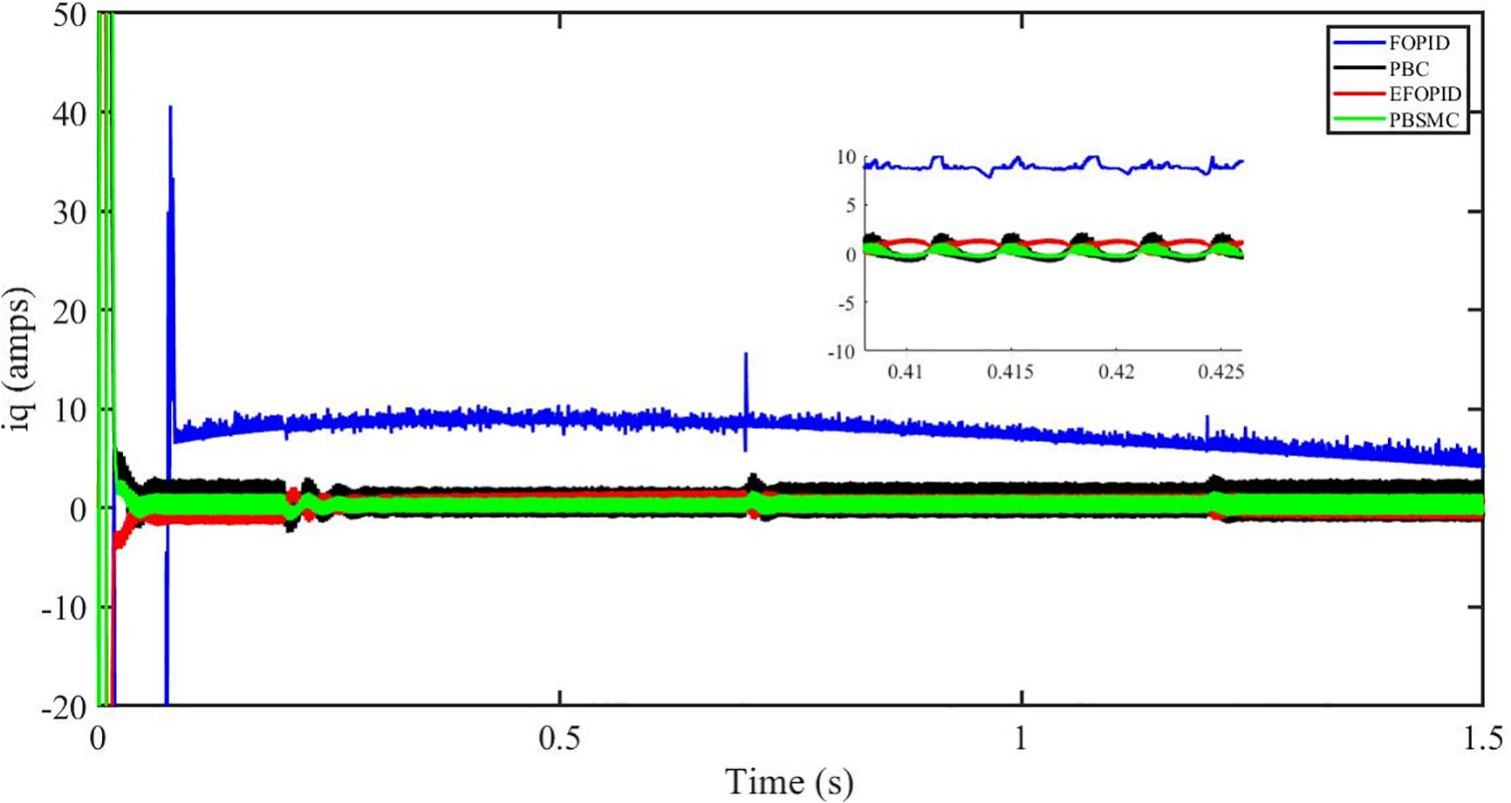
iq (amps).

**Fig 11:**
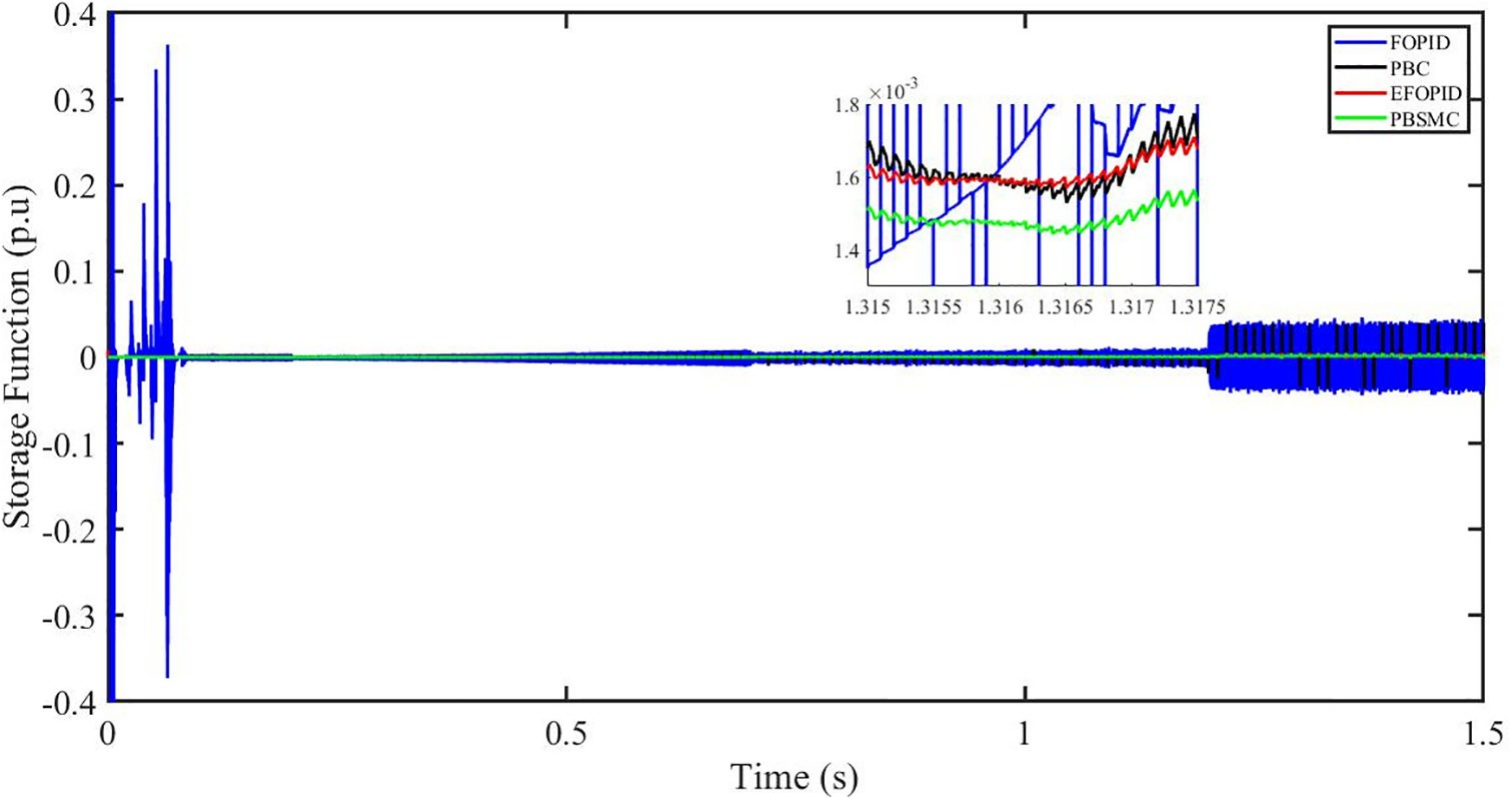
Storage function (p.u).

Figs 6 to 11 show how the responses of PV system alter when the solar irradiation changes. The amalgamation of passivity and fractional-order sliding mode methods allows PBSMC to provide the fastest tracking rate. Finally, the real-time difference of the storage function *H*(*i*_*q*_, *V*_*dc*_, *I*_*dc*_) shows that PBSMC has the quickest tracking speed (steepest slope) and the smallest tracking error (lowest peak value). Figs 6 and 7 show the fast tracking of the reference by the proposed controller. Fig 9 shows that reactive power injected into the grid is zero, i.e. real power (Fig 8) is injected into the grid at unity power factor, by the proposed controller, however FOPID method could not achieve nonzero reactive power initially but it is tending towards zero. Similarly, the proposed controller rendered the quadrature current, *i*_*q*_ almost zero, as shown in Fig 10. On the other hand FOPID method has nonzero *i*_*q*_, however it is tending towards zero.

**Fig 12:**
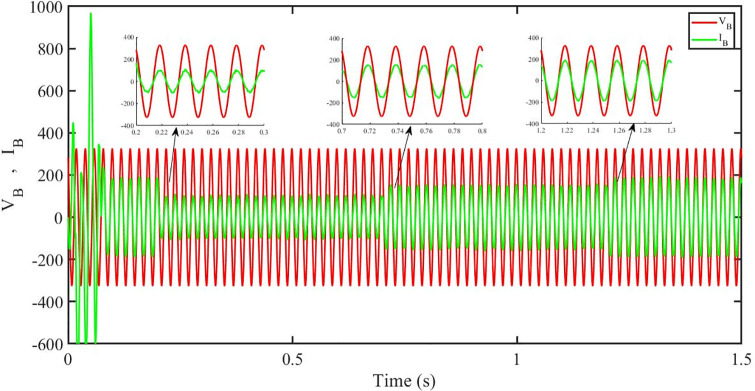
Grid side voltage and current corresponding to blue phase for FOPID.

Fig 12 shows grid side voltage and current corresponding to blue phase for FOPID under solar irradiance change.

**Fig 13:**
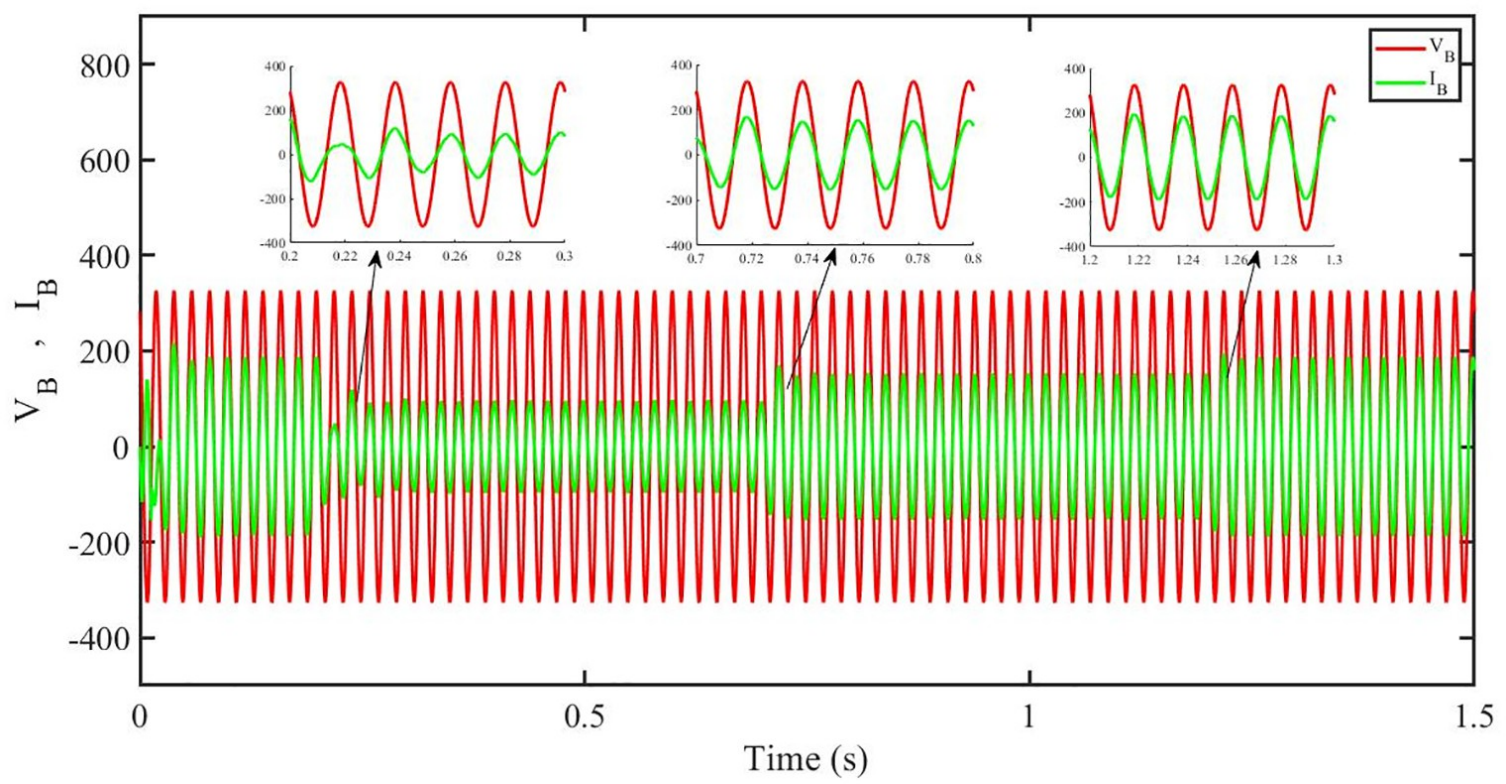
Grid side voltage and current corresponding to blue phase for PBC.

Fig 13 shows grid side voltage and current corresponding to blue phase for PBC under solar irradiance change. As can be seen the voltage and current are in phase due to unity power factor.

**Fig 14:**
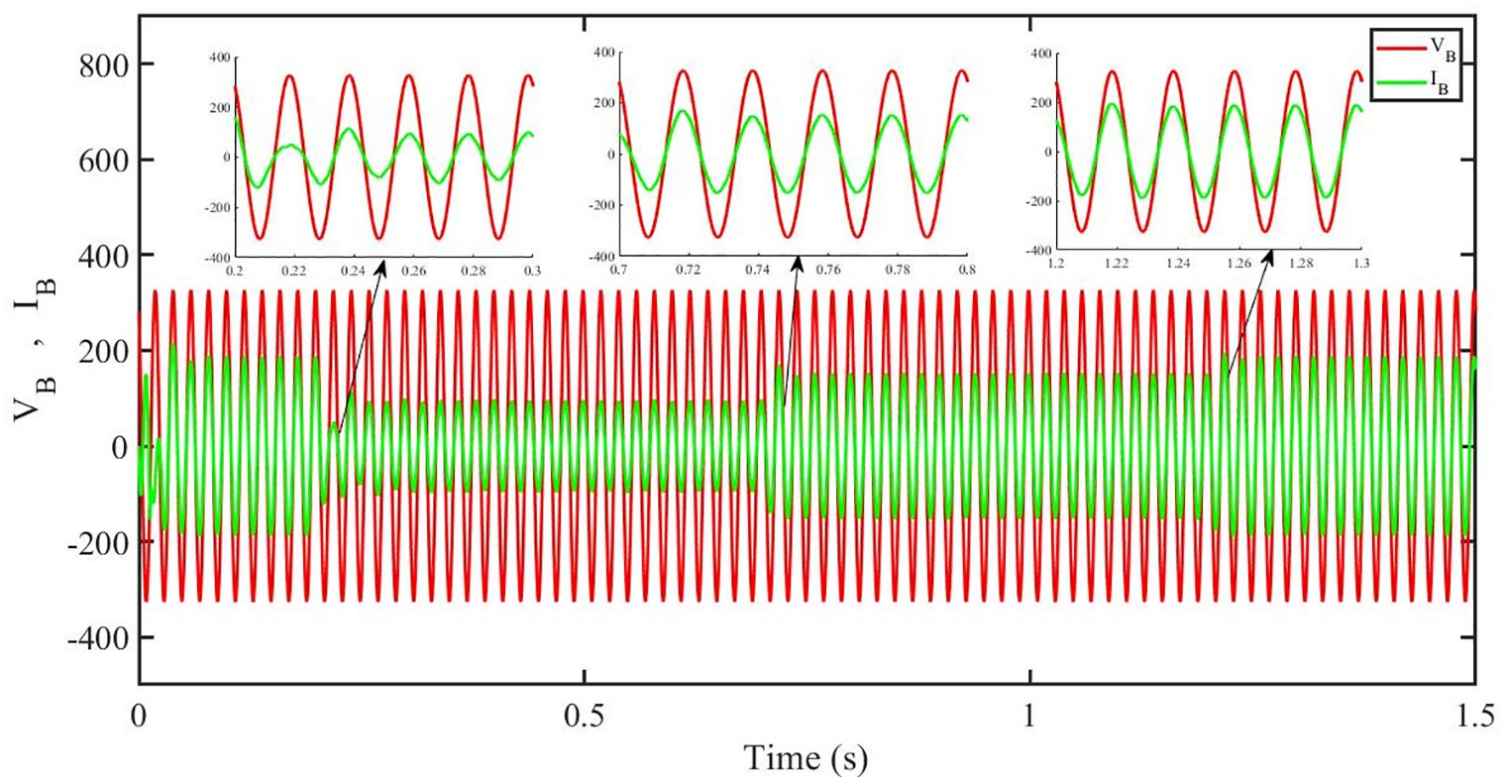
Grid side voltage and current corresponding to blue phase for PBFOPID.

Fig 14 shows grid side voltage and current corresponding to blue phase for PBFOPID under solar irradiance change. As can be seen the voltage and current are in phase due to unity power factor.

**Fig 15:**
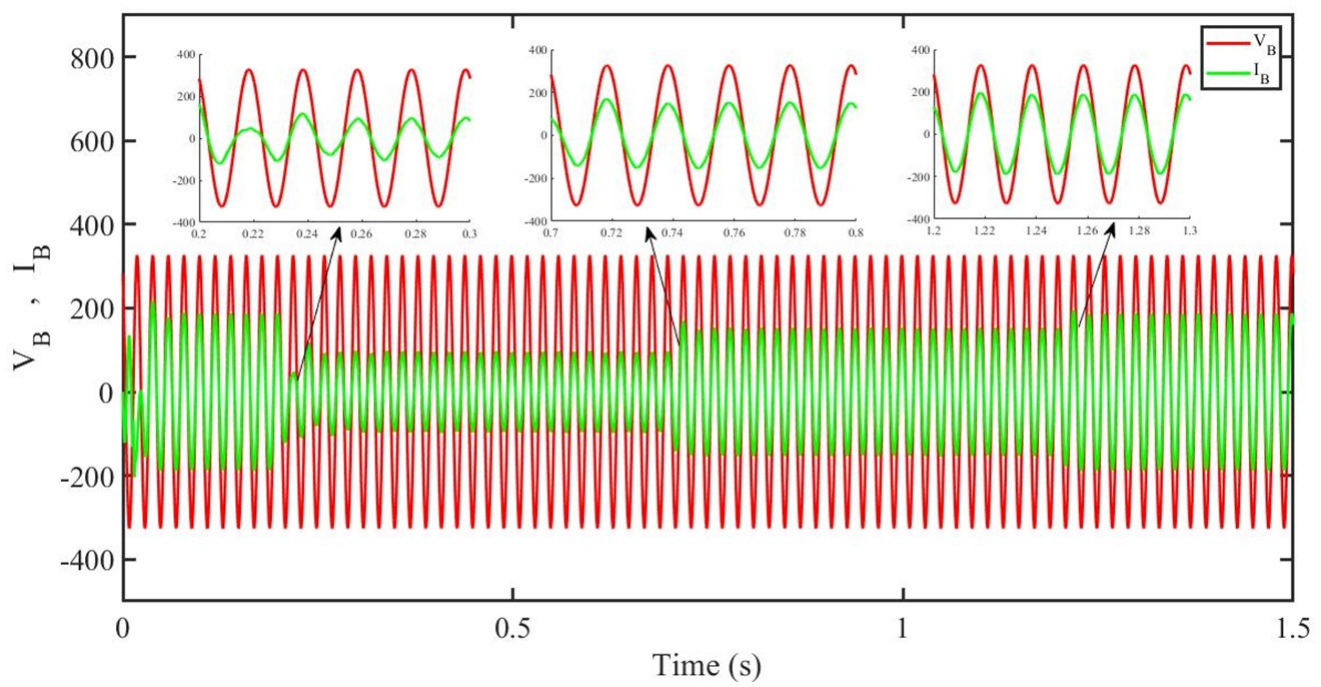
Grid side voltage and current corresponding to blue phase for PBSMC.

Fig 15 shows grid side voltage and current corresponding to blue phase for PBSMC under solar irradiance change. As can be seen the voltage and current are in phase due to quadrature current being rendered zero.

#### 5.2.1 Performance Indices of PV output power under solar irradiance change

**Fig 16:**
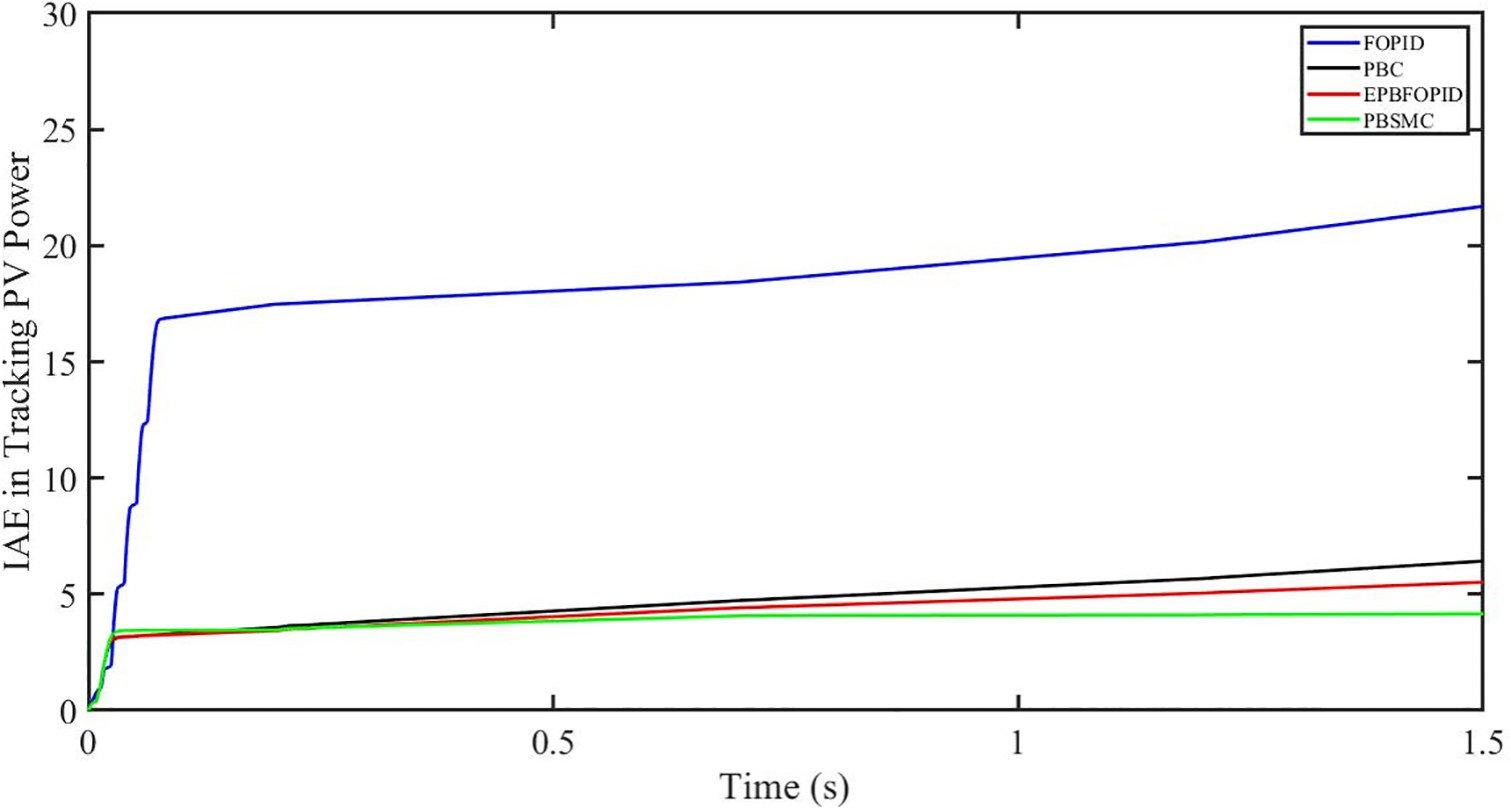
IAE in Tracking PV power.

**Fig 17:**
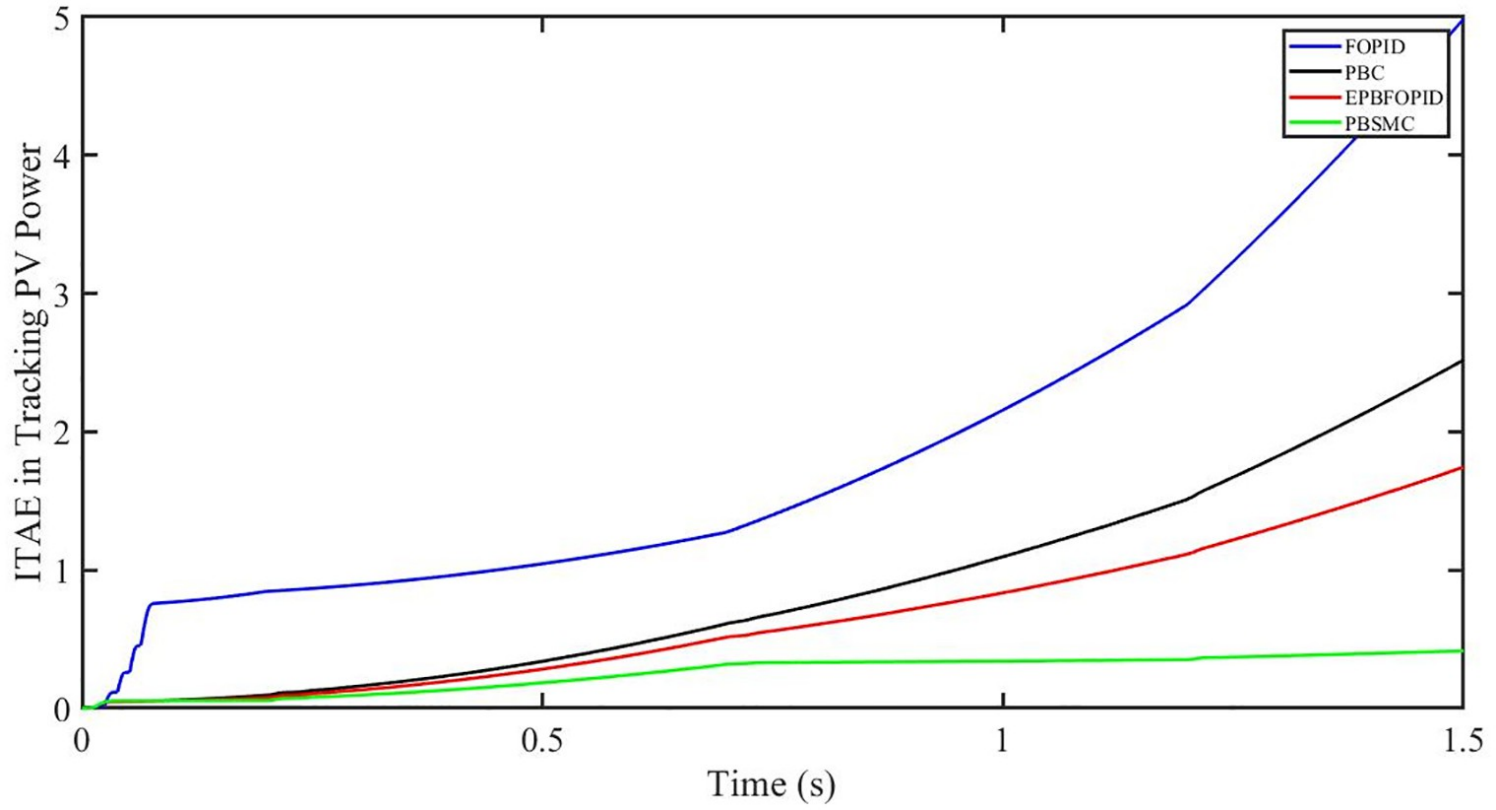
ITAE in Tracing PV power.

**Fig 18:**
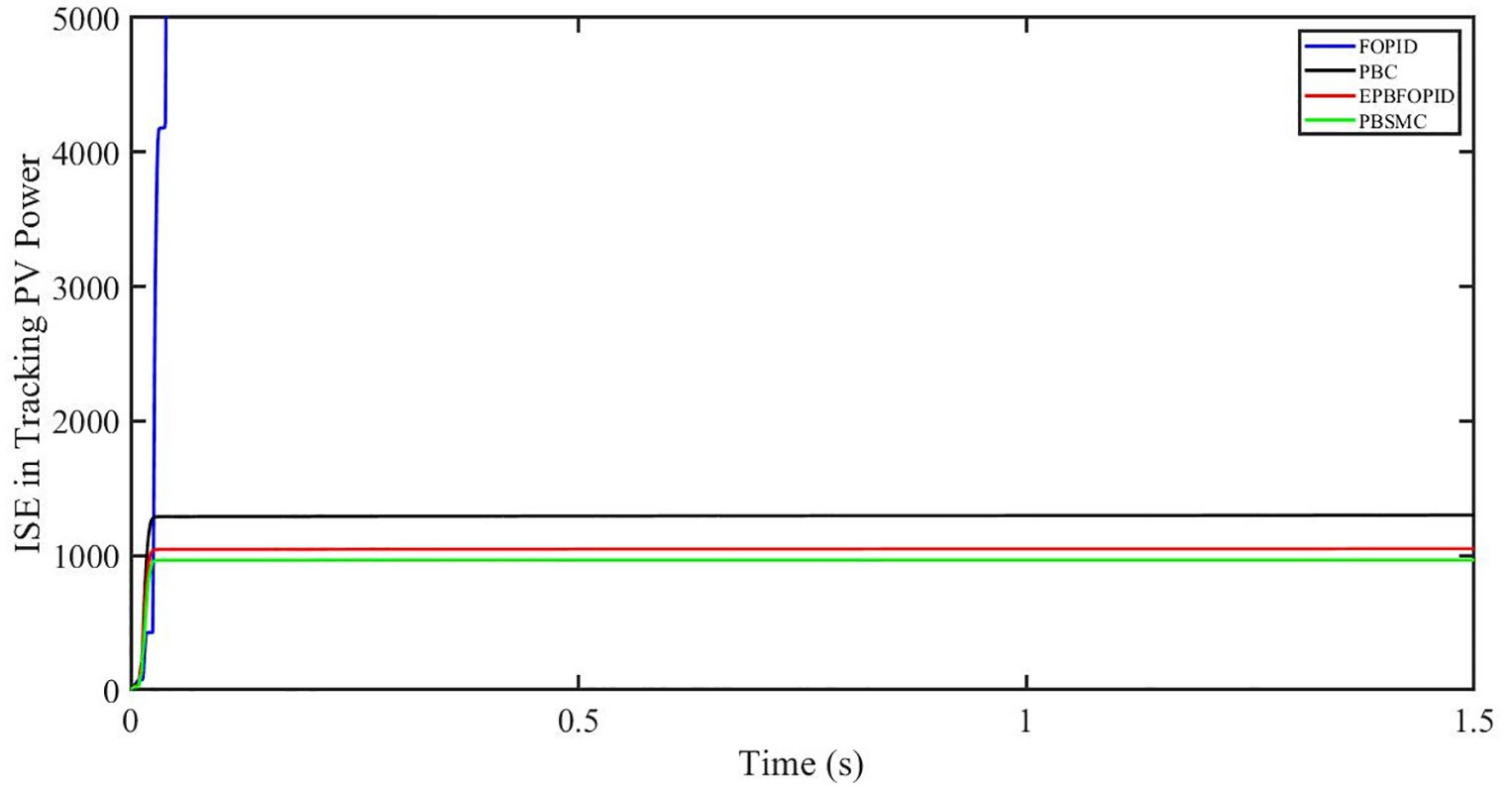
ISE in tracking PV power.

The performance indices of the four controllers, i.e. Integral absolute error (IAE), Integral time absolute error (ITAE), and Integral square error (ISE) [31] are listed below. To investigate the whole operating range of three cases, the simulation time T = 1.5 s was used. PBSMC has the lowest IAE, ITAE, and ISE indices for PV output power with solar irradiance variation, as shown in Figs 16 to 18 respectively. As a result, it performs better than the other three controllers.

The performance metrics are defined as follows,

$$IAE(t)=\int_{0}^{t}\left|Є\right|dt$$ (60)

$$ITAE(t)=\int_{0}^{t}t\left|Є\right|dt$$ (61)

$$ISE(t)=\int_{0}^{t}{Є}^{2}dt$$ (62)

where Є is the error. In case of tracking PV power, Є is defined as difference between reference PV power and actual PV power, whereas in case of tracking V_dc_*, Є is defined as difference between reference V_dc_ and actual V_dc_*.

#### 5.2.2 Performances Indices of dc-link voltage V_dc_ under solar irradiance change

**Fig 19:**
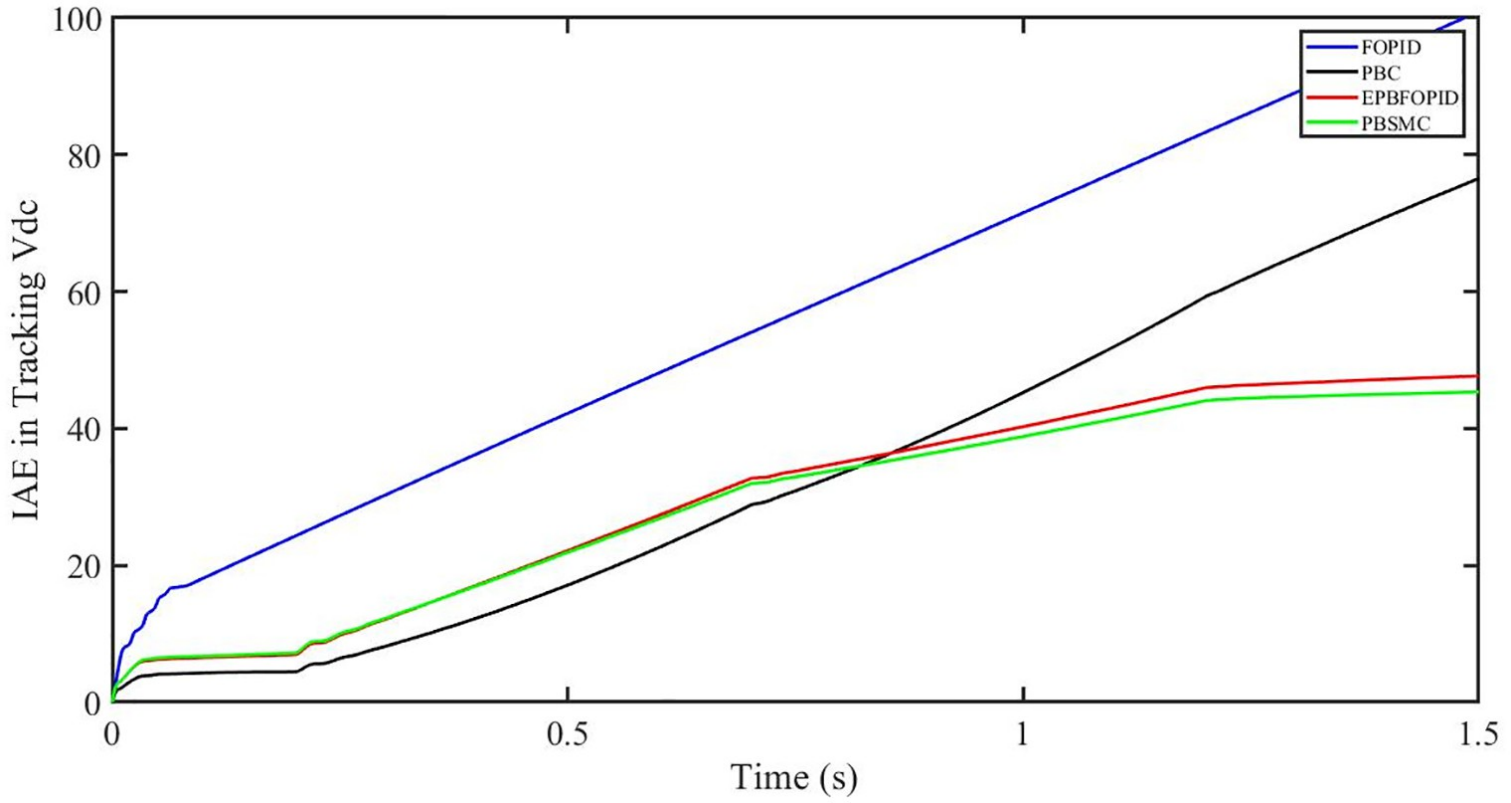
IAE in Tracking *V*_*dc*_.

**Fig 20:**
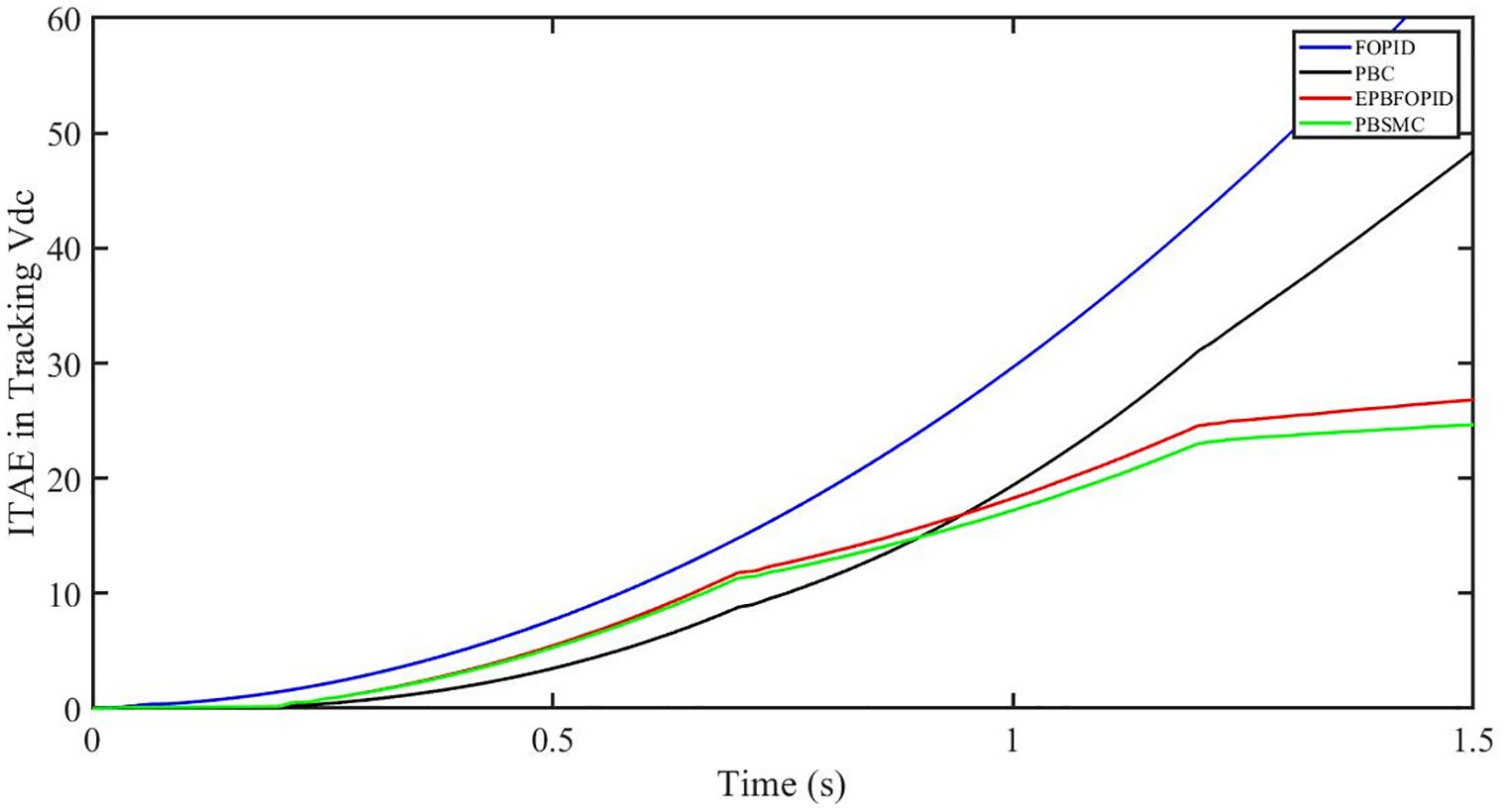
ITAE in Tracking *V*_*dc*_.

**Fig 21:**
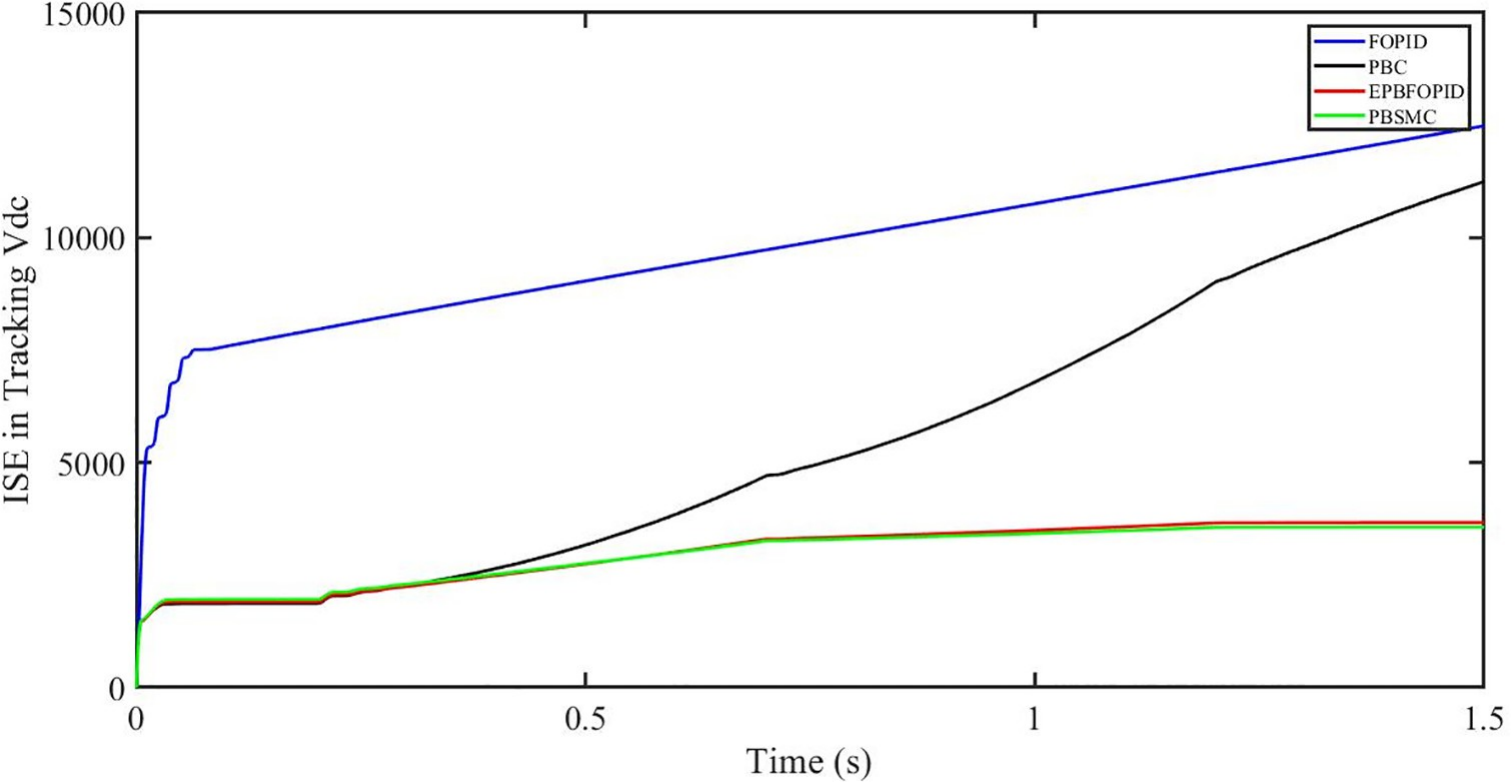
ISE in tracking *V*_*dc*_.

The performance indices of the four controllers, i.e. IAE, ITAE, and ISE indices are listed below. To investigate the whole operating range of three cases, the simulation time T = 1.5 s was used. PBSMC has the lowest IAE, ITAE, and ISE indices for dc-link voltage V_dc_ during solar irradiation variation, as shown in Figs 19 to 21. As a result, it performs better than the other three controllers.

### 5.3 Temperature variation and solar irradiance change

Three-step changes in ambient temperature are explored.

**Fig 22:**
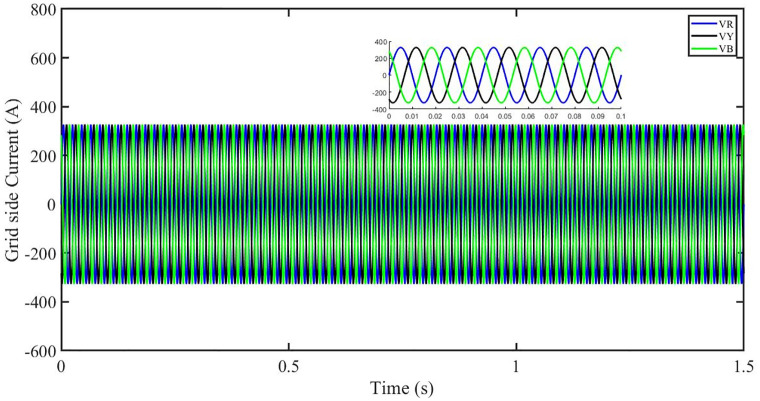
Three-phase voltages at the grid side under solar irradiance change.

**Fig 23:**
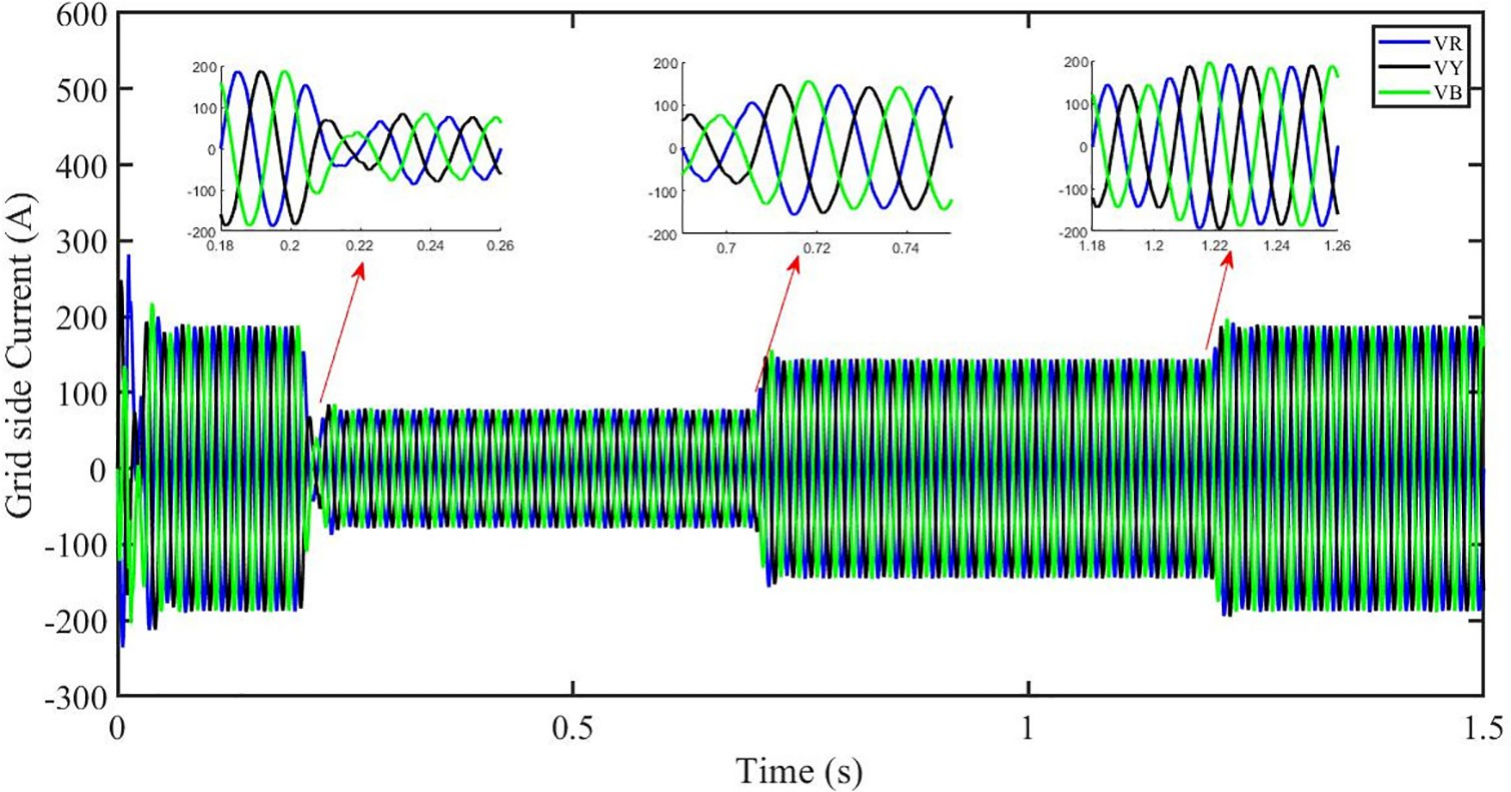
Three-phase currents at the grid side under solar irradiance change.

It is supposed that the system operates under a solar irradiance change and temperature variation condition. Fig 22 shows that the three-phase voltages are sinusoidal but the three-phase currents are dropped to 90 amperes at 0.2 sec because of dropping of solar irradiance to 0.5 kw/m^2^ and increase of temperature to 33 °C. Then the currents rise to 130 amperes as the irradiance changed to 0.8 kw/m^2^ and temperature is increased to 40 °C at 0.7 sec and finally the current is recovered at 1.2 sec with the irradiance of 1 kw/m^2^ and temperature dropped to 25 °C as shown in Fig 23. We can see that the increase in temperature caused drop in three-phase current. This causes the output inverter current non-sinusoidal but the PBSMC is capable of removing oscillations from the output voltage, thus the voltage waveform looks quite sinusoidal. Hence, the proposed controller performance is quite effective under irradiance change and temperature variation conditions. Also, the Total Harmonic Distortion (THD) of the output voltage is within limits as per IEEE standards.

Meanwhile, the solar irradiance is adjusted as described in Table 4, with PV power and grid power reducing to 50 KW at t = 0.2 seconds and then being restored at t = 1.2 seconds. However, during the lowering point, reactive power and i_q_ current are at their maximum value. At t = 0.2 seconds, the dc-link voltage drops and then restores at t = 1.2 seconds. Temperature changes cause a lot of harmonics, uncertainties, and a reduction in dc-link voltage, yet PBSMC immediately recovered the parameters.

**Table 4:** Three consecutive steps changes in temperature.

Temperature	Time
25 °C	0 sec
33 °C	0.2 sec
40 °C	0.7 sec
25 °C	1.2 sec

**Fig 24:**
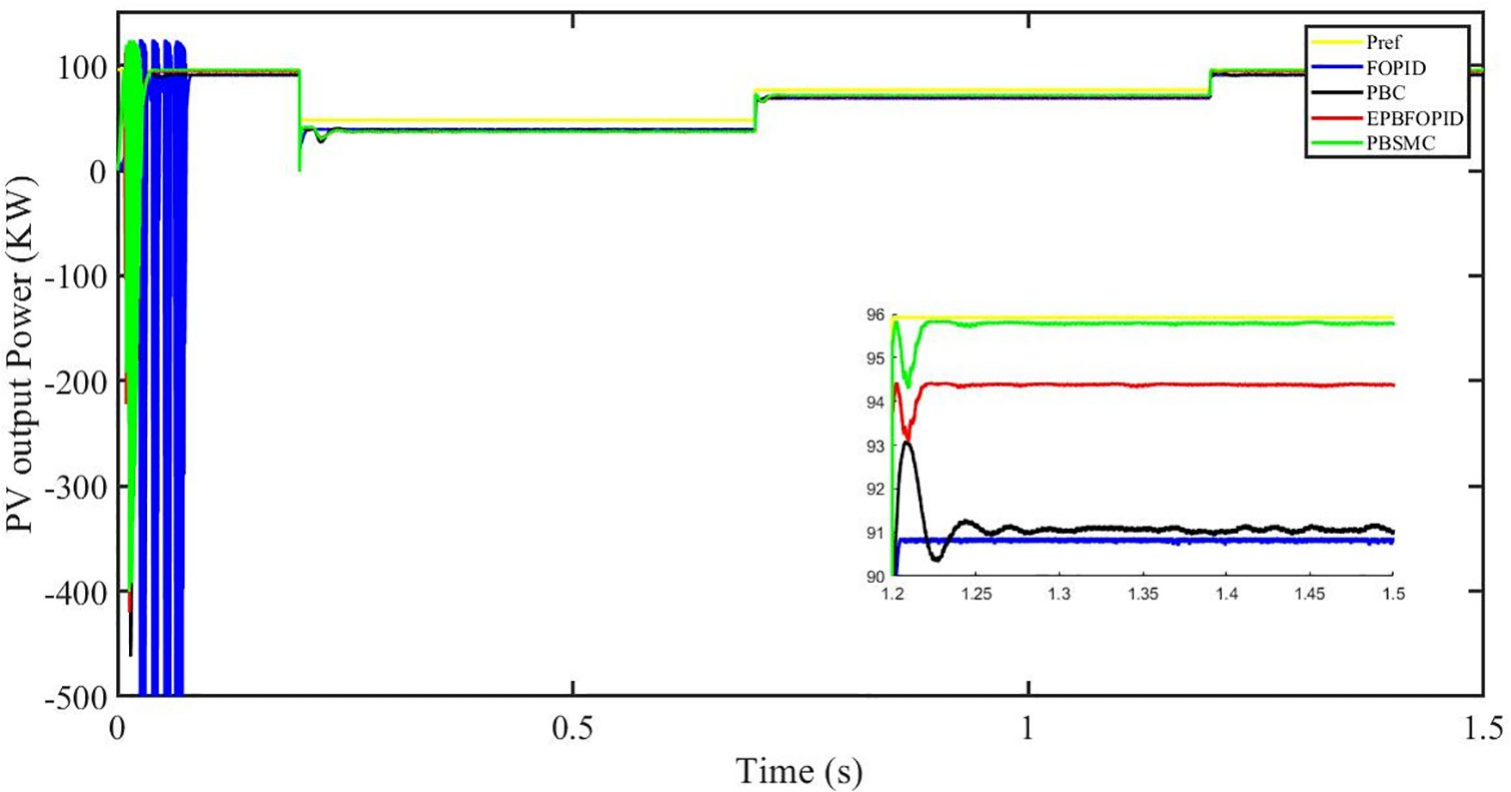
PV output power (kW).

**Fig 25:**
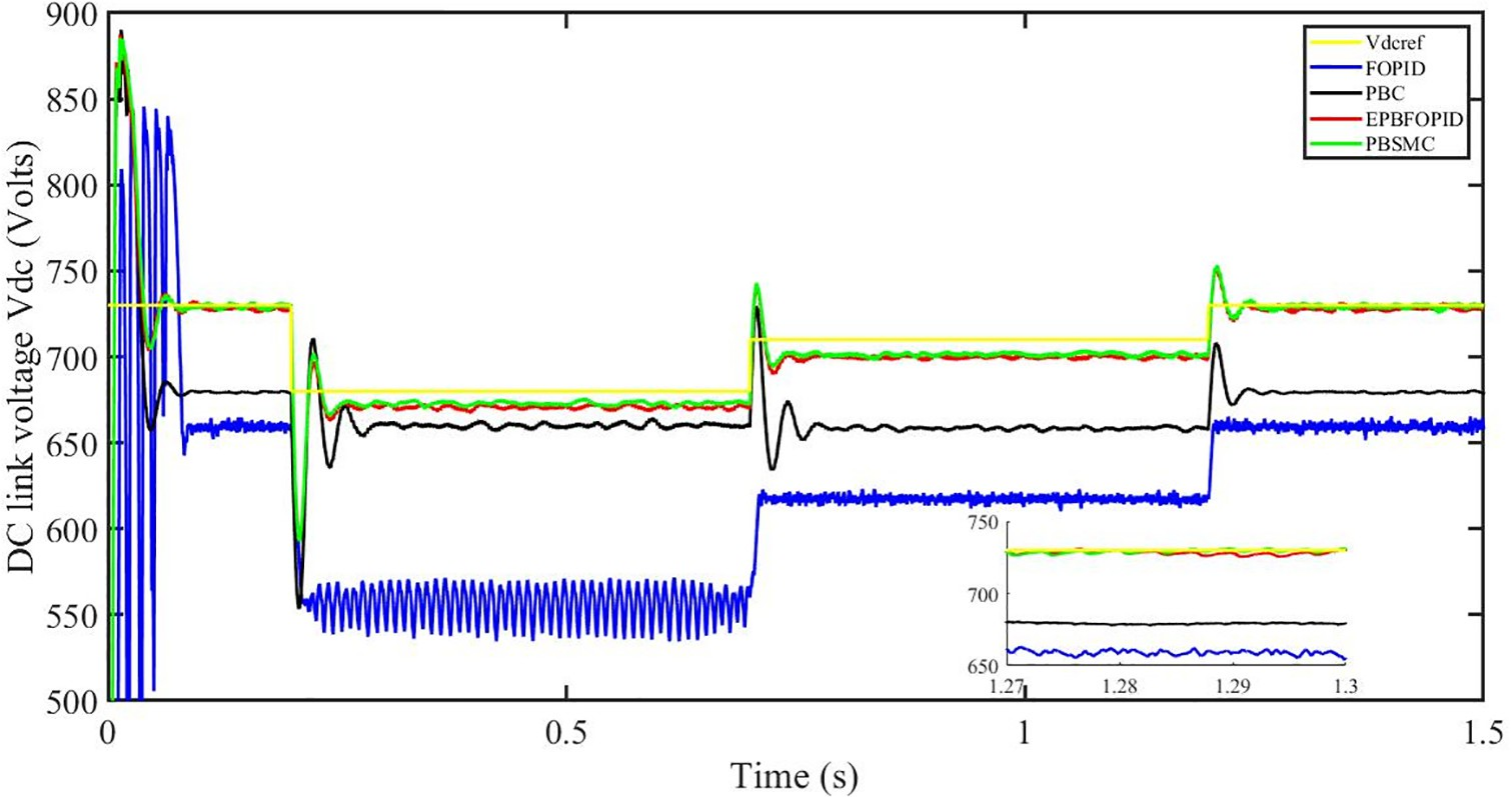
DC link voltage *V*_*dc*_.

**Fig 26:**
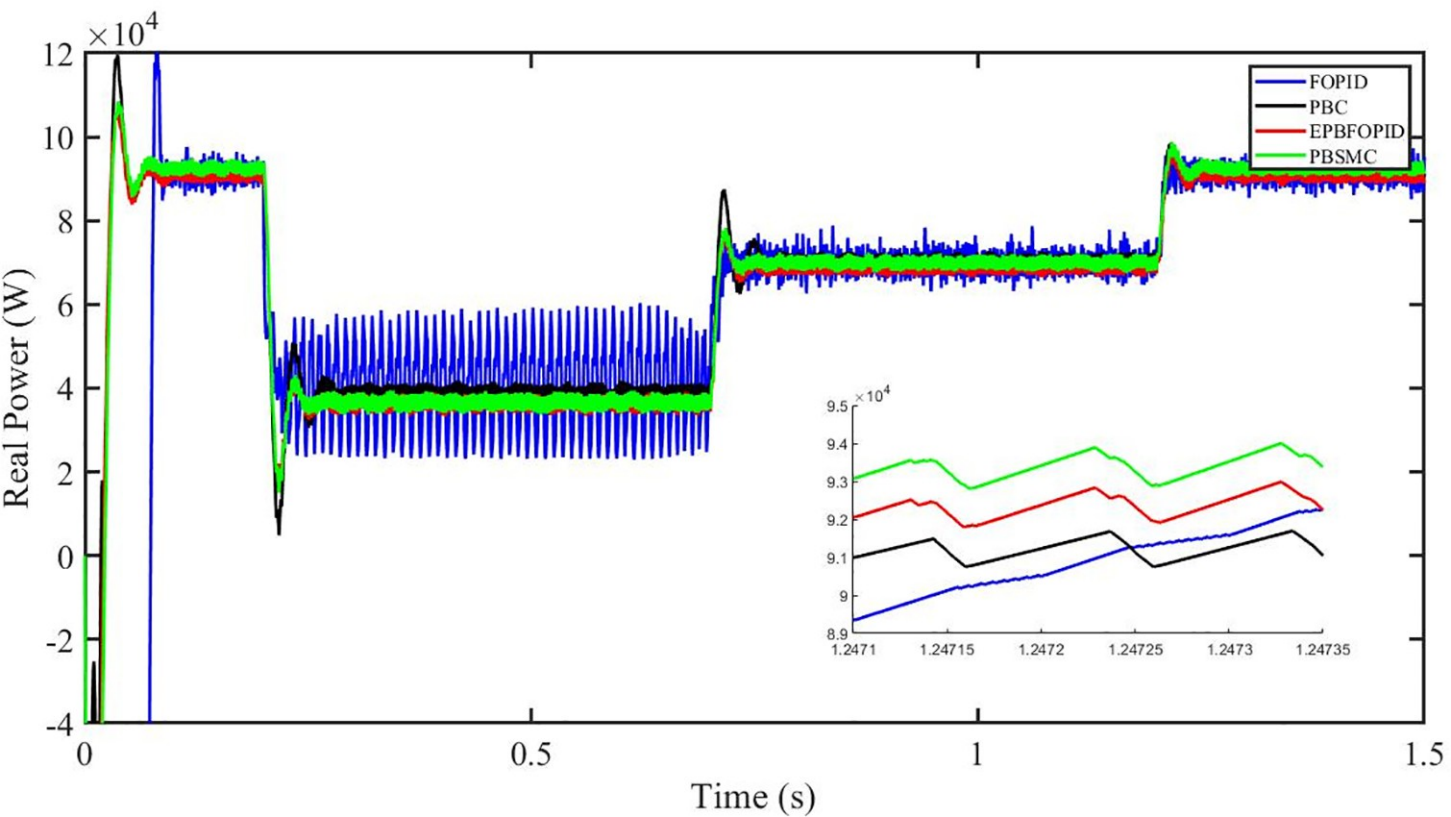
Real power (W).

**Fig 27:**
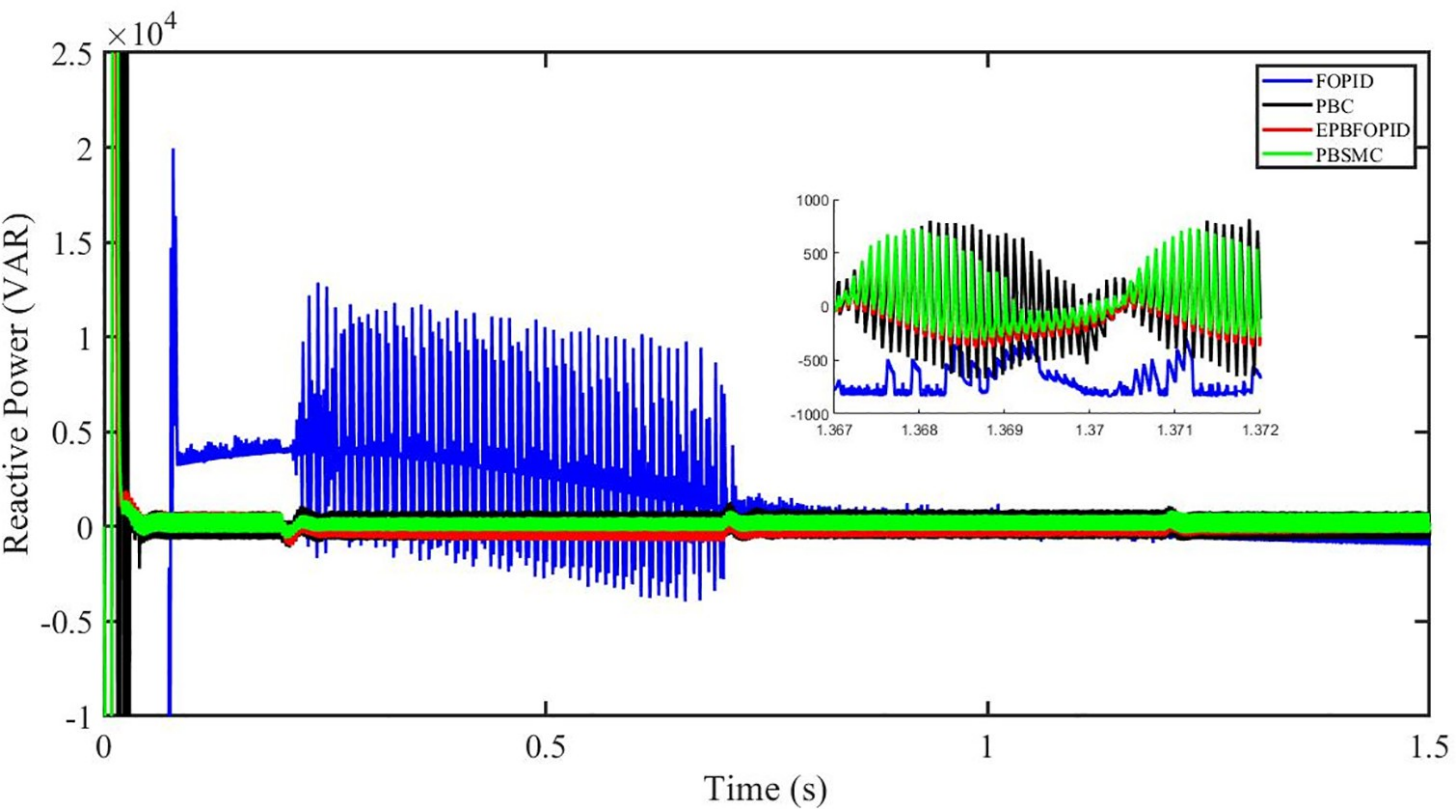
Reactive power (VAR).

**Fig 28:**
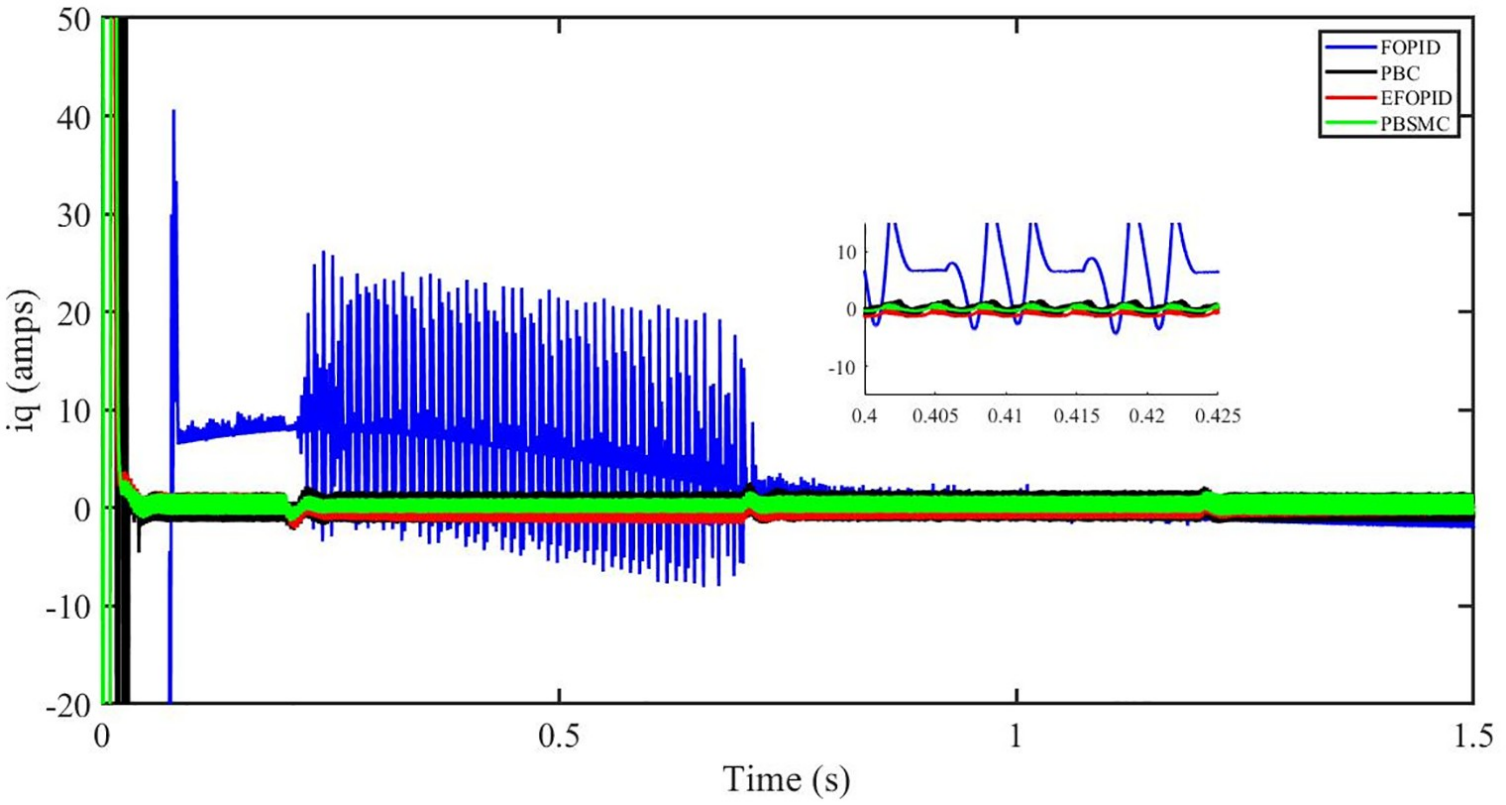
iq (amps).

**Fig 29:**
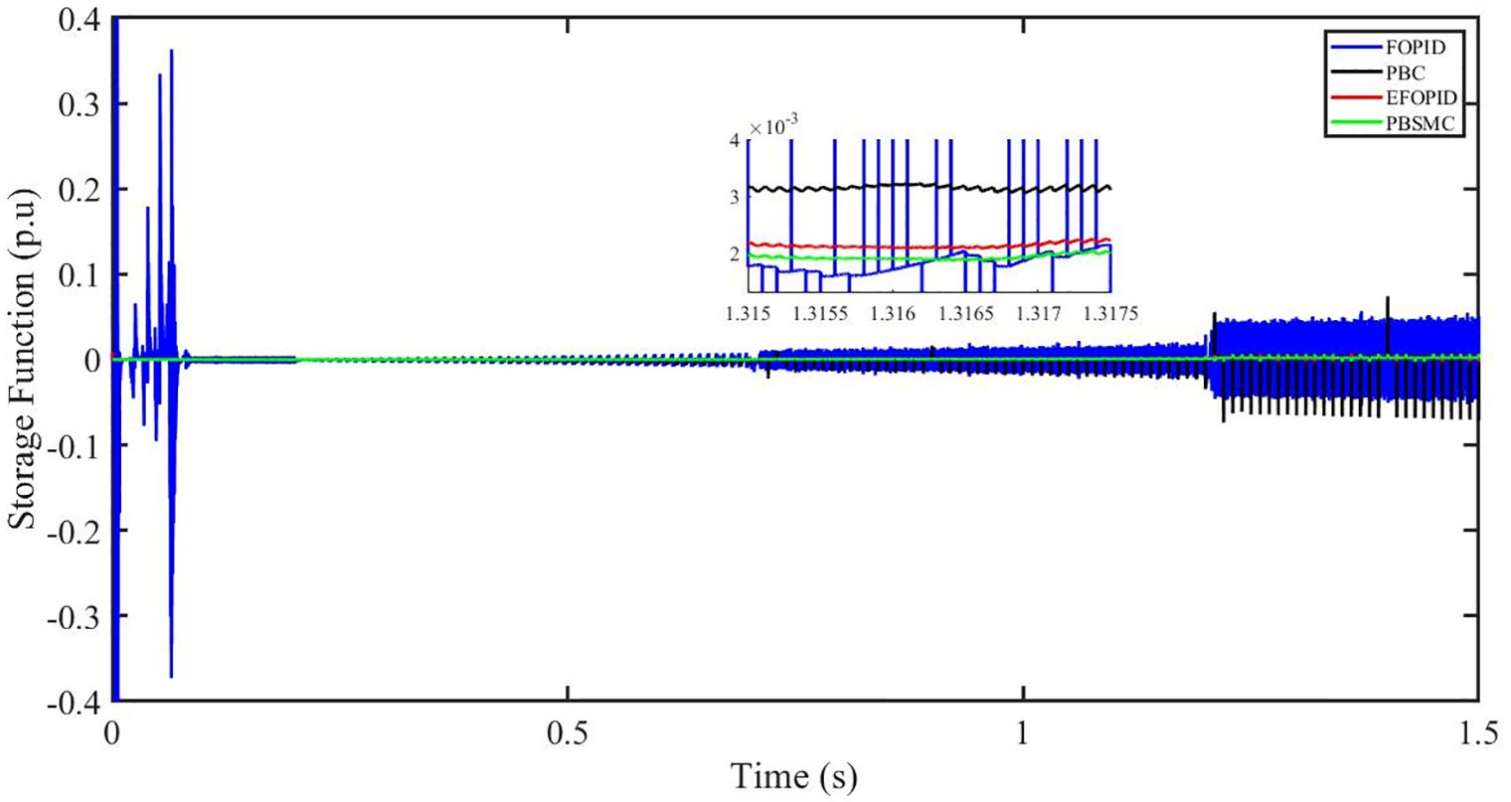
Storage function (p.u).

The outcome of the PV system responses is shown in Figs 24 to 29, which reveals that PBSMC has the best control performance amongst the four controllers since it has the maximum tracking speed with no overshoot. Figs 24 and 25 shows the fast tracking of the reference by the proposed controller. Fig 27 shows that reactive power injected into the grid is zero, i.e. real power (Fig 26) is injected into the grid at unity power factor, by the proposed controller, however FOPID method could not achieve nonzero reactive power initially but it is tending towards zero. Similarly, the proposed controller rendered the quadrature current, *i*_*q*_ almost zero, as shown in Fig 28. On the other hand, FOPID method has nonzero *i*_*q*_, however it is tending towards zero. In Fig 24, there is a reduction in PV output power due to change in temperature and irradiance, however PBSMC accurately tracks the reference value with the least overshoot. In Fig 25, there is a drop in the level of dc link voltage, however it gets restored after 1.2 s, in case of PBSMC. On the other hand FOPID and PBC have shown significant deviation and very weak convergence behavior. In Fig 26, there is a reduction in real power due to temperature and irradiance change, however, PBSMC has shown the maximum output real power of all. In Fig 27, FOPID has shown significant fluctuations in the reactive power. Also the reactive power is non-zero. Whereas in case of the remaining methods, the fluctuations are around the horizontal axis and are quite less in amplitude. In Fig 28, FOPID has shown significant fluctuations in the quadrature current. Also the quadrature current is non-zero. Whereas in case of the remaining methods, the fluctuations are around the horizontal axis and are quite less in amplitude with PBSMC being the least. In Fig 29, FOPID has shown significant fluctuations in the storage function. Also the value of storage function is non-zero in this case. Whereas in case of the remaining methods, the fluctuations are around the horizontal axis and are quite less in amplitude.

**Fig 30:**
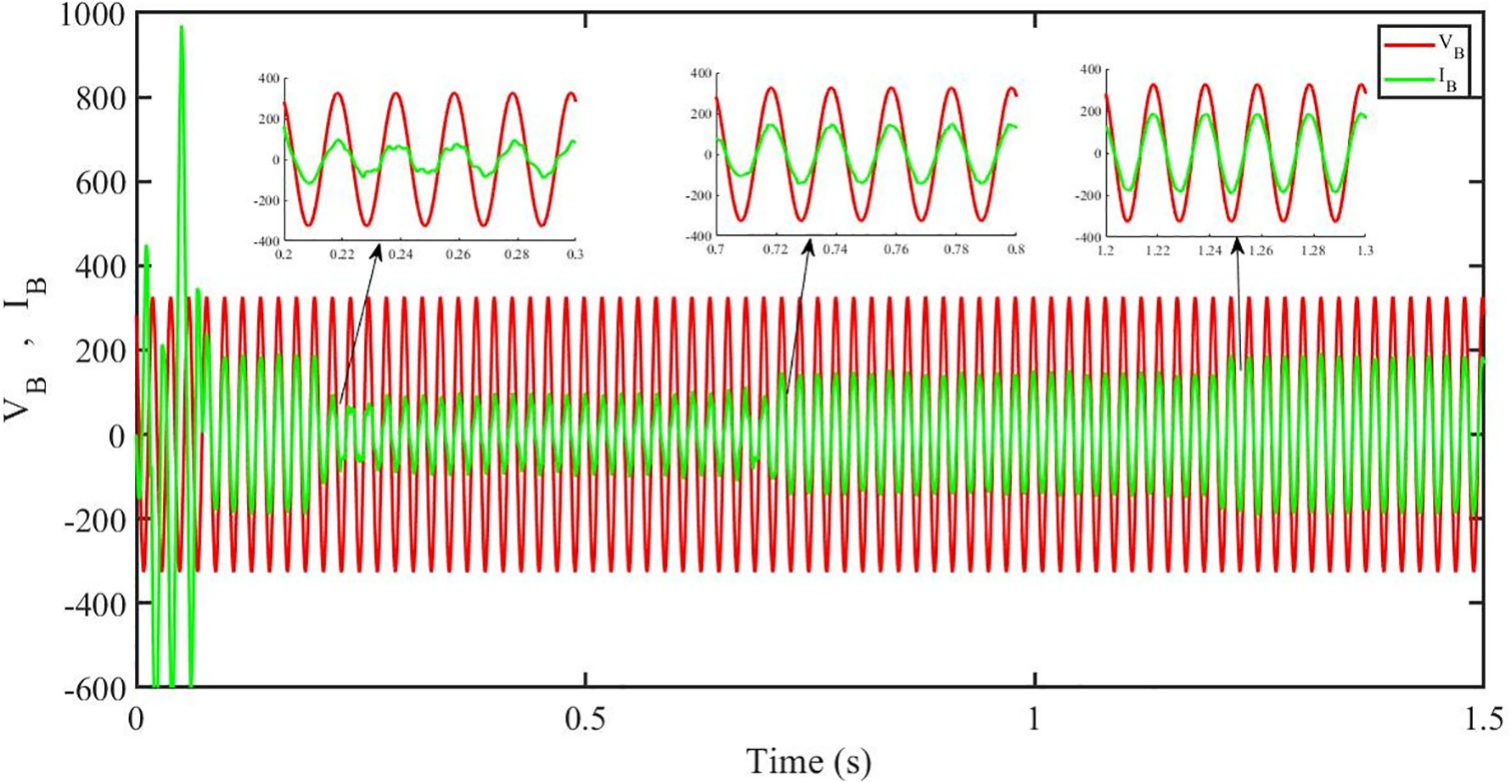
Grid side voltage and current corresponding to blue phase for FOPID.

Fig 30 shows grid side voltage and current corresponding to blue phase for FOPID under irradiance and temperature change. The current has reduced due to decrease in solar irradiance. After 1.2 s, current is at normal level in FOPID.

**Fig 31:**
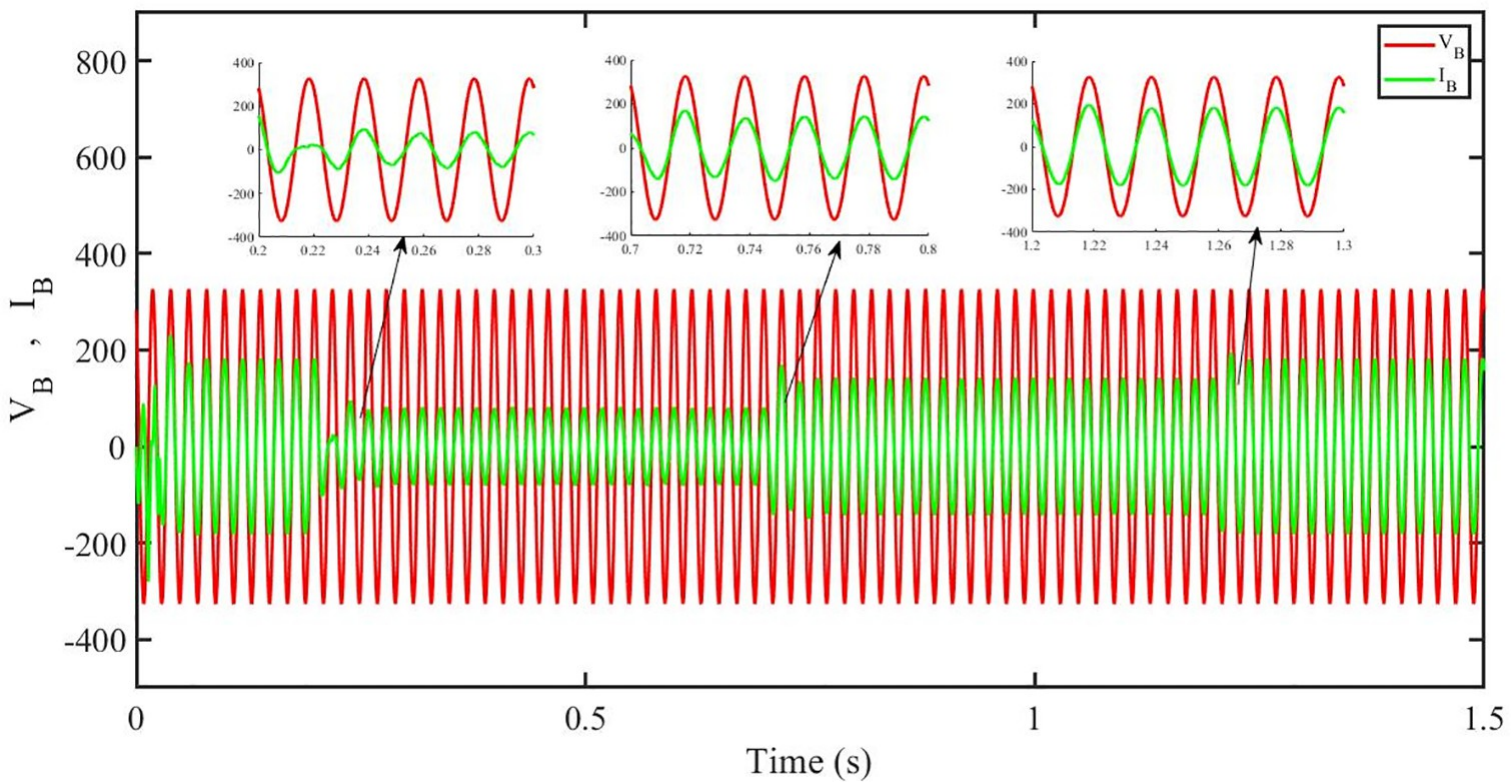
Grid side voltage and current corresponding to blue phase for PBC.

Fig 31 shows grid side voltage and current corresponding to blue phase for PBC under irradiance and temperature change. The current has reduced due to decrease in solar irradiance. After 1.2 s current is at normal level in PBC. As can be seen the current and voltage are in phase due to unity power factor.

**Fig 32:**
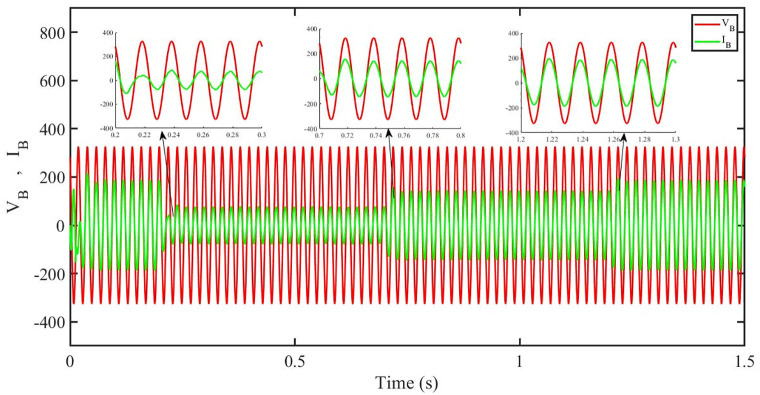
Grid side voltage and current corresponding to blue phase for PBFOPID.

Fig 32 shows grid side voltage and current corresponding to blue phase for PBFOPID under irradiance and temperature change. The current has reduced due to decrease in solar irradiance. After 1.2s current is at normal range in PBFOPID. As can be seen the current and voltage are in phase due to unity power factor.

**Fig 33:**
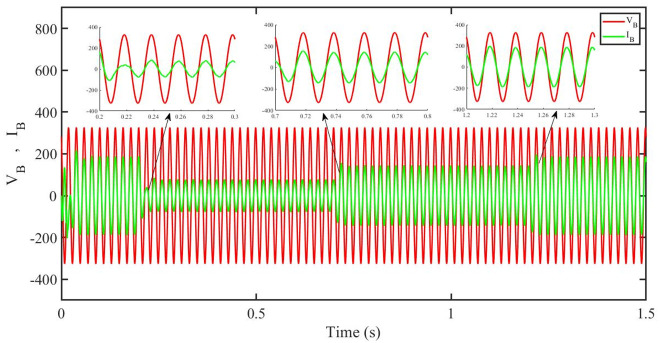
Grid side voltage and current corresponding to blue phase for PBSMC.

Fig 33 shows grid side voltage and current corresponding to blue phase for PBSMC under irradiance and temperature change. The current has reduced due to decrease in solar irradiance. After 1.2s current is at normal level in PBSMC and oscillations have reduced during initial phase. As can be seen the voltage and current are in phase due to quadrature current rendered to zero.

#### 5.3.1 Performance indices of PV output power under the change in solar irradiance and temperature

**Fig 34:**
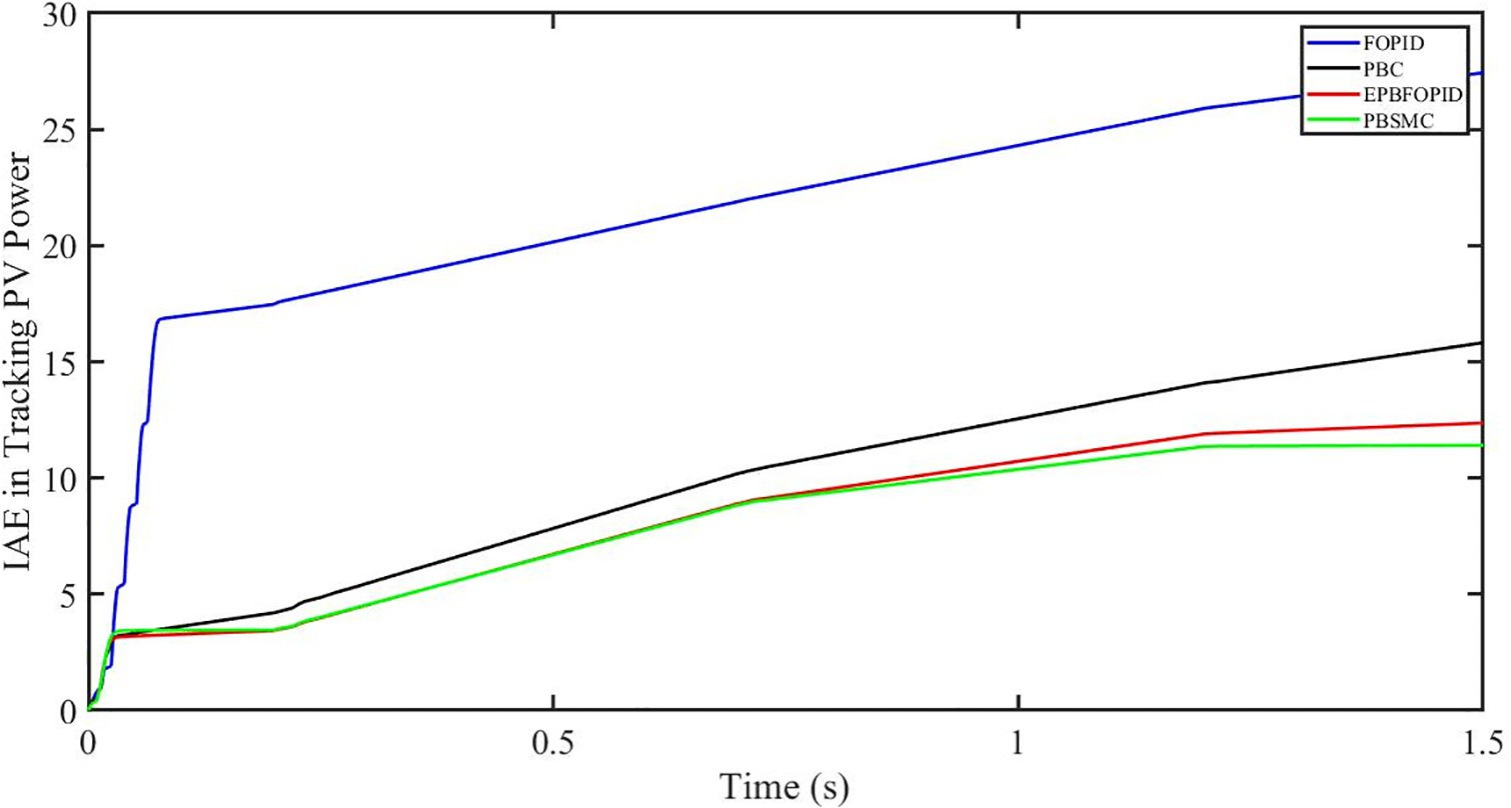
IAE in tracking PV power.

**Fig 35:**
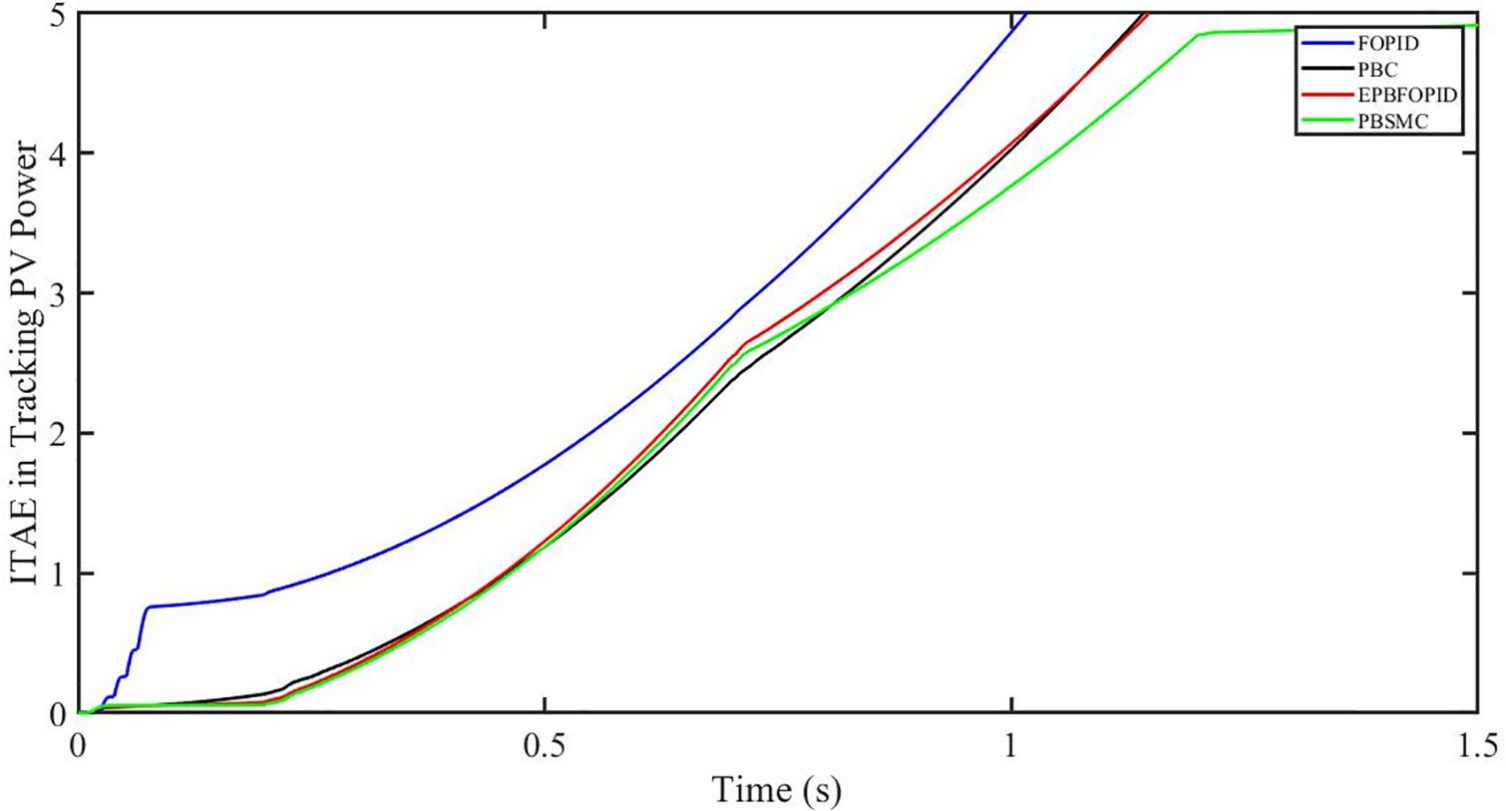
ITAE in tracking PV power.

**Fig 36:**
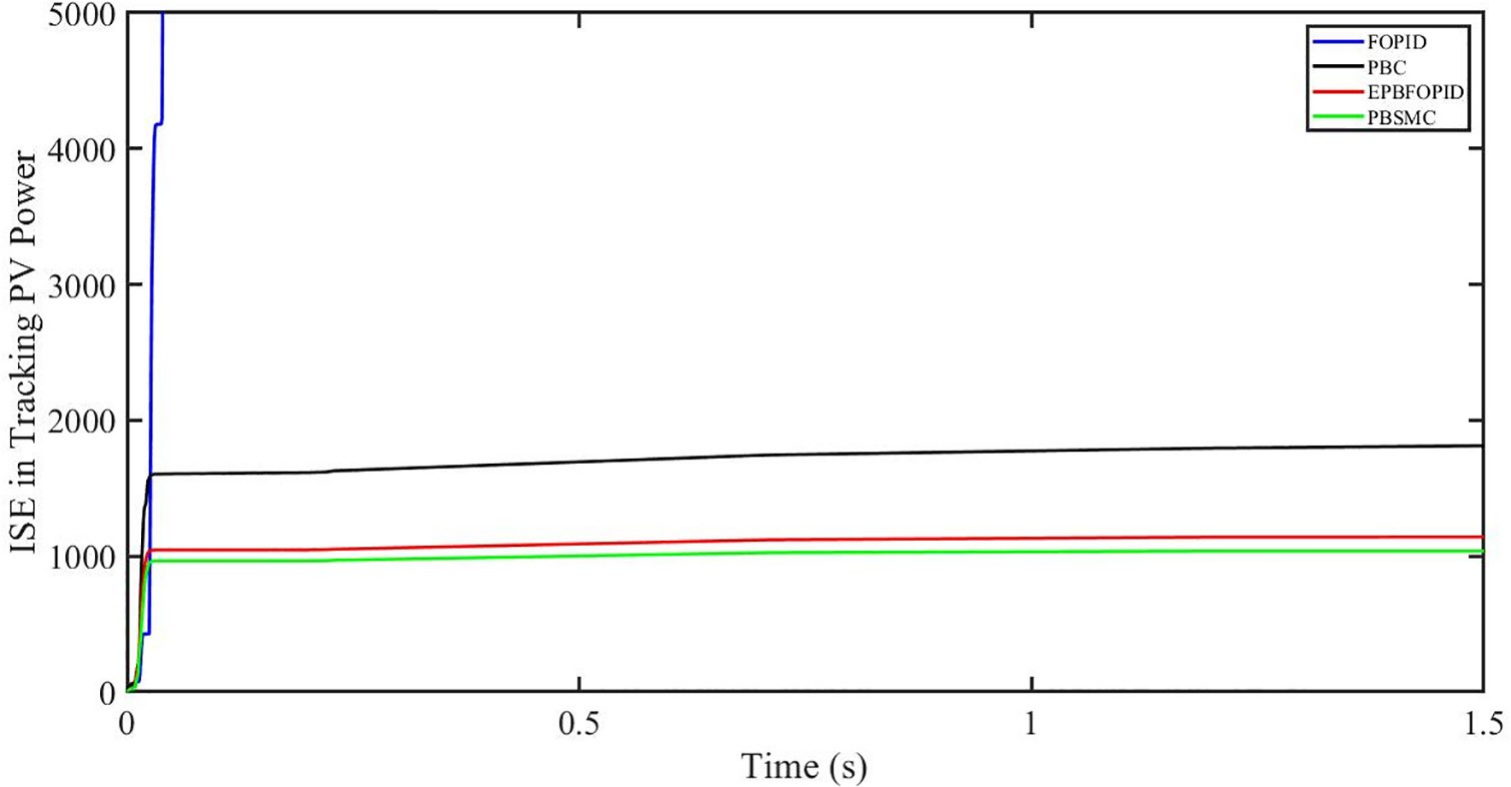
ISE in tracking PV power.

The performance indices of the four controllers, i.e. IAE, ITAE, and ISE indices are listed below. To investigate the whole operating range of the three cases, the simulation time T = 1.5 s was used. PBSMC has the lowest IAE, ITAE, and ISE indices for PV output power during solar irradiation and temperature change, as shown in Figs 34 to 36. As a result, it performs better than the other three controllers.

#### 5.3.2 Performance indices of dc-link voltage *V*_*dc*_ under the change in solar irradiance and temperature

**Fig 37:**
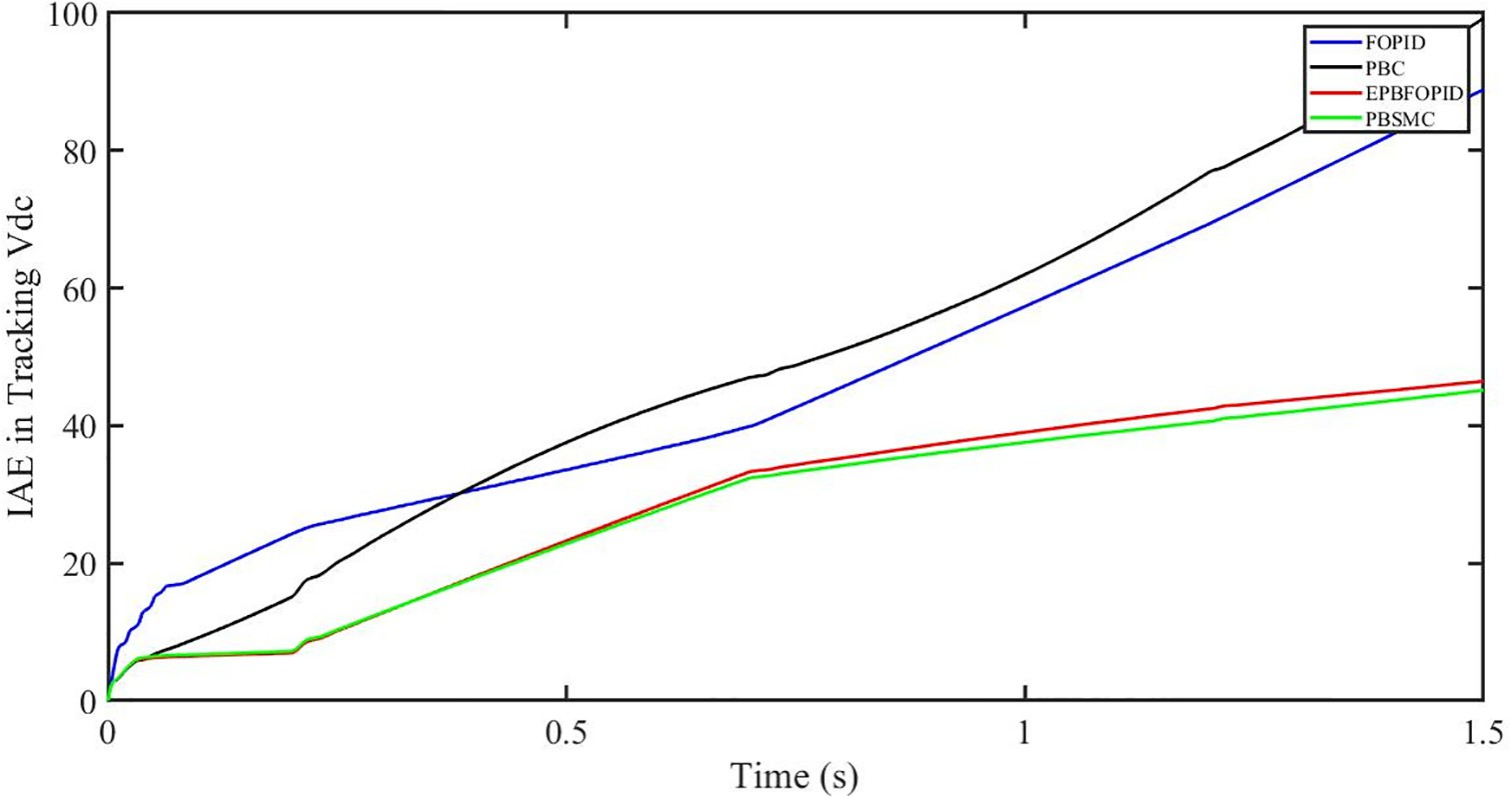
IAE in tracking *V*_*dc*_.

**Fig 38:**
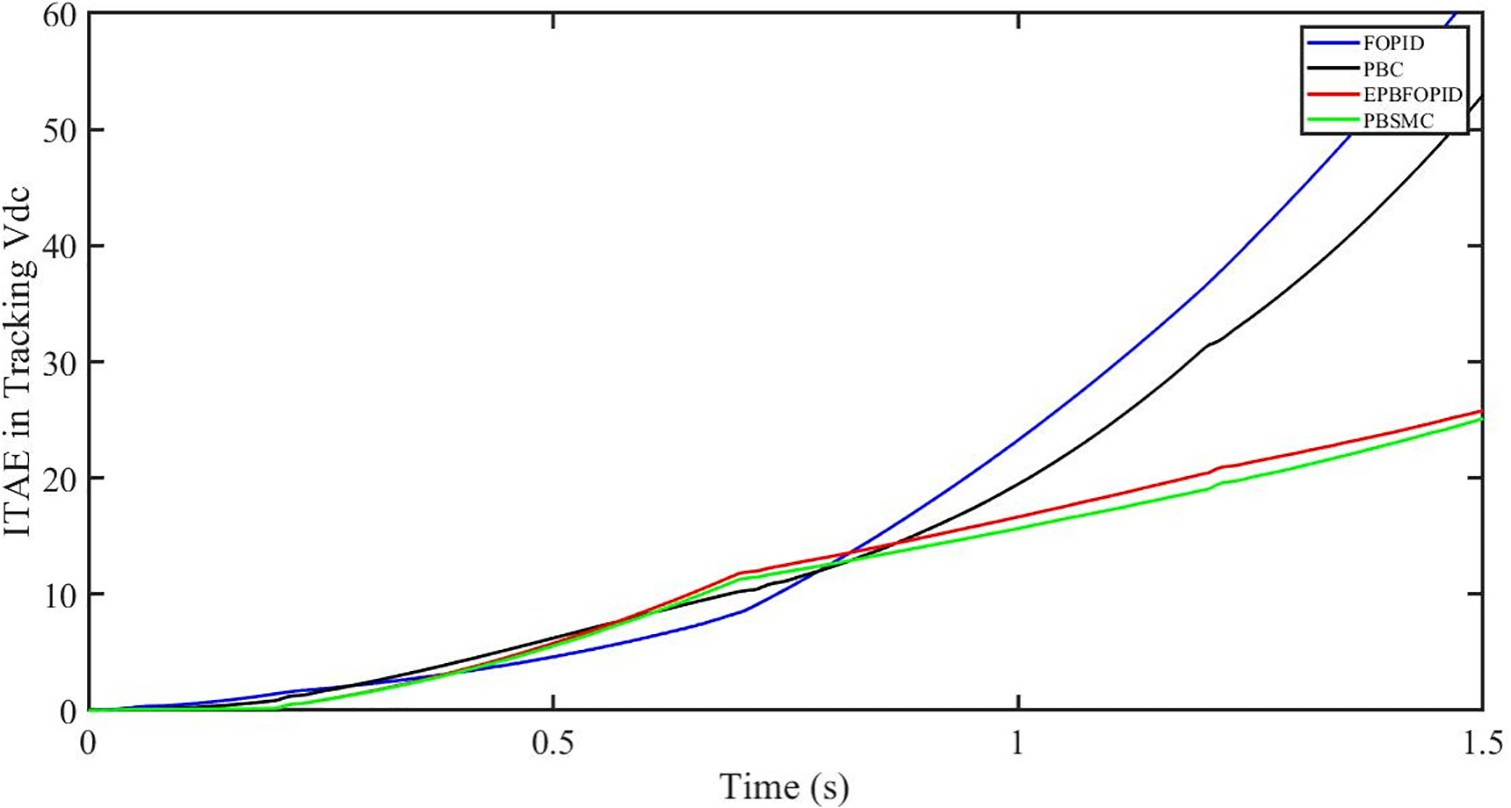
ITAE in tracking *V*_*dc*_.

**Fig 39:**
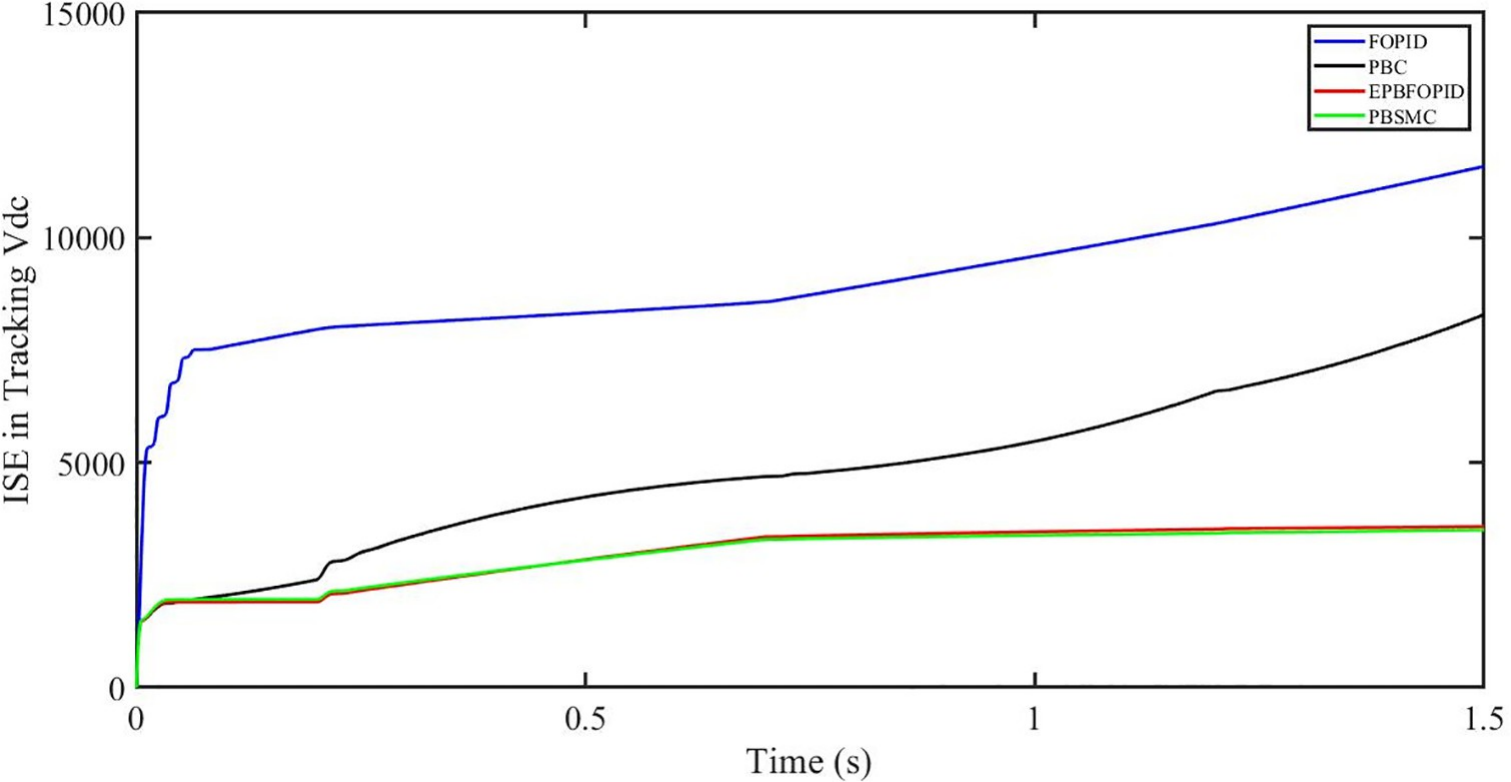
ISE in tracking *V*_*dc*_.

The performance indices of the four controllers, i.e. IAE, ITAE, and ISE are listed below. To investigate the whole operating range of the three cases, the simulation time T = 1.5 s was used. PBSMC has the lowest IAE, ITAE, and ISE indices for dc-link voltage V_dc_ during solar irradiation variation, as shown in Figs 37 to 39. As a result, it performs better than the other three controllers.

### 5.4 Power grid voltage drop

**Fig 40:**
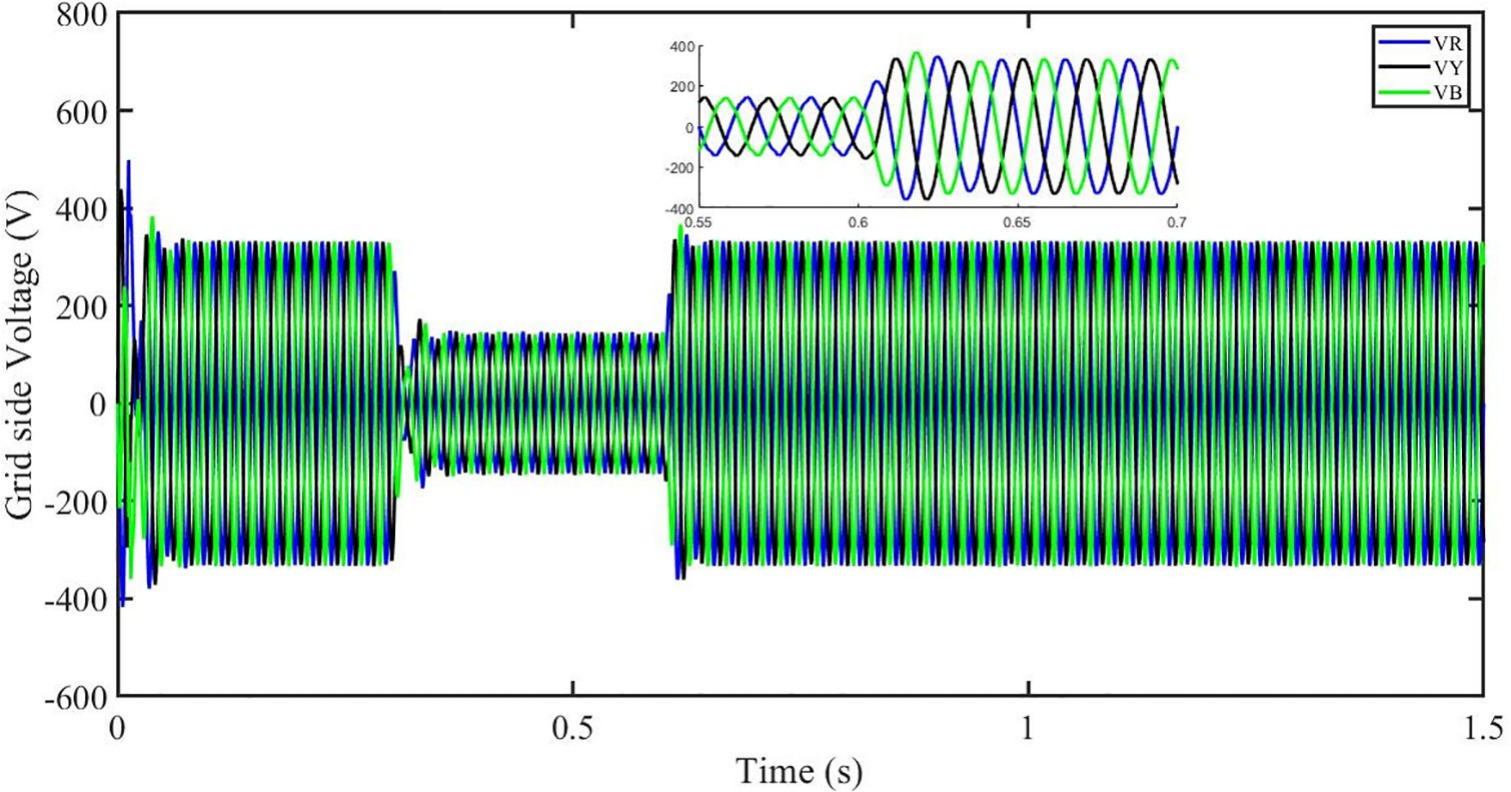
Three-phase voltages under fault ride through of a power grid.

**Fig 41:**
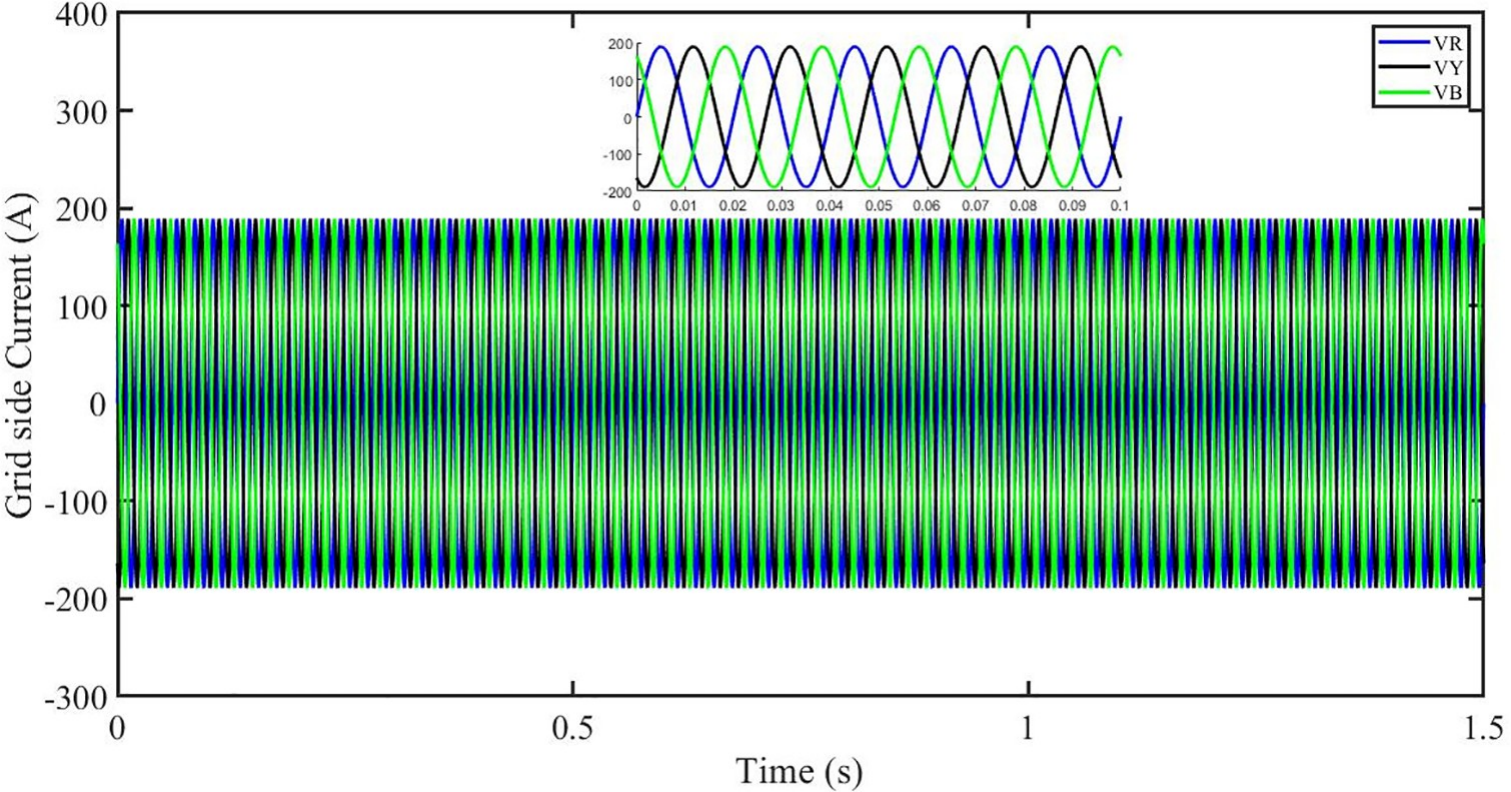
Three-phase currents under fault ride through of a power grid.

**Fig 42:**
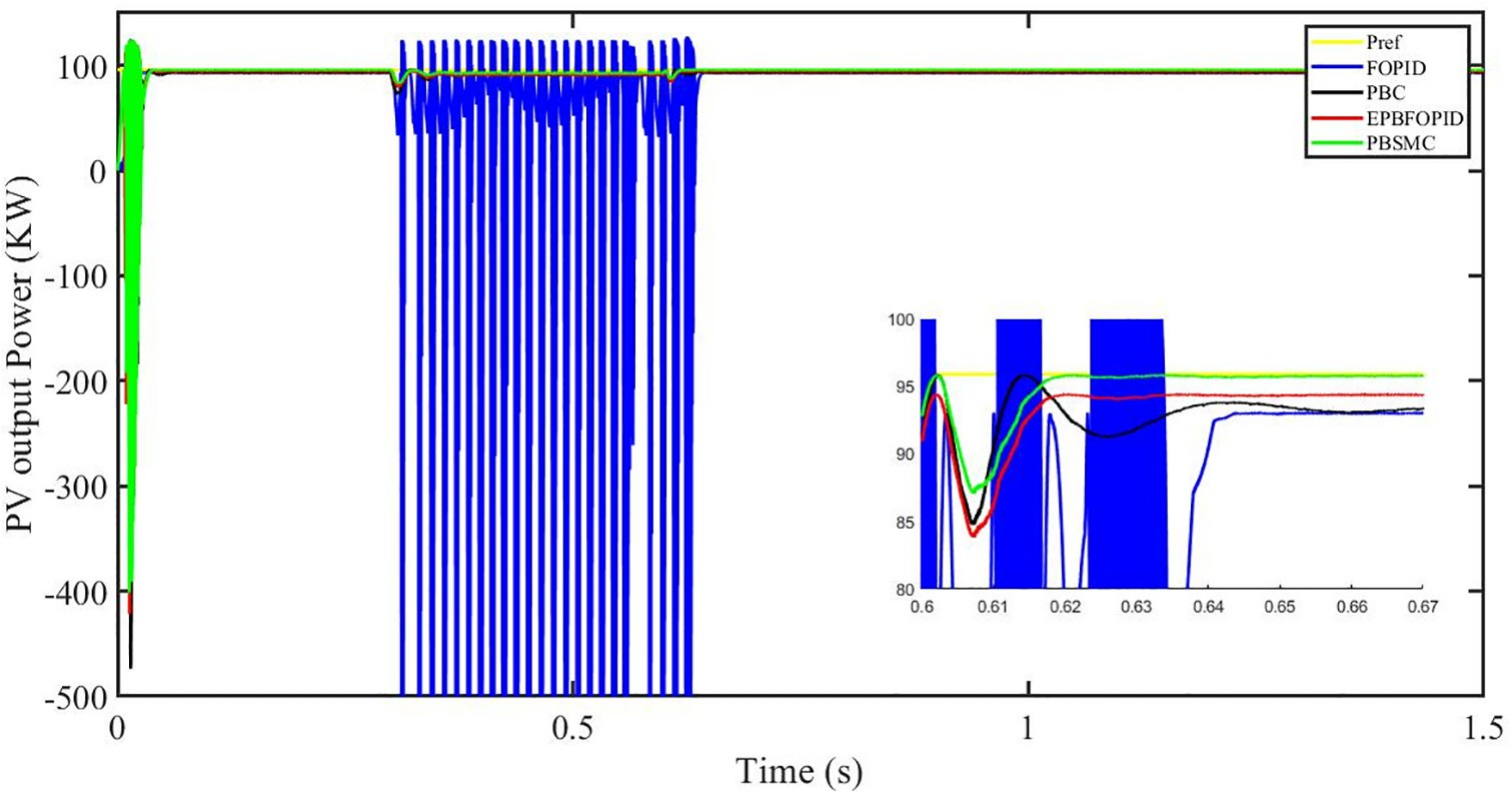
PV output power.

**Fig 43:**
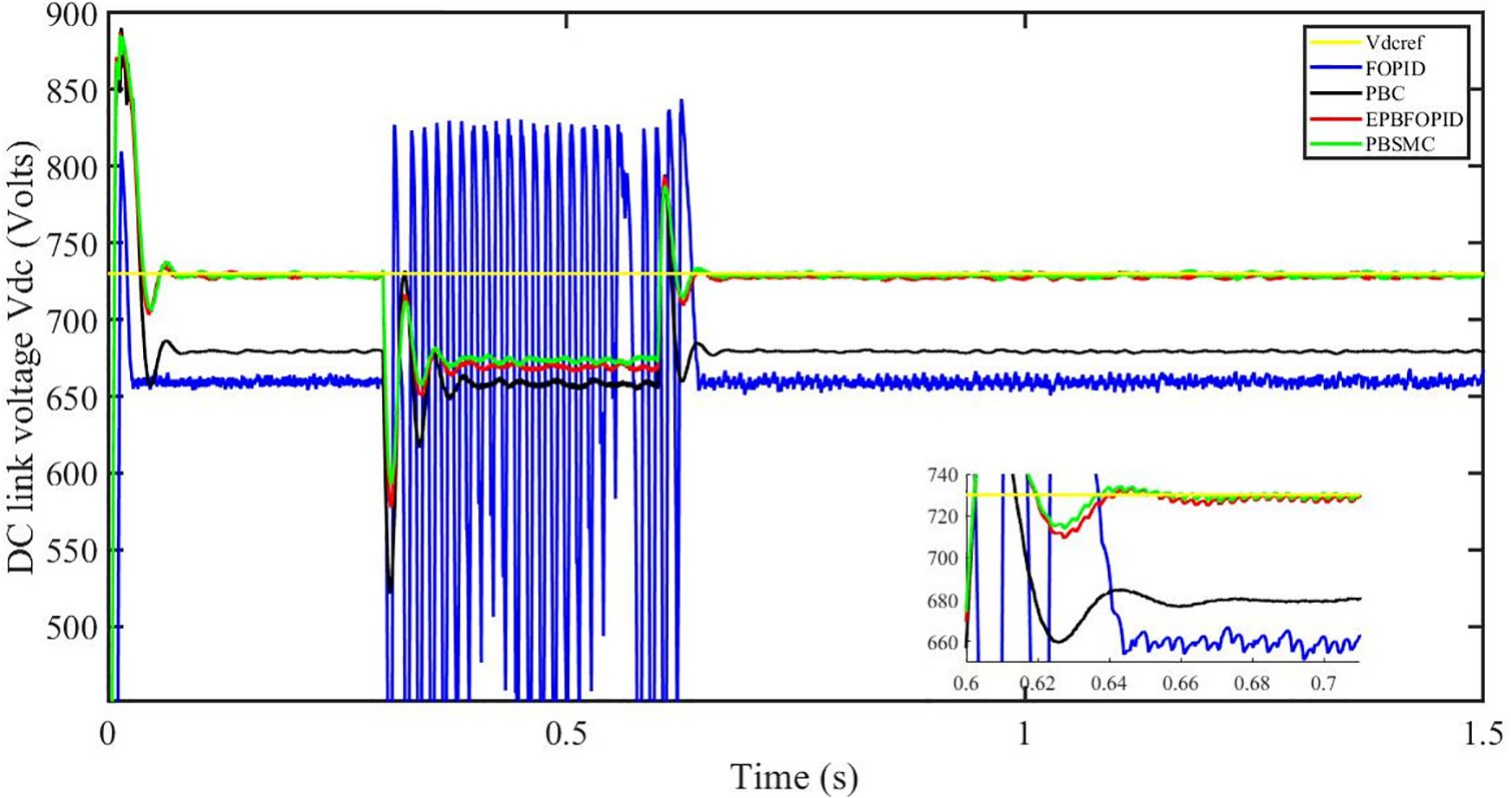
DC link voltage *V*_*dc*_.

**Fig 44:**
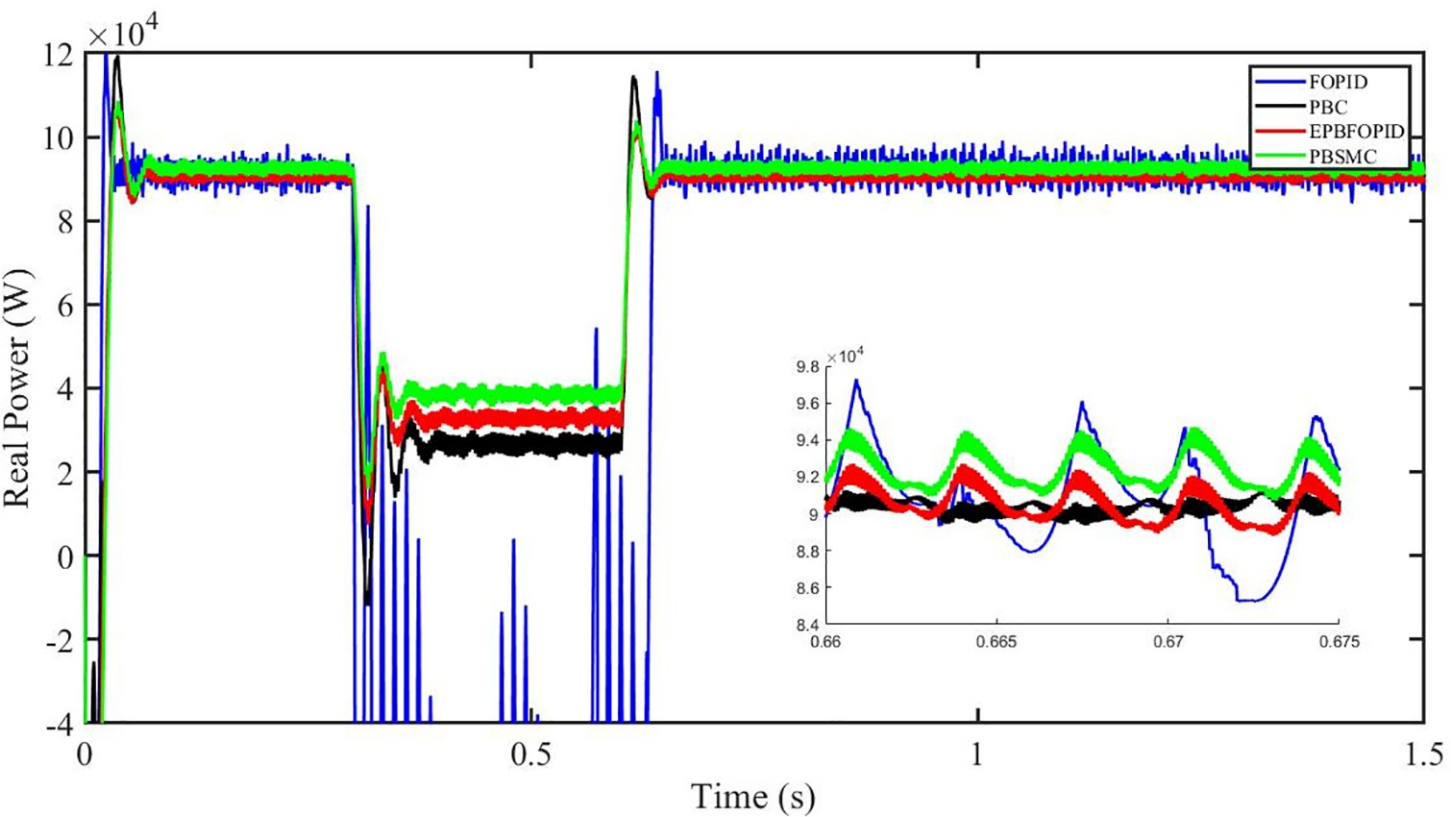
Real power (W).

**Fig 45:**
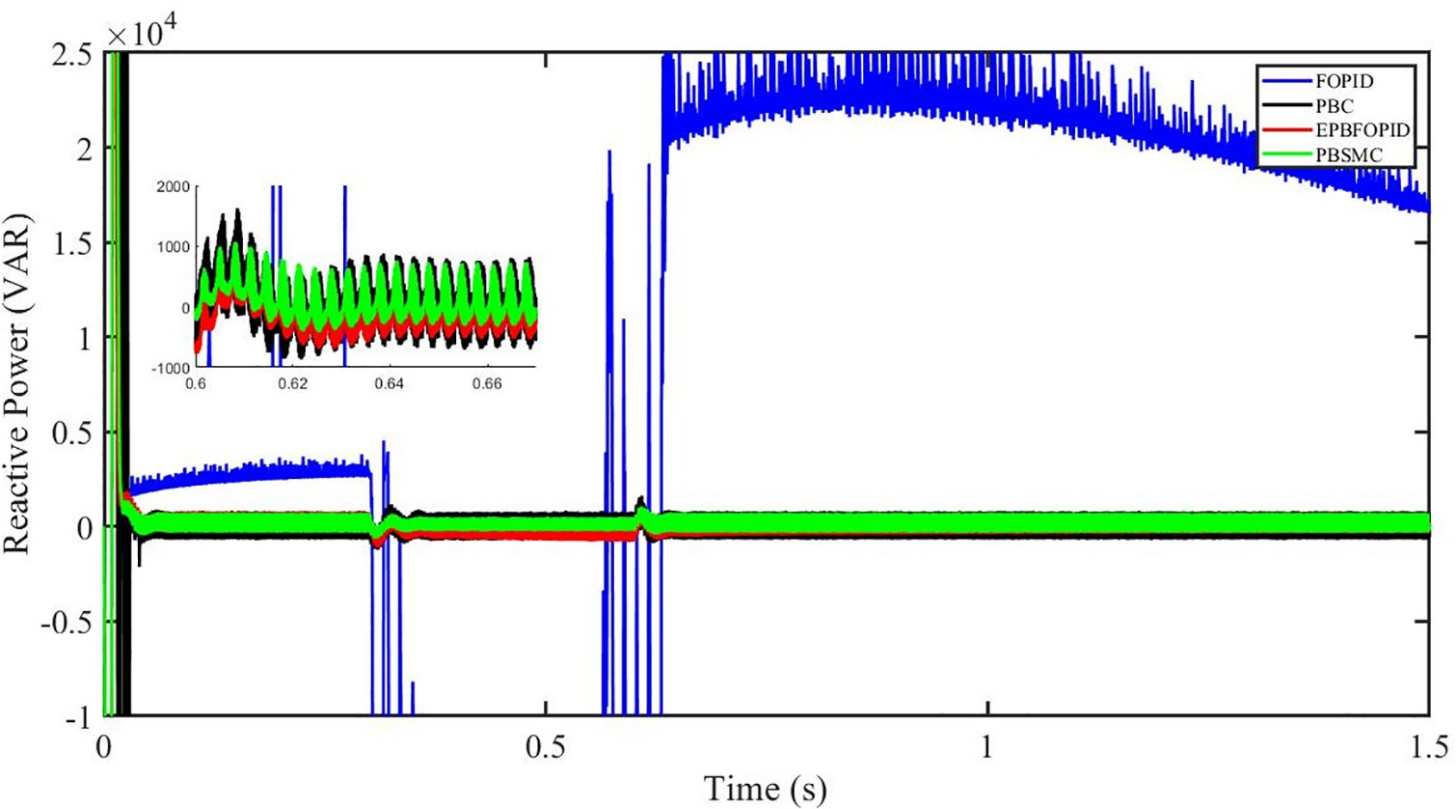
Reactive power (VAR).

**Fig 46:**
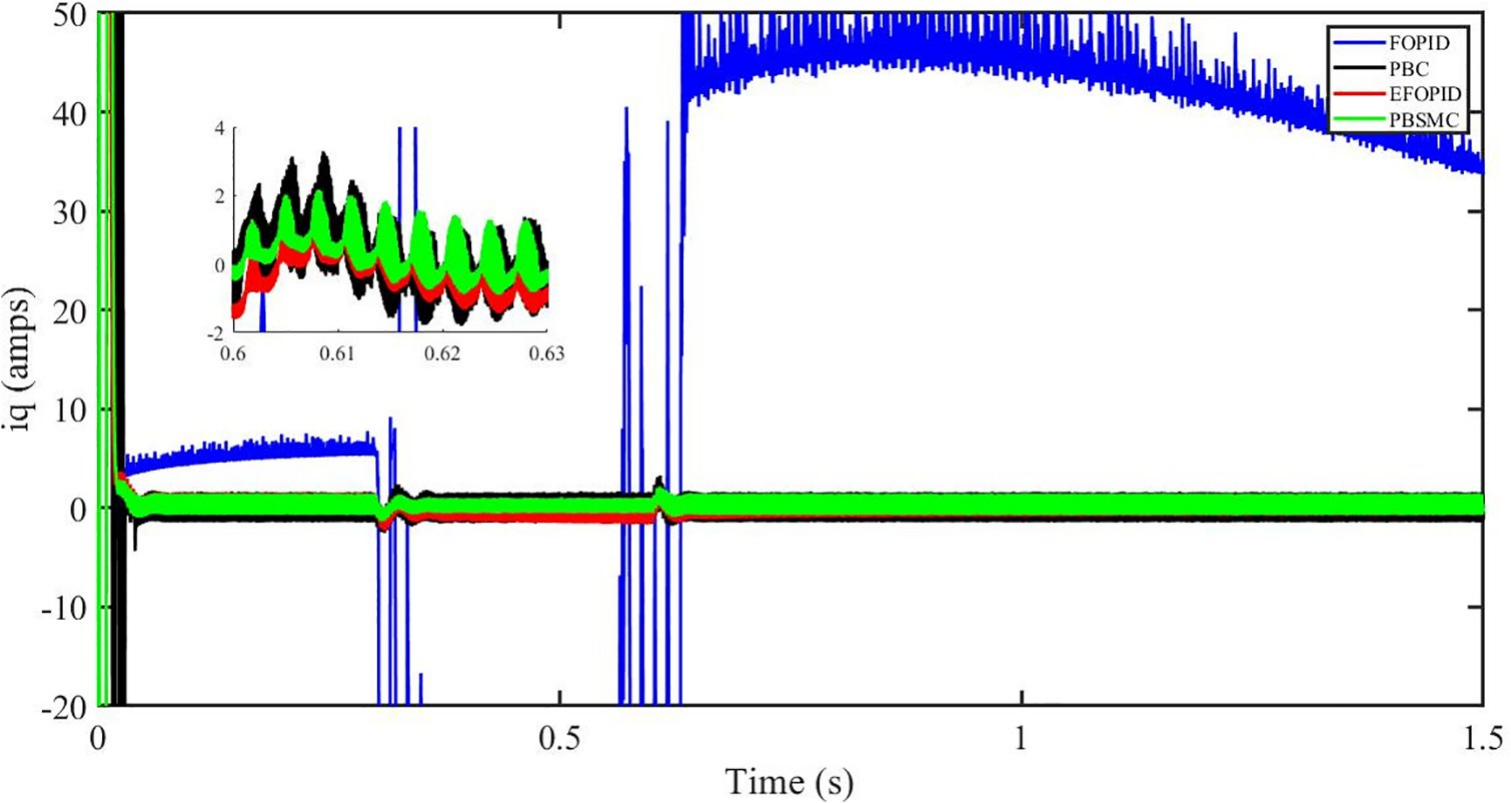
iq (amps).

**Fig 47:**
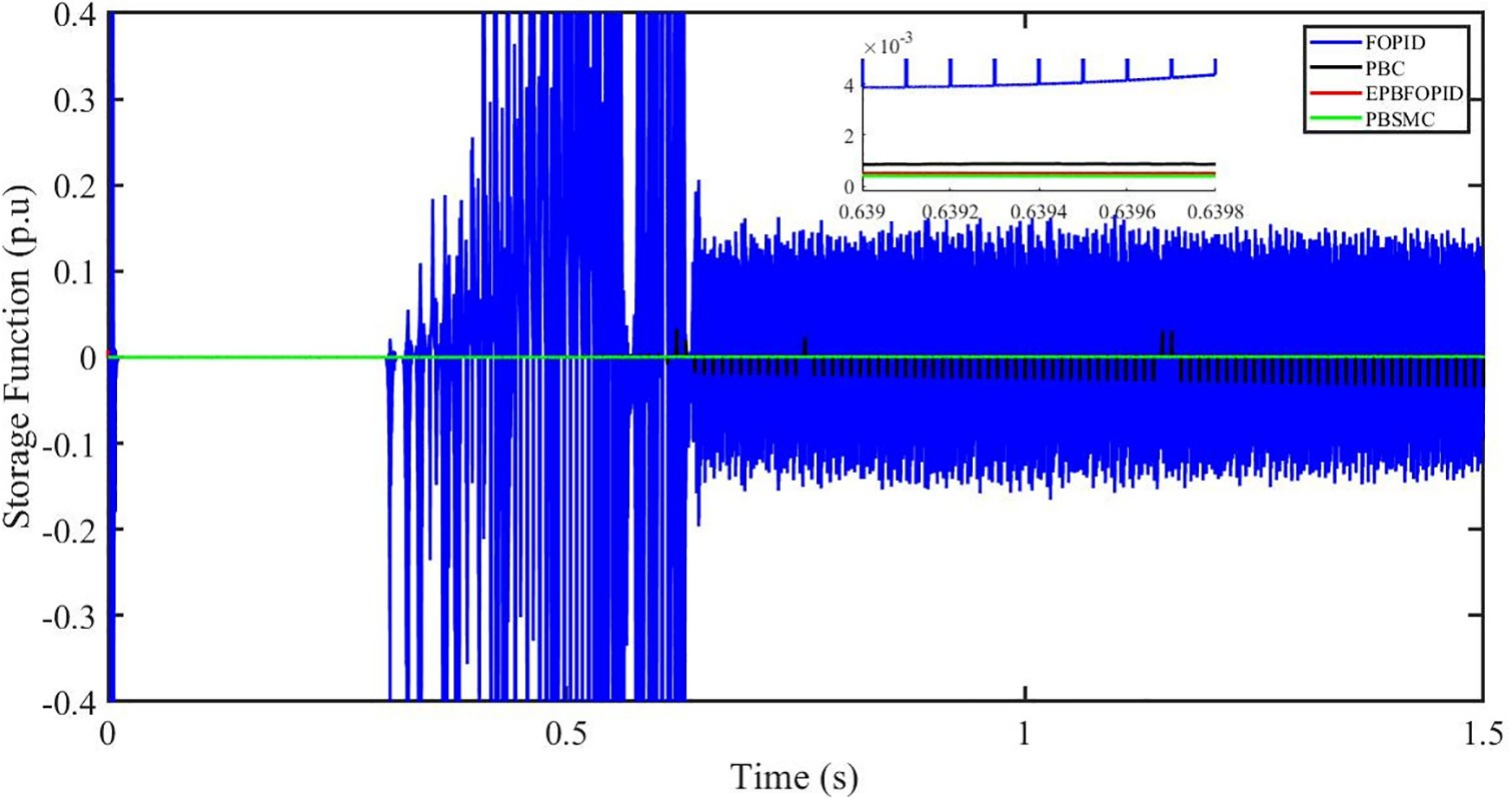
Storage function (p.u).

In the event of severe power grid voltage disruptions, fault ride-through (FRT) requires the PV system to remain connected and contribute to the power grid, since disconnection may impede voltage restoration during and after the fault [24, 26]. A power grid voltage decrease from its original value (t = 0.3s-0.6s) at the standard operating condition is used to assess the proposed approach’s FRT capacity shown in Figs 40 and 41. But the PBSMC is capable of removing oscillations from the output voltage, thus the voltage waveform looks quite sinusoidal. Hence, the proposed controller performance is quite effective under FRT. The relevant PV system responses are shown in Figs 42 to 47.

Figs 42 and 43 show the fast tracking of the reference by the proposed controller. Fig 9 shows that reactive power injected into the grid is zero, i.e. real power (Fig 44) is injected into the grid at unity power factor, by the proposed controller, however FOPID method could not achieve nonzero reactive power initially but it is tending towards zero. Similarly, the proposed controller rendered the quadrature current, *i*_*q*_ almost zero, as shown in Fig 46. On the other hand, FOPID method has nonzero *i*_*q*_, however it is tending towards zero.

In Fig 42, in case of FOPID, there are lot of oscillations in the PV output power in the fault interval, i.e. 0.3 s to 0.6 s, however they get stabilized after 0.6 s. Similarly in case of PBC, PBFOPID and PBSMC, there is a dip in the PV output power which is eliminated after 0.6 s. It needs to be mentioned here that the PBSMC has the least dip and PV output power converges fully to the reference power. In Fig 43, there is a reduction in the dc link voltage in the fault region interval, however after 0.6 s, PBSMC and PBFOPID get converged to the reference voltage, whereas FOPID and PBC do not get converged.

In Fig 44, real power gets reduced in the fault region, in all the four controllers, and is restored after the fault region. However there are still fluctuations in the real power after the fault region. In Fig 45, in case of FOPID, there are oscillations in the reactive power inside and outside the fault region. Also the reactive power is non-zero. However in the rest of the methods, i.e. PBC, PBFOPID and PBSMC, the reactive power is zero on the average, with some fluctuations. In Fig 46, in case of FOPID, there are oscillations in the quadrature current inside and outside the fault region. Also the quadrature current is non-zero. However in the rest of the methods, i.e. PBC, PBFOPID and PBSMC, the quadrature current is zero on the average, with some fluctuations. In Fig 47, in case of FOPID, there are large amplitude oscillations in the storage function, however, for the rest of the methods they are quite less in amplitude and the storage function is almost zero in case of PBSMC.

PBSMC can restore active power, DC-link voltage, and q-axis current produced by the fault at the fastest rate and with the fewest oscillations. This may also be validated by looking at how the storage function changes, for example, PBSMC can produce a small energy magnitude shift and quick energy dissipation.

**Fig 48:**
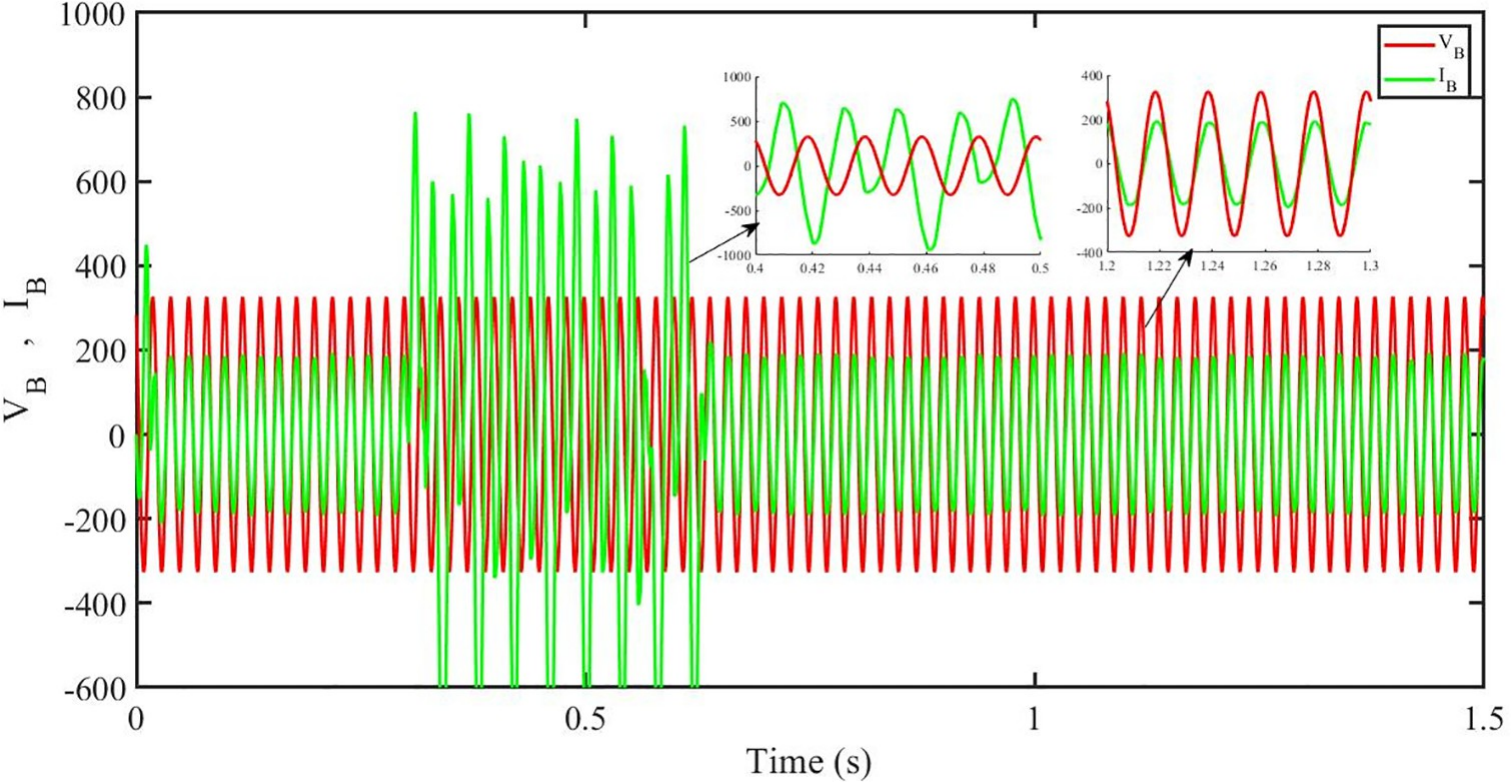
Grid side voltage and current corresponding to blue phase for FOPID.

Fig 48 shows grid side voltage and current corresponding to blue phase for FOPID under FRT. In the time interval, 0.3 s to 0.6 s, the current has increased from its normal value and also is out of phase from the voltage, however after 0.6 s, the current attains its normal amplitude and gets in phase with the voltage.

**Fig 49:**
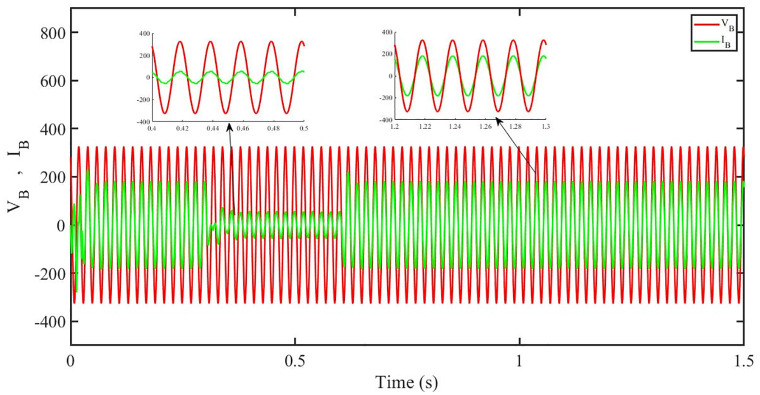
Grid side voltage and current corresponding to blue phase for PBC.

Fig 49 shows grid side voltage and current corresponding to blue phase for PBC under FRT. In this case the current amplitude has reduced in the fault interval, 0.3 s to 0.6 s, however after 0.6 s, the current comes back to its normal amplitude. Also the current is in phase with the voltage.

**Fig 50:**
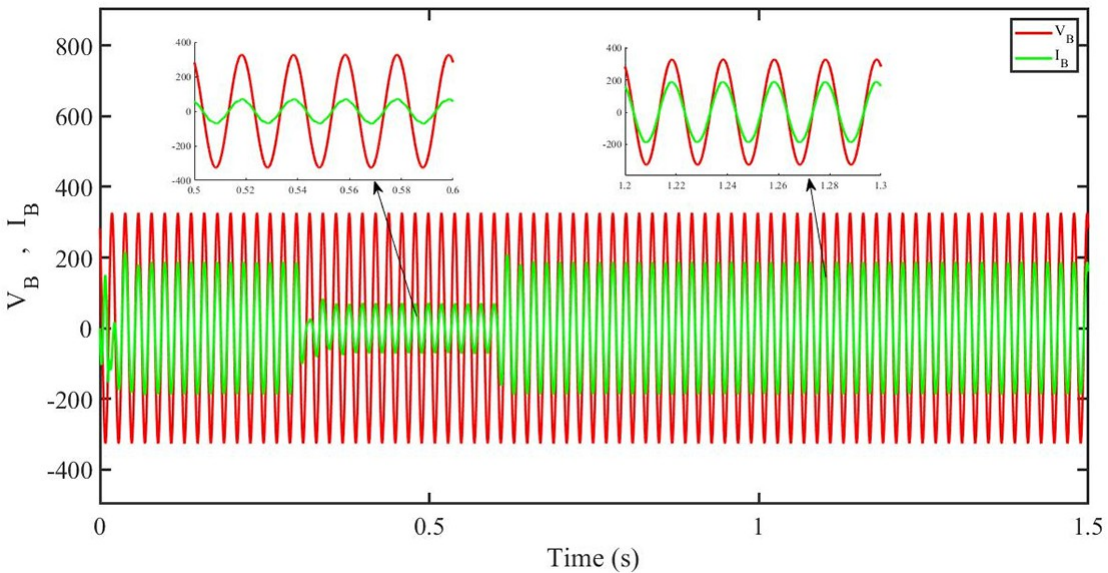
Grid side voltage and current corresponding to blue phase for PBFOPID.

Fig 50 shows grid side voltage and current corresponding to blue phase for PBFOPID under FRT. Similar to the previous case, in this case, the current amplitude has reduced in the fault interval, 0.3 s to 0.6 s, however after 0.6 s, the current comes back to its normal amplitude. Also the current is in phase with the voltage.

**Fig 51:**
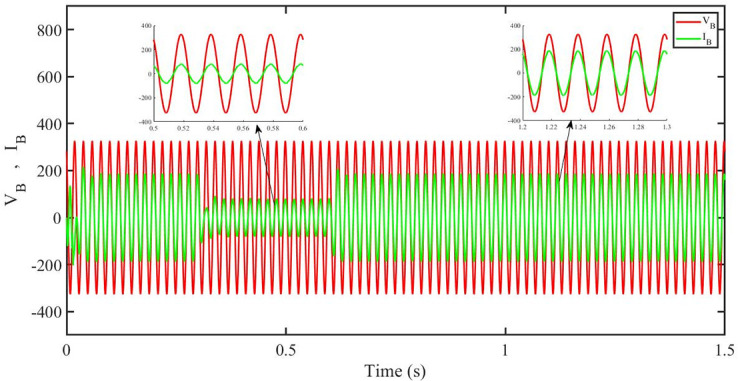
Grid side voltage and current corresponding to blue phase for PBSMC.

Fig 51 shows grid side voltage and current corresponding to blue phase for PBSMC under FRT. In this case the current amplitude has reduced in the fault interval, 0.3 s to 0.6 s, however after 0.6 s, the current comes back to its normal amplitude. Also the current is in phase with the voltage.

#### 5.4.1 Performance indices of PV output power under fault ride through a power grid

**Fig 52:**
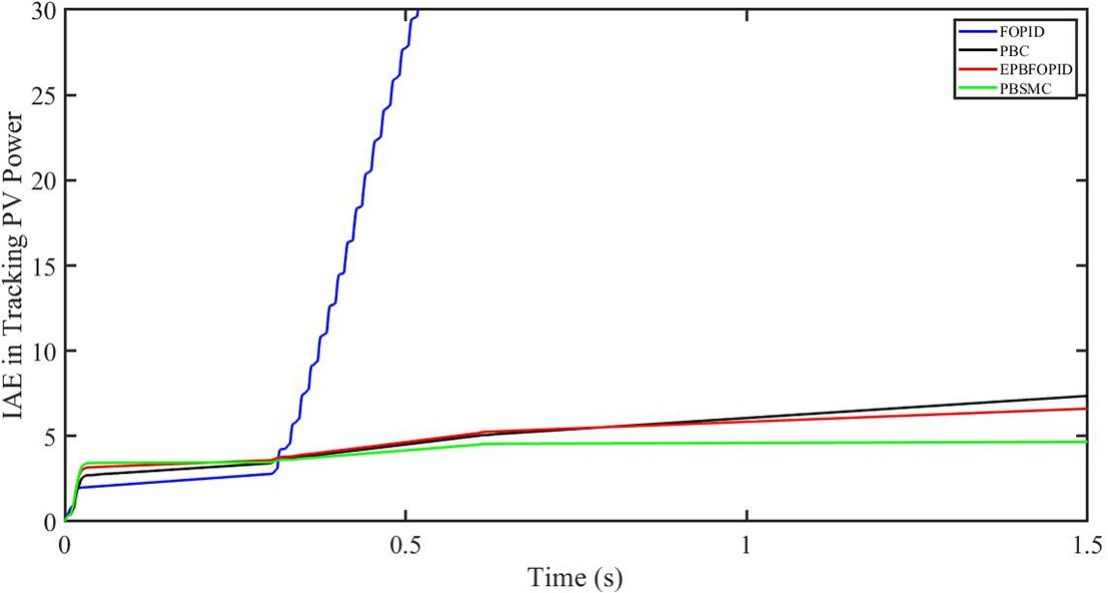
IAE in tracking PV power.

**Fig 53:**
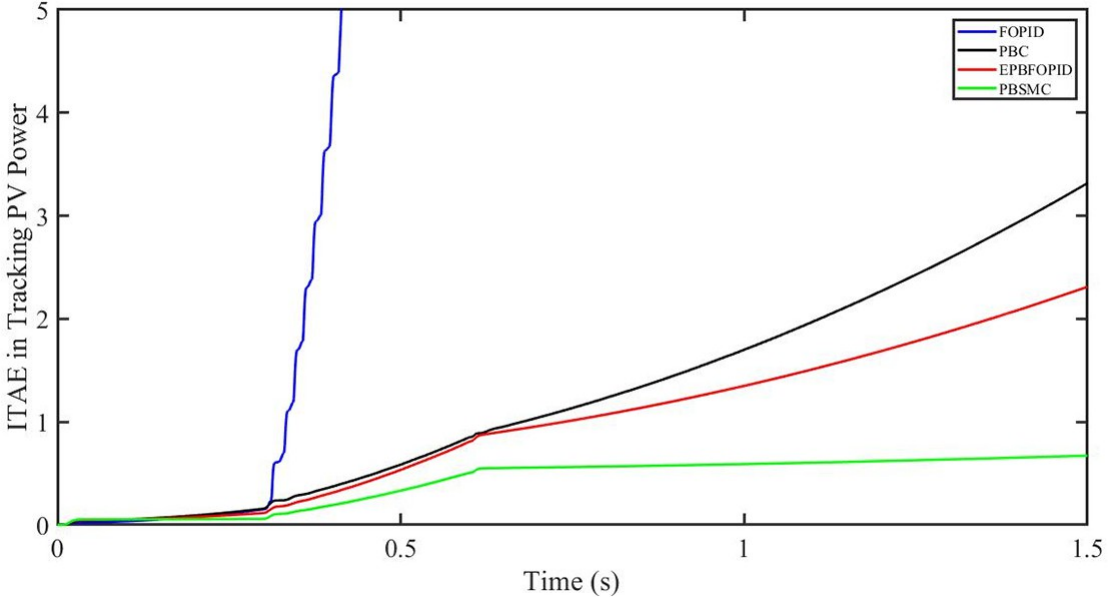
ITAE in tracking PV power.

**Fig 54:**
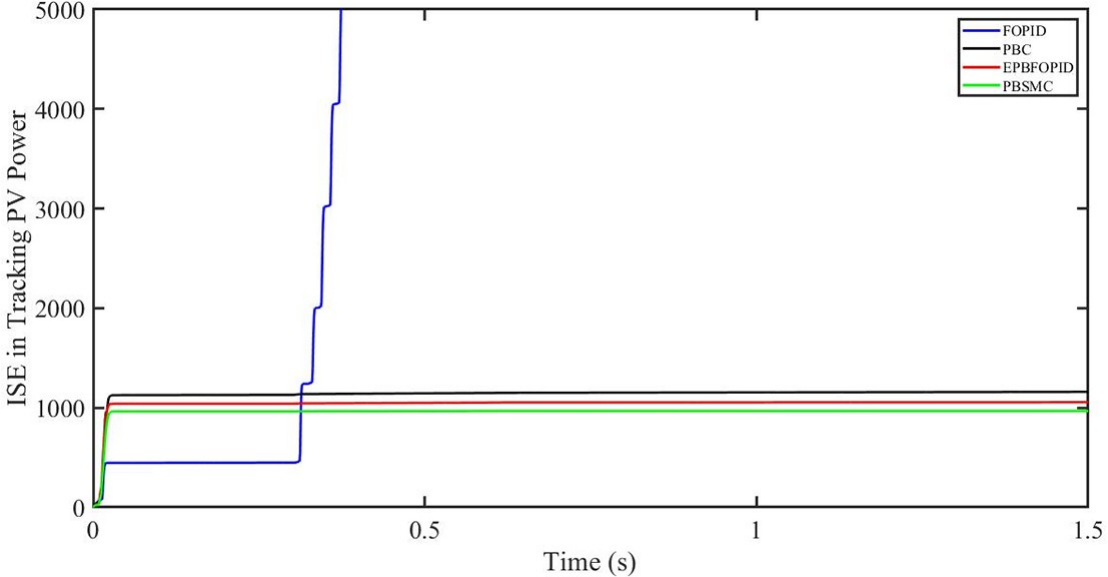
ISE in tracking PV power.

Performance indices of the four controllers, i.e. IAE, ITAE, and ISE are listed below. To investigate the whole operating range of three cases, the simulation time T = 1.5 s was used. PBSMC has the lowest IAE, ITAE, and ISE indices for PV output power during fault ride through to the power grid, as shown in Figs 52 to 54. As a result, it performs better than the other three controllers.

#### 5.4.2 Performance indices of dc-link voltage *V*_*dc*_ under fault ride through a power grid

**Fig 55:**
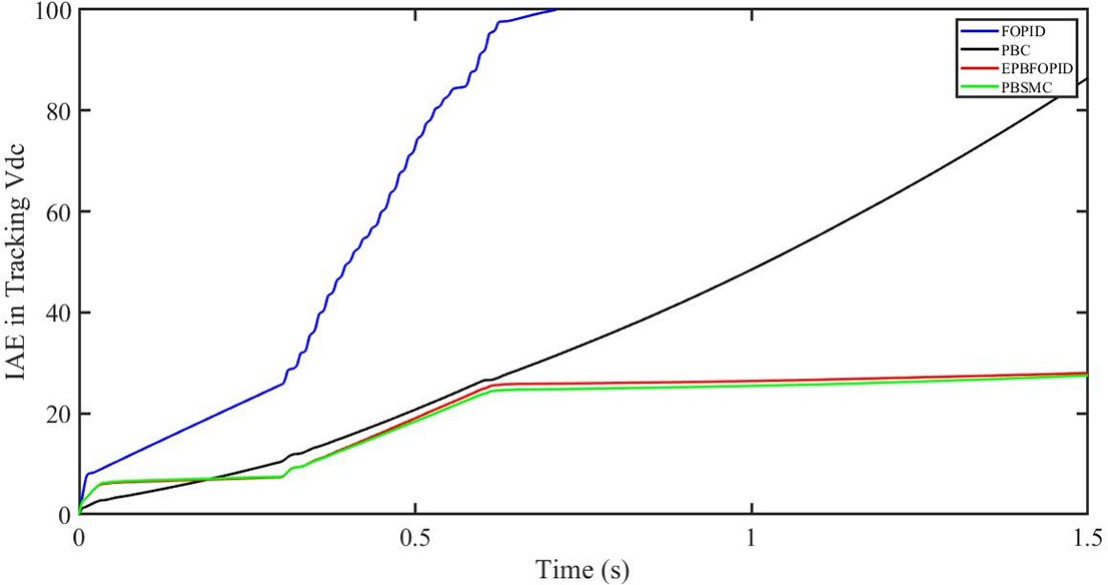
IAE in tracking *V*_*dc*_.

**Fig 56:**
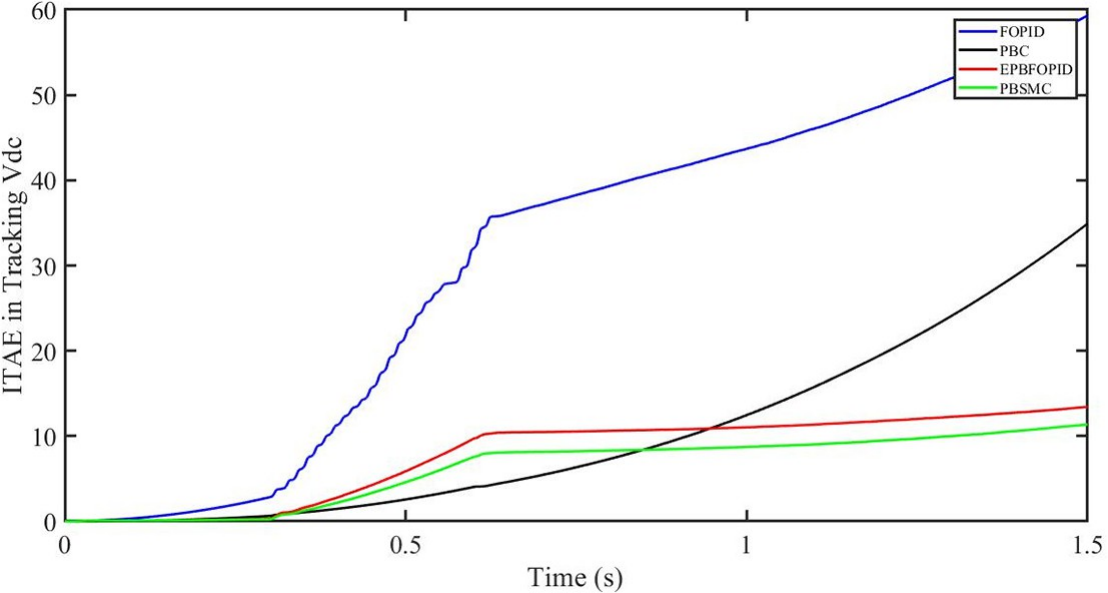
ITAE in tracking *V*_*dc*_.

**Fig 57:**
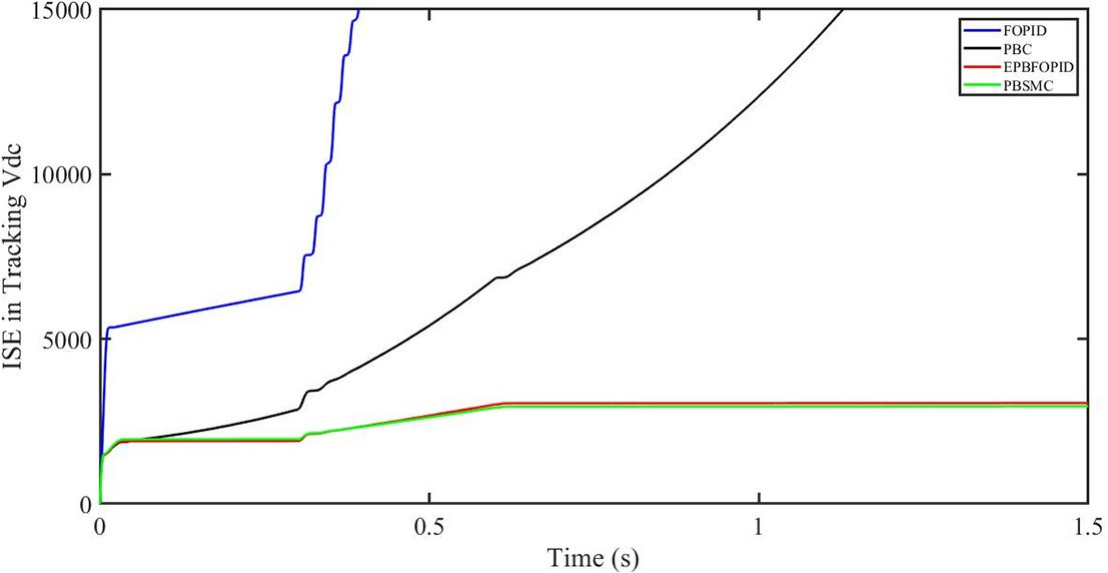
ISE in tracking *V*_*dc*_.

Performance indices of the four controllers, i.e. IAE, ITAE, and ISE are listed below. To investigate the whole operating range of three cases, the simulation time T = 1.5 s was used. PBSMC has the lowest IAE, ITAE, and ISE indices for dc-link voltage *V*_*dc*_ during fault ride through at the power grid, as shown in Figs 55 to 57. As a result, it performs better than the other three controllers.

Tables 5–7 show total values (integrated values of errors over the simulation time) of the performance indicators IAE, ITAE and ISE corresponding to the three cases. As can be seen, values corresponding to PBSMC are the least of all values.

**Table 5:** IAE indices of four controllers obtained in three cases.

Cases	IAE indices	FOPID	PBFOPID	PBC	PBSMC
**Solar irradiance change**	IAE_P_	21	5.5	6.5	4
	IAE_Vdc_	100	48	78	45
**Solar irradiance and temperature change**	IAE_P_	27	12.5	16	11
	IAE_Vdc_	88	45	100	43
**Power grid voltage drop**	IAE_P_	30	6.5	7.5	5
	IAE_Vdc_	100	28	87	25

**Table 6:** ITAE indices of four controllers obtained in three cases.

Cases	ITAE indices	FOPID	PBFOPID	PBC	PBSMC
**Solar irradiance change**	ITAE_P_	5	1.8	2.5	0.5
	ITAE_Vdc_	60	28	48	25
**Solar irradiance and temperature change**	ITAE_P_	5	4	4	3.5
	ITAE_Vdc_	60	26	53	23
**Power grid voltage drop**	ITAE_P_	5	2.2	3.2	0.8
	ITAE_Vdc_	60	13	35	10.5

**Table 7:** ISE indices of four controllers obtained in three cases.

Cases	ISE indices	FOPID	PBFOPID	PBC	PBSMC
**Solar irradiance change**	ISE_P_	5000	1050	1300	1000
	ISE_Vdc_	12500	3600	11500	3500
**Solar irradiance and temperature change**	ISE_P_	5000	1100	1800	1000
	ISE_Vdc_	11500	3500	8000	3300
**Power grid voltage drop**	ISE_P_	5000	1050	1150	950
	ISE_Vdc_	15000	3000	15000	2800

### 5.5 Total harmonic distortion (THD)

The total harmonic distortion [49] is a measure of the amount of harmonic distortion present in a signal and is defined as the ratio of sum of powers of all the harmonic components to the power of the fundamental frequency. Mathematically,

$$THD=\frac{\sqrt{\left(V_{2}^{2}+V_{3}^{2}+V_{4}^{2}+\ldots \right)}}{V_{1}}$$ (63)

where, *V*_*n*_ is the RMS value of the n^th^ harmonic voltage and *V*_1_ is the RMS value of the fundamental component.

#### 5.5.1 Solar irradiance change

**Fig 58:**
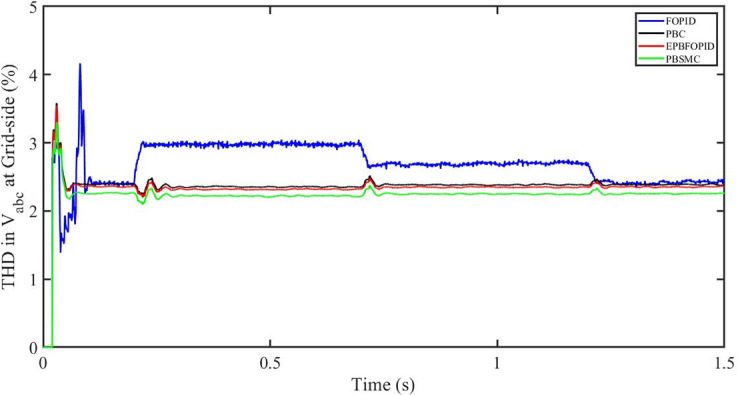
THD in V_abc_ (voltage) at grid side for variable irradiance.

**Fig 59:**
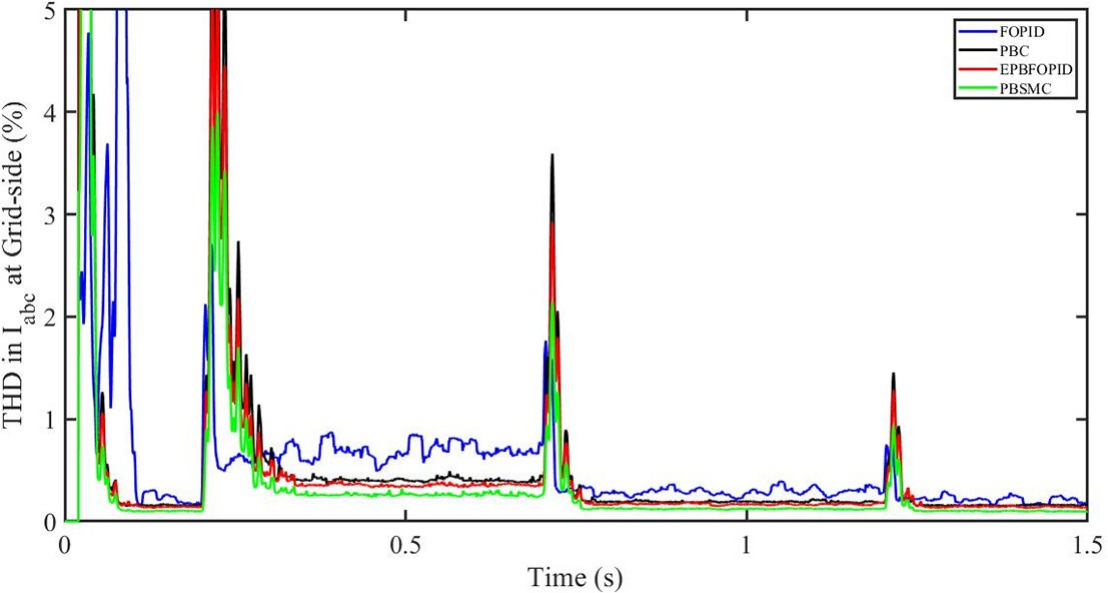
THD in I_abc_ (current) at grid side corresponding to variable irradiance.

Fig 58 shows the THD in grid side voltage corresponding to the four control systems, FOPID, PBFOPID, PBC and PBSMC with variable irradiance and constant temperature. As can be seen, proposed PBSMC has least THD. Similarly Fig 59 shows THD in grid side current corresponding to the four control systems. Again as can be seen proposed PBSMC has least THD.

#### 5.5.2 Solar irradiance and temperature change

**Fig 60:**
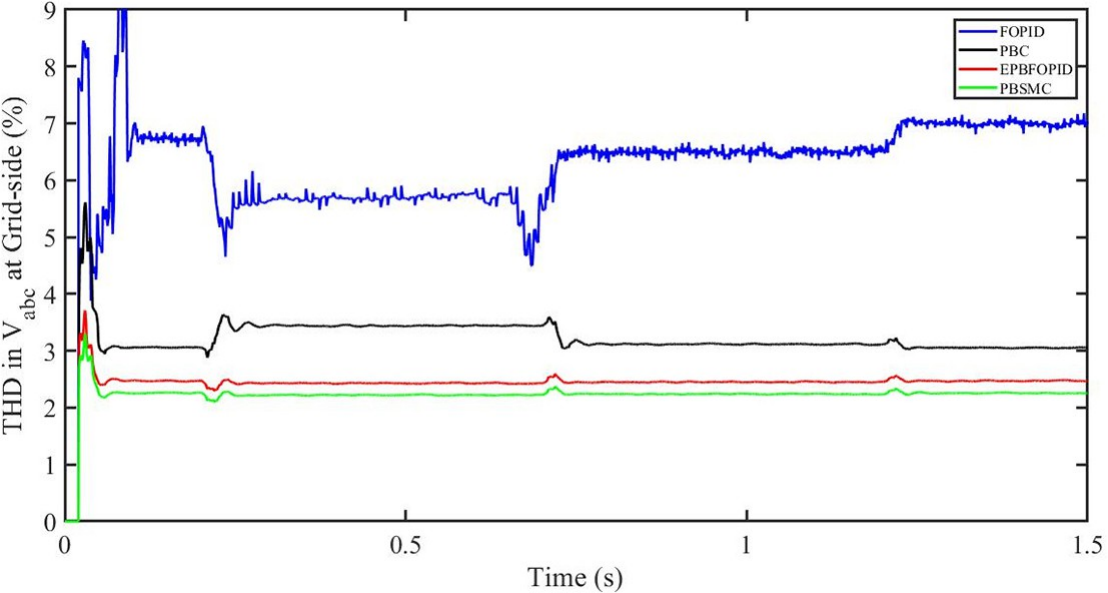
THD in V_abc_ (voltage) at grid side corresponding to variable irradiance and temperature.

**Fig 61:**
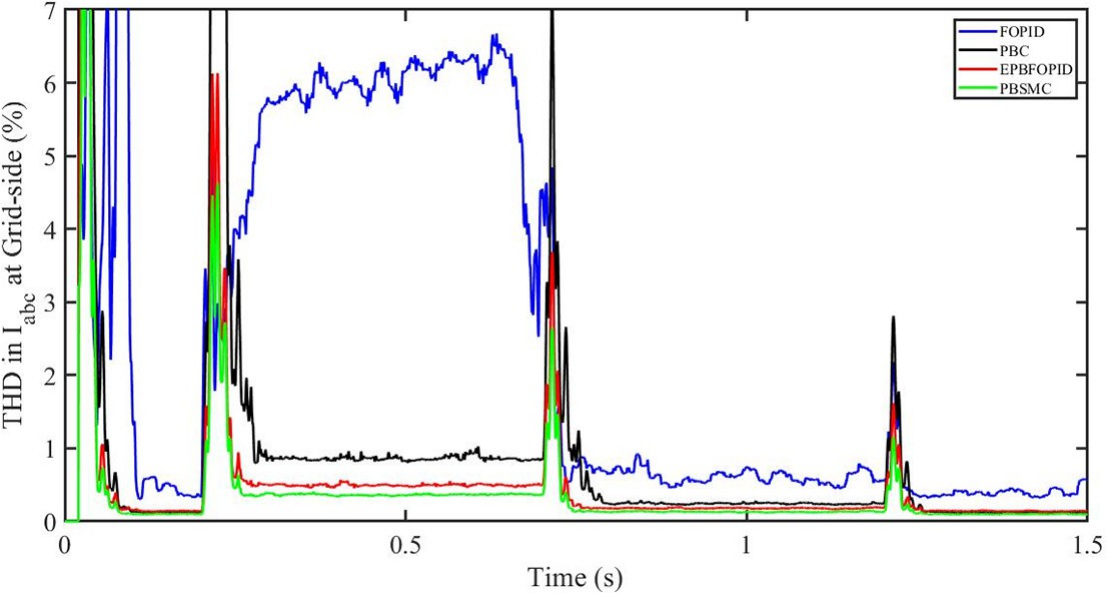
THD in I_abc_ (current) at grid side corresponding to variable irradiance and temperature.

Fig 60 shows the THD in grid side voltage corresponding to the four control systems, FOPID, PBFOPID, PBC and PBSMC with variable irradiance and temperature. As can be seen, proposed PBSMC has least THD. Similarly Fig 61 shows THD in grid side current corresponding to the four control systems. Again as can be seen proposed PBSMC has least THD.

#### 5.5.3 Power grid voltage drop

**Fig 62:**
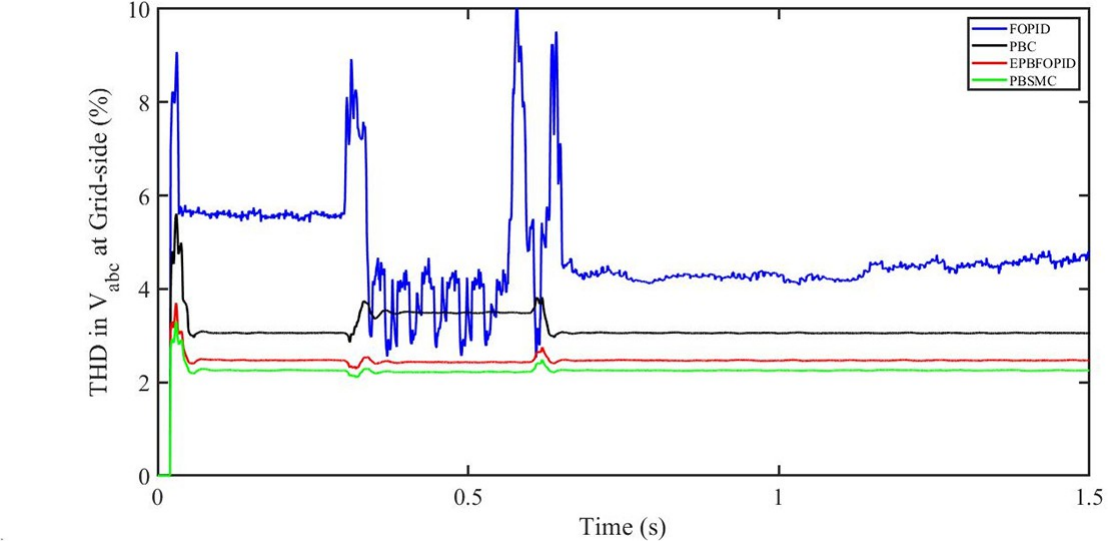
THD in V_abc_ (voltage) corresponding to FRT.

**Fig 63:**
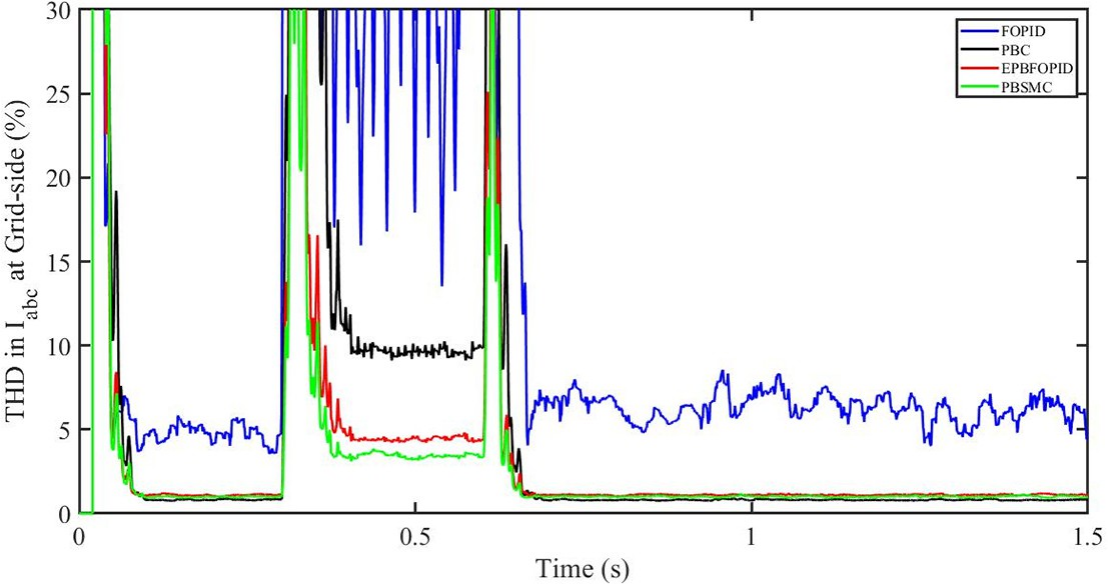
THD in I_abc_ (current) at grid side corresponding to FRT.

Fig 62 shows the THD in grid side voltage corresponding to the four control systems, FOPID, PBFOPID, PBC and PBSMC with power grid voltage drop. As can be seen, proposed PBSMC has least THD. Similarly Fig 63 shows THD in grid side current corresponding to the four control systems. Again as can be seen proposed PBSMC has least THD.

## 6. Conclusion

A novel PBSMC technique for a grid-connected three-phase PV inverter is suggested in this research to capture the maximum possible solar energy under various operating conditions and disturbances. The following is a summary of the findings: (1) a storage function linked with the DC-link voltage, is constructed for the PV system based on the passivity theory, with the physical features of each term extensively researched and evaluated. (2) A unique sliding surface for FOSMC framework is proposed based on R-L theorem (3) The stability and finite time convergence of FOSMC is proved by employing Lyapunov stability criteria. (4) FOSMC is implemented as additional input to the passivized system to reshape the storage function and to significantly increase the robustness of the closed loop system in the presence of PV inverter and its parameter uncertainities. (5) Simulated outcome of case study reveal that PBSMC outperforms FOPID, PBC, and PBFOPID controllers under different atmospheric conditions.

Under solar irradiance change, the tracking time of PV output power is 0.025 seconds due to PBSMC, however FOPID, PBC, EPBFOPID, have failed to converge fully. Similarly, under this condition, the dc link voltage has tracked the reference voltage in 0.05 seconds however the rest of the methods either could not converge, or converge after significant amount of time. Similarly, under solar irradiance and temperature change, the photovoltaic output power has converged in 0.018 seconds, due to PBSMC, however remaining methods fail to converge or track fully. Under same condition, the dc link voltage has minimum tracking error due to PBSMC as compared to the other methods. Under power grid voltage drop, the photovoltaic output power converges to the reference power in 0.1 seconds, whereas other methods failed to converge fully.

## Supporting information

S1 Appendix (DOCX)

## Abbreviations

FOPID: Fractional Order PID
FOSMC: Fractional Order Sliding Mode Control
INC: Incremental conductance
MPPT: Maximum Power Point Tracking
P&O: Perturb and observe
PBC: Passivity based control
PBFOPID: passivity based fractional order PID
PBSMC: Passivity based Fractional order Sliding-Mode controller
PI: Proportional Integral
PID: Proportional integral derivative
R-L: Riemann-Liouville
SMC: Sliding mode control
SPWM: Sinusoidal Pulse Width Modulation
