# Supplementary material for: Passivity-based Rieman Liouville fractional order sliding mode control of three phase inverter in a grid-connected photovoltaic system
Source: PLoS One. 2024 Feb 7;19(2):e0296797. doi: 10.1371/journal.pone.0296797 (PMC10849251; doi:10.1371/journal.pone.0296797)
Supplement: S1 Appendix — (DOCX) [file pone.0296797.s001.docx]

**Appendix**

**SYMBOLS**

| **Symbol** | **Description** |
| --- | --- |
| $I_{pv}$  $I_{ph}$  $V_{dc}$  $I_{dc}$  $I_{sc}$  $v_{d}, v_{q}$  $e_{d}, e_{q}$  $i_{d}, i_{q}$  $\omega$  $R$  $L$  $x$  $y$  $u$  $H\left( x \right)$  $S_{1}, S_{2}$  $e_{1}, e_{2}$  $m_{d}(t)$, $m_{q}(t)$ | PV output current  Cell photo current  PV output voltage  PV inverter’s input current  Cell short circuit current  d-q components of the PV inverter output voltage  d- q components of grid voltage  d-q components of grid current  AC grid frequency  Equivalent line resistance of power grid  Equivalent line inductance of a power grid  State vector  Output of the nonlinear dynamic system  Input of the nonlinear dynamic system  Storage function  Sliding surfaces  Tracking errors  d and q components of modulation signals for Sinusoidal Pulse Width Modulation (SPWM) |
